# Additions and corrections to “Family-group names in Coleoptera (Insecta)”

**DOI:** 10.3897/zookeys.922.46367

**Published:** 2020-03-25

**Authors:** Patrice Bouchard, Yves Bousquet

**Affiliations:** 1 Canadian National Collection of Insects, Arachnids and Nematodes, Agriculture and Agri-Food Canada, 960 Carling Avenue, Ottawa, Ontario, K1A 0C6, Canada Agriculture and Agri-Food Canada Ottawa Canada; 2 Gatineau, Quebec, Canada Unaffiliated Gatineau Canada

**Keywords:** Beetles, family-group name, nomenclature, stem, type genus

## Abstract

Changes to the treatment of Coleoptera family-group names published by Bouchard et al. (2011) are given. These include necessary additions and corrections based on much-appreciated suggestions from our colleagues, as well as our own research. Our ultimate goal is to assemble a complete list of available Coleoptera family-group names published up to the end of 2010 (including information about their spelling, author, year of publication, and type genus).

The following 59 available Coleoptera family-group names are based on type genera not included in Bouchard et al. (2011): Prothydrinae Guignot, 1954, Aulonogyrini Ochs, 1953 (Gyrinidae); Pogonostomini Mandl 1954, Merismoderini Wasmann, 1929, †Escheriidae Kolbe, 1880 (Carabidae); Timarchopsinae Wang, Ponomarenko & Zhang, 2010 (Coptoclavidae); Stictocraniini Jakobson, 1914 (Staphylinidae); Cylindrocaulini Zang, 1905, Kaupiolinae Zang, 1905 (Passalidae); Phaeochroinae Kolbe, 1912 (Hybosoridae); Anthypnidae Chalande, 1884 (Glaphyridae); Comophorini Britton, 1957, Comophini Britton, 1978, Chasmidae Streubel, 1846, Mimelidae Theobald, 1882, Rhepsimidae Streubel, 1846, Ometidae Streubel, 1846, Jumnidae Burmeister, 1842, Evambateidae Gistel, 1856 (Scarabaeidae); Protelmidae Jeannel, 1950 (Byrrhoidea); Pseudeucinetini Csiki, 1924 (Limnichidae); Xylotrogidae Schönfeldt, 1887 (Bostrichidae); †Mesernobiinae Engel, 2010, Fabrasiinae Lawrence & Reichardt, 1966 (Ptinidae); Arhinopini Kirejtshuk & Bouchard, 2018 (Nitidulidae); Hypodacninae Dajoz, 1976, Ceuthocera Mannerheim, 1852 (Cerylonidae); Symbiotinae Joy, 1932 (Endomychidae); Cheilomenini Schilder & Schilder, 1928, Veraniini Schilder & Schilder, 1928 (Coccinellidae); Ennearthroninae Chûjô, 1939 (Ciidae); Curtimordini Odnosum, 2010, Mordellochroini Odnosum, 2010 (Mordellidae); Chanopterinae Borchmann, 1915 (Promecheilidae); Heptaphyllini Prudhomme de Borre, 1886, Olocratarii Baudi di Selve, 1875, Opatrinaires Mulsant & Rey, 1853, Telacianae Poey, 1854, Ancylopominae Pascoe, 1871 (Tenebrionidae); Oxycopiini Arnett, 1984 (Oedemeridae); Eutrypteidae Gistel, 1856 (Mycteridae); Pogonocerinae Iablokoff-Khnzorian, 1985 (Pyrochroidae); Amblyderini Desbrochers des Loges, 1899 (Anthicidae); Trotommideini Pic, 1903 (Scraptiidae); Acmaeopsini Della Beffa, 1915, Trigonarthrini Villiers, 1984, Eunidiini Téocchi, Sudre & Jiroux, 2010 (Cerambycidae); Macropleini Lopatin, 1977, Stenopodiides Horn, 1883, Microrhopalides Horn, 1883, Colaphidae Siegel, 1866, Lexiphanini Wilcox, 1954 (Chrysomelidae); †Medmetrioxenoidesini Legalov, 2010, †Megametrioxenoidesini Legalov, 2010 (Nemonychidae); Myrmecinae Tanner, 1966, Tapinotinae Joy, 1932, Acallinae Joy, 1932, Cycloderini Hoffmann, 1950, Sthereini Hatch, 1971 (Curculionidae).

The following 21 family-group names, listed as unavailable in Bouchard et al. (2011), are determined to be available: Eohomopterinae Wasmann, 1929 (Carabidae); Prosopocoilini Benesh, 1960, Pseudodorcini Benesh, 1960, Rhyssonotini Benesh, 1960 (Lucanidae); Galbini Beaulieu, 1919 (Eucnemidae); Troglopates Mulsant & Rey, 1867 (Melyridae); Hippodamiini Weise, 1885 (Coccinellidae); Micrositates Mulsant & Rey, 1854, Héliopathaires Mulsant & Rey, 1854 (Tenebrionidae); Hypasclerini Arnett, 1984; Oxaciini Arnett, 1984 (Oedemeridae); Stilpnonotinae Borchmann, 1936 (Mycteridae); Trogocryptinae Lawrence, 1991 (Salpingidae); Grammopterini Della Beffa, 1915, Aedilinae Perrier, 1893, Anaesthetinae Perrier, 1893 (Cerambycidae); Physonotitae Spaeth, 1942, Octotomides Horn, 1883 (Chrysomelidae); Sympiezopinorum Faust, 1886, Sueinae Murayama, 1959, Eccoptopterini Kalshoven, 1959 (Curculionidae).

The following names were proposed as new without reference to family-group names based on the same type genus which had been made available at an earlier date: Dineutini Ochs, 1926 (Gyrinidae); Odonteini Shokhin, 2007 (Geotrupidae); Fornaxini Cobos, 1965 (Eucnemidae); Auletobiina Legalov, 2001 (Attelabidae).

The priority of several family-group names, listed as valid in Bouchard et al. (2011), is affected by recent bibliographic discoveries or new nomenclatural interpretations. †Necronectinae Ponomarenko, 1977 is treated as permanently invalid and replaced with †Timarchopsinae Wang, Ponomarenko & Zhang, 2010 (Coptoclavidae); Agathidiini Westwood, 1838 is replaced by the older name Anisotomini Horaninow, 1834 (Staphylinidae); Cyrtoscydmini Schaufuss, 1889 is replaced by the older name Stenichnini Fauvel, 1885 (Staphylinidae); Eremazinae Iablokoff-Khnzorian, 1977 is treated as unavailable and replaced with Eremazinae Stebnicka, 1977 (Scarabaeidae); Coryphocerina Burmeister, 1842 is replaced by the older name Rhomborhinina Westwood, 1842 (Scarabaeidae); Eudysantina Bouchard, Lawrence, Davies & Newton, 2005 is replaced by the older name Dysantina Gebien, 1922 which is not permanently invalid (Tenebrionidae). The names Macraulacinae/-ini Fleutiaux, 1923 (Eucnemidae), Anamorphinae Strohecker, 1953 (Endomychidae), Pachycnemina Laporte, 1840 (Scarabaeidae), Thaumastodinae Champion, 1924 (Limnichidae), Eudicronychinae Girard, 1971 (Elateridae), Trogoxylini Lesne, 1921 (Bostrichidae), Laemophloeidae Ganglbauer, 1899 (Laemophloeidae); Ancitini Aurivillius, 1917 (Cerambycidae) and Tropiphorini Marseul, 1863 (Curculionidae) are threatened by the discovery of older names; Reversal of Precedence (ICZN 1999: Art. 23.9) or an application to the International Commission on Zoological Nomenclature will be necessary to retain usage of the younger synonyms. Reversal of Precedence is used herein to qualify the following family-group names as *nomina protecta*: Murmidiinae Jacquelin du Val, 1858 (Cerylonidae) and Chalepini Weise, 1910 (Chrysomelidae).

The following 17 Coleoptera family-group names (some of which are used as valid) are homonyms of other family-group names in zoology, these cases must be referred to the Commission for a ruling to remove the homonymy: Catiniidae Ponomarenko, 1968 (Catiniidae); Homopterinae Wasmann, 1920, Glyptini Horn, 1881 (Carabidae); Tychini Raffray, 1904, Ocypodina Hatch, 1957 (Staphylinidae); Gonatinae Kuwert, 1891 (Passalidae); Aplonychidae Burmeister, 1855 (Scarabaeidae); Microchaetini Paulus, 1973 (Byrrhidae); Epiphanini Muona, 1993 (Eucnemidae); Limoniina Jakobson, 1913 (Elateridae); Ichthyurini Champion, 1915 (Cantharidae); Decamerinae Crowson, 1964 (Trogossitidae); Trichodidae Streubel, 1839 (Cleridae); Monocorynini Miyatake, 1988 (Coccinellidae); Gastrophysina Kippenberg, 2010, Chorinini Weise, 1923 (Chrysomelidae); Meconemini Pierce, 1930 (Anthribidae).

The following new substitute names are proposed: *Phoroschizus* (to replace *Schizophorus* Ponomarenko, 1968) and Phoroschizidae (to replace Schizophoridae Ponomarenko, 1968); *Mesostyloides* (to replace *Mesostylus* Faust, 1894) and Mesostyloidini (to replace Mesostylini Reitter, 1913).

The following new genus-group name synonyms are proposed [valid names in square brackets]: *Plocastes* Gistel, 1856 [*Aesalus* Fabricius, 1801] (Lucanidae); *Evambates* Gistel, 1856 [*Trichius* Fabricius, 1775] (Scarabaeidae); *Homoeoplastus* Gistel, 1856 [*Byturus* Latreille, 1797] (Byturidae). Two type genera previously treated as preoccupied and invalid, *Heteroscelis* Latreille, 1828 and *Dysantes* Pascoe, 1869 (Tenebrionidae), are determined to be senior homonyms based on bibliographical research. While *Dysantes* is treated as valid here, Reversal of Precedence (ICZN 1999: Art. 23.9) is used to conserve usage of *Anomalipus* Guérin-Méneville, 1831 over *Heteroscelis*.

## Introduction

Nine years have passed since the publication of the catalogue of “Family-group names in Coleoptera” (Bouchard et al. 2011). During this period, we have become aware of a number of available family-group names that were unfortunately omitted at the time. Furthermore, bibliographic and other errors associated with family-group names and their type genera were discovered. The additions and corrections herein focus on available family-group names proposed up to the end of 2010 with the exception of replacement names for names proposed before 2011. Several changes to the classification of Coleoptera have been published in the years since the publication of Bouchard et al. (2011), generally based on new molecular phylogenetic analyses. A thorough summary of these changes is outside of the scope of this article and therefore the classification scheme used in Bouchard et al. (2011) is followed here to maximize uniformity of contents for users. Further action is required to resolve remaining nomenclatural issues involving the Principle of Priority and the Principle of Homonymy (summarized in Appendices 2, 3). These cases are left unresolved so that specialists on the relevant groups can review them and decide on the best course of action. A dagger symbol “†” precedes fossil taxa.

We use the same methods as in Bouchard et al. (2011) although further comments are necessary regarding the treatment of the substantial number of family-group names proposed after 1999 based on the incorrectly formed stem of their type genus. Article 29.4 states that “If after 1999 a new family-group name is based on a generic name which is or ends in a Greek or Latin word or ends in a Greek or Latin suffix, but its derivation does not follow the grammatical procedures of Articles 29.3.1 or 29.3.2, its original spelling must be maintained as the correct original spelling, provided 29.4.1. it has a correctly formed suffix [Art. 29.2], and 29.4.2. its stem is formed from the name of the type genus as though it were an arbitrary combination of letters [Art. 29.3.3].” Following the suggestion of Newton (2017: 4), we have accepted that the best way to promote stability in the long term (especially as several more family-group names proposed since 2011 are based on the incorrectly formed stem of their type genus) is to maintain the spellings as originally proposed when conditions laid out in Art. 29.3.3 are met. In this work we have accordingly reverted back to original spellings for family-group names proposed after 1999 that fall into this category. It may be argued that the spelling of some of these family-group names, as used in our initial work (Bouchard et al. 2011), is in prevailing usage and so is to be maintained (Art. 29.5). However, in all the cases the family-group names as corrected in our initial work have been used by very few authors (as far as we known in two works or fewer) and therefore we do not believe that Article 29.5 should apply here.

## Additions and corrections

**Bibliographic notes**

Throughout the text replace “Acloque, 1896” with “Acloque, 1895”. Acloque’s *Faune de France* was issued in December 1895 (Bousquet 2016: 40), not in 1896 as listed on the title page.

Throughout the text replace “Branden, 1885” with “Branden, 1884”. The separate of Branden’s paper in Volume 29 of the *Annales de la Société Entomologique de Belgique* was issued in 1884, prior to the publication in the journal (Bousquet 2016: 543). The paginations in the separate and the journal article are identical.

Throughout the text replace “Desmarest, 1857” with “Desmarest, 1852”. We follow Bousquet (2016: 142–143) who recommended using “1852” as the correct date of publication for the second part of this work.

Throughout the text replace “Germar, 1824” with “Germar, 1823” (but see exception for the genus *Otiorhynchus* below). Germar’s *Coleopterorum species novae aut minus cognitae*” was issued in 1823, not in 1824 as indicated on the title page (Bousquet 2016: 211; Prena 2018: 327, 342).

Throughout the text replace “Laporte, 1836” with “Laporte, 1838”. Although 1836 is the year given on the title page of volume 4 of the *Revue Entomologique*, this volume was only published in early 1838 (Hayek 1983: 207–208).

Throughout the text replace “Lacordaire, 1856” with “Lacordaire, 1855”. Lacordaire’s third volume of his *Histoire naturelle des insectes* was issued in October 1855, not in 1856 as listed on the title page (Bousquet 2016: 314).

Throughout the text replace “W. S. MacLeay, 1827” with “W. S. MacLeay, 1826”. MacLeay’s *Annulosa. Catalogue of insects*, *collected by Captain King*, *R.N.* was first published in two volumes in 1826 (Bousquet 2016: 353–354).

Throughout the text replace “Pic, 1912” with Pic, 1912a.” The family-group names attributed to “Pic, 1912” in Bouchard et al. (2011) were published in *Coleopterorum Catalogus* (18 October 1912) while Ernobiinae (Pic 1912b: 55), which appeared in Volume 28 of *L’Échange*, was issued earlier in the same year (July 1912).

Throughout the text replace “Reitter, 1909” with “Reitter, 1909a.” The family-group names attributed to “Reitter, 1909” in Bouchard et al. (2011) appeared in the second Theil of Reitter’s “Fauna Germanica”, which should be treated as having been published on December 31, 1909 for nomenclatural purposes (recorded in *Naturae Novitates* in January 1910). The new entry for Tanygnathini Reitter, 1909b (see below) was first proposed in the Coleoptera section (i.e., part 3–4) of “Die Süsswasserfauna Deutschlands: eine Exkursionsfauna” (recorded in *Naturae Novitates* in August 1909).

**Table 1**

Page 8. In Table 1, replace the generic ending from “-*celis*” to “-*scelis*” and the meaning from “spot (Greek)” to “leg (Greek)”.

Page 8. In Table 1, replace the meaning of “-*onyx*” with “claw (Greek)” and that of “-*teles*” with “end, tail (Greek)”.

**Catalogue of Coleoptera family-group names**

**Suborder ARCHOSTEMATA**

Page 95. Below “**Suborder ARCHOSTEMATA**” add:

“**Superfamily Cupedoidea Laporte, 1838**

Cupesidae Laporte, 1838: 56 [stem: *Cuped*-]. Type genus: *Cupes* Fabricius, 1801.” Note. This superfamily is proposed to include the families Crowsoniellidae, Cupedidae, Micromalthidae, Ommatidae, Jurodidae, †Triadocupedidae, †Magnocoleidae, and †Obrieniidae. The suborder ARCHOSTEMATA includes the superfamilies Cupedoidea, †Asiocoleoidea, †Rhombocoleoidea, and †Schizocoleoidea.

Page 97. Replace the valid name “†**Tribe Kenderlykaini Legalov, 2009**” with “†**Tribe Kenderlykanini Legalov, 2009**”.

Page 97. In the entry “Kenderlykanini Legalov, 2009c: 285...” replace the stem with “*Kenderlykan*-” and replace the “Comment” section with “Comment: incorrect original stem formation maintained under Art. 29.4 (should be *Kenderlyka*-).”

Page 97. Move the entries “†**Superfamily Asiocoleoidea Rohdendorf, 1961**”, “†**Superfamily Rhombocoleoidea Rohdendorf, 1961**” and “†**Superfamily Schizophoroidea Ponomarenko, 1968**” and their associated data to below the entry “Obrieniidae Zherikhin and Gratshev, 1994: 51...” Note. The suborder MYXOPHAGA includes only the superfamilies Lepiceroidea Hinton, 1936 (1882) and Sphaeriusoidea Erichson, 1845.

Page 97. Replace the valid name “†**Superfamily Schizophoroidea Ponomarenko, 1968**” with “†**Superfamily Schizocoleoidea Rohdendorf, 1961**”.

Page 97. Replace the entry “Schizophoridae Ponomarenko, 1968: 130…” below the valid name “†**Superfamily Schizocoleoidea Rohdendorf, 1961**” with:

“Schizocoleidae Rohdendorf, 1961: 438 [stem: *Schizocole*-]. Type genus: *Schizocoleus* Rohdendorf, 1961.”

Page 97. Replace the valid name “†**Family Schizophoridae Ponomarenko, 1968**” with “†**Family Phoroschizidae Bouchard and Bousquet, *nomen novum***”.

Page 97. Replace the entry “Schizophoridae Ponomarenko, 1968: 130…” below the valid name “†**Family Schizophoridae Ponomarenko, 1968**” with:

“Schizophoridae Ponomarenko, 1968: 130 [stem: *Schizophor*-]. Type genus: *Schizophorus* Ponomarenko, 1968 [preoccupied genus name, not *Schizophorus* Balashova, 1953 [Trilobita]; syn. of *Phoroschizus* Bouchard and Bousquet, ***nomen novum***. Comment: permanently invalid (Art. 39): based on preoccupied type genus.”

Page 97. Below the entry “Schizophoridae Ponomarenko, 1968: 130…” add:

“Phoroschizidae Bouchard and Bousquet, ***nomen novum*** for Schizophoridae Ponomarenko, 1968 [stem: *Phoroschiz*-]. Type genus: *Phoroschizus* Bouchard and Bousquet, ***nomen novum*** for *Schizophorus* Ponomarenko, 1968.”

Page 97. At the end of the entry “Catiniidae Ponomarenko, 1968: 137…” add “Comment: the family-group name Catiniidae Bocquet and Stock, 1957 (type genus *Catinia* Bocquet and Stock, 1957) is available in Crustacea; this case is to be referred to the Commission to remove the homonymy (Art. 55.3.1); the unnecessary replacement name Coleocatiniidae Ponomarenko and Prokin (2015) is unavailable (Art. 11.7.1.1) since it was not based on an available genus name at the time.”

**Suborder ADEPHAGA**

**Family Tritarsusidae Hong, 2002**

Page 99. Replace the valid name “†**Family Tritarsidae Hong, 2002**” with “†**Family Tritarsusidae Hong, 2002**”.

Page 99. In the entry “Tritarsusidae Hong, 2002: 102...” replace the stem with “*Tritarsus*-” and replace the “Comment” section with “Comment: incorrect original stem formation maintained under Art. 29.4 (should be *Tritars*-).”

**Family Gyrinidae Latreille, 1810**

Page 100. Replace the valid name “**Tribe Enhydrini Régimbart, 1882**” with “**Tribe Enhydrusini Branden, 1882**”.

Page 100. Replace the entry “Enhydrini Régimbart, 1882: 392…” under the valid name “**Tribe Enhydrusini Branden, 1882**” with:

“Enhydrini Branden, 1882: 48 [stem: *Enhydrus*-]. Type genus: *Enhydrus* Laporte, 1834 [placed on the Official List of Generic Names in Zoology (ICZN 1964, 2012a)]. Comment: usage of this name conserved over Dineutini Desmarest, 1851 (Art. 35.5), though considered a junior synonym of Dineutini Desmarest, 1851 by Gustafson and Miller (2013: 81, 95–96); name placed on the Official List of Family-Group Names in Zoology (ICZN 2012a, as “Enhydrusini Régimbart, 1882”).”

Page 100. In the entry “Dineutides Desmarest, 1851: 223…” replace “Type genus: *Dineutes* W. S. MacLeay, 1825.” with “Type genus: *Dineutus* W. S. MacLeay, 1825.”

Page 100. Below the entry “Dineutides Desmarest, 1851: 223…” add:

“Dineutini Ochs, 1926: 63 [stem: *Dineut*-]. Type genus: *Dineutus* W. S. MacLeay, 1825. Comment: family-group name proposed as new without reference to Dineutides Desmarest, 1851 (see Gustafson and Miller 2013).”

Page 100. Replace the valid name “**Subtribe Enhydrina Régimbart, 1882**” with “**Subtribe Enhydrusina Branden, 1882**”.

Page 100. Replace the entry “Enhydrini Régimbart, 1882: 392…” under the valid name “**Subtribe Enhydrusina Branden, 1882**” with:

“Enhydrini Branden, 1882: 48 [stem: *Enhydrus*-]. Type genus: *Enhydrus* Laporte, 1834 [placed on the Official List of Generic Names in Zoology (ICZN 1964, 2012a)]. Comment: correct stem ruled to be *Enhydrus*- to remove homonymy with Enhydrini Gray, 1825 (type genus *Enhydra* Fleming, 1822) in Mammalia and “Enhydrusini Régimbart, 1882” placed on the Official List of Family-Group Names in Zoology (ICZN 2012a); “Enhydrini Régimbart, 1882” deemed to be an incorrect original spelling and placed on the Official Index of Rejected and Invalid Family-Group Names in Zoology (ICZN 2012a); regarding the authorship of this name, Régimbart’s paper was issued by December 1882 while Branden’s was published earlier, before 25 April 1882.”

Page 100. Below the entry “Enhydrini Branden, 1882: 48…” under the valid name “**Subtribe Enhydrusina Branden, 1882**” add:

“Prothydrinae Guignot, 1954: 45 [stem: *Prothydr*-]. Type genus: *Prothydrus* Guignot, 1954.”

Page 100. Below the entry “Gyrinites Latreille, 1810: 141…” under the valid name “**Subtribe Gyrinina Latreille, 1810**” add:

“Aulonogyrini Ochs, 1953: 8 [stem: *Aulonogyr*-]. Type genus: *Aulonogyrus* Motschulsky, 1853.”

Page 100. Replace the valid name “**Tribe Orectochilini Régimbart, 1882**” with “**Tribe Orectochilini Kolbe, 1880**”.

Page 100. Replace “Orectochilini Régimbart, 1882: 391” with “Orectochilini Kolbe, 1880: 264”.

**Family Carabidae Latreille, 1802**

Page 104. Move the entry “Euryodini W. Horn, 1899: 37…” from “**Subtribe Dromicina Thomson, 1859**” to “**Subtribe Cicindelina Latreille, 1802**” below the entry “Cicindeletae Latreille, 1802: 77…” and replace “Type genus: *Euryoda* Lacordaire, 1842 [syn. of *Prothyma* Hope, 1838]” with “Type genus: *Euryoda* Lacordaire, 1842 [syn. of *Heptodonta* Hope, 1838]” Note. As discussed by Bousquet (2002: 22) *Euryoda* Lacordaire, 1842 is an unnecessary replacement name for *Heptodonta* Hope, 1838 and is therefore a junior objective synonym of that taxon.

Page 105. Delete the entry “*Colliurides Motschulsky, 1855: 34…” Note. The genus-group name *Colliuris* used by Latreille (1802) is an incorrect subsequent spelling of *Collyris* Fabricius, 1801, not in prevailing usage. Therefore, Colliurides used by Motschulsky is an incorrect subsequent spelling of Collyridina Brullé, 1834.

Page 105. Below the entry “Ctenostomidae Laporte, 1834b: 38…” add:

“Pogonostomini Mandl 1954: 7 [stem: *Pogonostomat*-]. Type genus: *Pogonostoma* Klug, 1835. Comment: incorrect original stem formation, not in prevailing usage.”

Page 106. In the entry “*Cechenogenici Morawitz, 1889: 40…” replace “Type genus: *Cechenus* Fischer von Waldheim, 1822” with “Type genus: *Cechenus* Fischer von Waldheim, 1822 [preoccupied genus name, not *Cechenus* Illiger, 1807 [Hymenoptera: Braconidae]; syn. of *Cechenochilus* Motschulsky, 1850].”

Page 106. In the entry “Cechenogenici Csiki, 1927: 110…” replace “Type genus: *Cechenus* Fischer von Waldheim, 1822” with “Type genus: *Cechenus* Fischer von Waldheim, 1822 [preoccupied genus name, not *Cechenus* Illiger, 1807 [Hymenoptera: Braconidae]; syn. of *Cechenochilus* Motschulsky, 1850]. Comment: permanently invalid (Art. 39): based on preoccupied type genus.”

Page 116. In the entry “Sinozolini Deuve, 1997…” replace “Type genus: *Sinozolus* Bedel, 1898” with “Type genus: *Sinozolus* Deuve, 1997”.

Page 118. Replace the entry “Nomiidae Gozis, 1875: 3…” with:

“Nomiidae Gozis, 1875: 3 [stem: *Nomius*-]. Type genus: *Nomius* Laporte, 1835 [placed on the Official List of Generic Names in Zoology (ICZN 2011a)]. Comment: Nomiidae Gozis, 1875 placed on the Official Index of Rejected and Invalid Family-Group Names in Zoology (ICZN 2011a), stem emended to *Nomius*- and Nomiusidae Gozis, 1875 placed on the Official List of Family-Group Names in Zoology (ICZN 2011a).”

Page 119. Below the entry “Carabidomemninae Wasmann, 1928: 271…” add:

“Eohomopterinae Wasmann, 1929: 60 [stem: *Eohomopter*-]. Type genus: *Eohomopterus* Wasmann, 1920.”

Page 120. At the end of the entry “Homopterinae Wasmann, 1920: 111…” add “Comment: the family-group name Homopterinae Boisduval, 1852 (type genus *Homoptera* Boisduval, 1852) is available in Lepidoptera; this case is to be referred to the Commission to remove the homonymy (Art. 55.3.1).”

Page 120. Below the entry “Paussili Latreille, 1806: 234…” add:

“Merismoderini Wasmann, 1929: 60 [stem: *Merismoder*-]. Type genus: *Merismoderus* Westwood, 1846 [syn. of *Melanospilus* Westwood, 1846].”

Page 128. At the end of the entry “Glypti G. H. Horn, 1881: 179…” add “Comment: the family-group name Glyptini Cushman and Rohwer, 1920 (type genus *Glypta* Gravenhorst, 1829) is available and used as valid in Hymenoptera: Ichneumonidae; this case is to be referred to the Commission to remove the homonymy (Art. 55.3.1).”

Page 133. In the entry “Callidides Chaudoir, 1873b: 97…” replace “Type genus: *Calleida* Dejean, 1824” with “Type genus: *Calleida* Latreille, 1824”.

Page 138. In the entry “Melanodini Alluaud, 1916: 228…” replace “syn. of *Melanchiton* Basilewsky, 1946.” with “syn. of *Melanchiton* Andrewes, 1940.”

Page 138. In the entry “Melanchitonitae Jeannel, 1948b: 627…” replace “Type genus: *Melanchiton* Basilewsky, 1946” with “Type genus: *Melanchiton* Andrewes, 1940.”

Page 141. In the entry “Meleagrosini Morvan, 2004: 2...” replace the stem with “*Meleagros*-” and replace the “Comment” section with “Comment: incorrect original stem formation maintained under Art. 29.4 (should be *Meleagr*-).”

Page 143. Replace “Stomidae Chaudoir, 1846: 514” with “Stomides Gené, 1839: 50”

Page 145. In the entry “Agronomaeidae Gistel, 1848: [2]…” after “...*Amara* Bonelli, 1810” add “; preoccupied genus name, not *Agronoma* Hübner, 1821 [Lepidoptera]” and at the end of the “Comment” section add “; permanently invalid (Art. 39): based on preoccupied type genus.”

Page 146. Above the valid name “**Family Haliplidae Aubé, 1836**” add:

“**Carabidae *incertae sedis***

†Escheriidae Kolbe, 1880: 266 [stem: *Escheri*-]. Type genus: *Escheria* Heer, 1847.”

**Family Coptoclavidae Ponomarenko, 1961**

Page 147. Replace the valid name “†**Subfamily Necronectinae Ponomarenko, 1977**” with “†**Subfamily Timarchopsinae Wang, Ponomarenko and Zhang, 2010**”.

Page 147. In the entry “Necronectinae Ponomarenko, 1977: 22…” replace “Type genus: *Necronectes* Ponomarenko, 1977 [syn. of *Timarchopsis* Brauer, Redtenbacher and Ganglbauer, 1889]” with “Type genus: *Necronectes* Ponomarenko, 1977 [preoccupied genus name, not *Necronectes* Milne-Edwards, 1881 [Crustacea]; syn. of *Timarchopsis* Brauer, Redtenbacher and Ganglbauer, 1889]” and add “Comment: permanently invalid (Art. 39): based on preoccupied type genus.”

Page 147. Below the entry “Necronectinae Ponomarenko, 1977: 22…” add:

“Timarchopsinae Wang, Ponomarenko and Zhang, 2010: 681 [stem: *Timarchops*-]. Type genus: *Timarchopsis* Brauer, Redtenbacher and Ganglbauer, 1889. Comment: replacement name for Necronectinae Ponomarenko, 1977; incorrect stem formation maintained under Art. 29.4 (should be *Timarchopse*-).”

Page 147. In the entry “Hispanoclavinae Soriano et al., 2007: 527…” replace “Type genus: *Hispanoclava* Soriano, Ponomarenko and Delclos, 2007” with “Type genus: *Hispanoclavina* Soriano, Ponomarenko and Delclòs, 2007” and add “Comment: incorrect stem formation maintained under Art. 29.4 (should be *Hispanoclavin*-).”

**Family Liadytidae Ponomarenko, 1977**

Page 147. In the entry “Liadytidae Ponomarenko, 1977: 37…” replace “Type genus: *Liadytes* Ponomarenko, 1977” with “Type genus: *Liadytes* Ponomarenko, 1963.”

**Family Noteridae Thomson, 1860**

Page 148. Replace the valid name “**Subfamily Notomicrinae Zimmermann, 1919**” with “**Subfamily Notomicrinae Branden, 1884**”.

Page 148. Replace “Notomicrini A. Zimmermann, 1919: 110” with “Notomicrini Branden, 1884: 13”.

**Family Dytiscidae Leach, 1815**

Page 151. In the entry “Bidessini Sharp, 1880: cxlviii…”, replace “Type genus: *Bidessus* Sharp, 1882” with “Type genus: *Bidessus* Sharp, 1880 [for comments regarding problems with the type species of this genus see Bousquet and Bouchard (2018a: 32), Fery and Grygier (2019: 62)].”

Page 152. Replace “Hydrocoptini Branden, 1885: 13” with “Hydrocoptini Kolbe, 1883b: 386.”

Page 152. Replace “Pachydrini Biström et al., 1997: 66” with “Pachydrini Young, 1980: 306.” Note. We do not consider the statement “should probably be placed in a new tribe” used by Young (1980: 306) as evidence of a conditional proposal (Art. 15.1).

**Suborder POLYPHAGA**

**Family Hydrophilidae Latreille, 1802**

Page 157. In the entry “Cyllomina Zaitzev, 1908: 400…” replace “[stem: *Cylomat*-]” to “[stem: *Cylom*-]” and delete the “Comment” section. Note. See comment by Seidel et al. (2016: 161) regarding the stem of the genus *Cyloma* Sharp, 1872.

**Family Histeridae Gyllenhal, 1808**

Page 160. In the entry “Scolytini Jakobson, 1911a: 652…” replace “Type genus: *Scolytus* Müller, 1764 [preoccupied genus name, not *Scolytus* Geoffroy, 1762 [Coleoptera: Curculionidae]; syn. of *Onthophilus* Leach, 1817]. Comment: permanently invalid (Art. 39): based on preoccupied type genus.” with “Type genus: *Scolytus* sensu Jakobson, 1911 [not *Scolytus* Geoffroy, 1762; syn. of *Onthophilus* Leach, 1817]. Comment: based on a misidentified type genus.” Note. Since stability or universality is not threatened, no application to the Commission is needed to suppress this family-group name (Art. 65.2.1).

Page 161. Replace the valid name “**Nymphistrini Tishechkin, 2007**” with “**Nymphisterini Tishechkin, 2007**”.

Page 161. In the entry “Nymphisterini Tishechkin, 2007” replace the stem with “*Nymphister*-” and replace the “Comment” section with “Comment: incorrect original stem formation maintained under Art. 29.4 (should be *Nymphistr*-).” Note. See Newton (2017: 5).

**Family Ptiliidae Erichson, 1845**

Page 166. In the entry “*Pterycini Dybas, 1966: 16, 44…” replace the “Comment” section with “Comment: unavailable family-group name, proposed after 1930 without description or bibliographic reference to such a description (Art. 13.1); incorrect original stem formation, not in prevailing usage; the earlier usage of “pterycine group” by Dybas (1955: 562) is unavailable because it is not a noun (Art. 11.7.1.1); also this name has been used subsequently by Hall (2003: 85) but the name Pterycini has not been made available yet (see Hall 2005: 257).”

**Family Leiodidae Fleming, 1821**

Page 168. Replace the valid name “**Tribe Agathidiini Westwood, 1838**” with “**Tribe Anisotomini Horaninow, 1834**”.

Page 168. Below the entry “*Anisotomidae Stephens, 1828: 99…” add:

“Anisotomidae Horaninow, 1834: 124 [stem: *Anisotom*-]. Type genus: *Anisotoma* Panzer, 1797. Comment: Horaninow (1834) used *Anisotoma* as valid and in the sense of Panzer (1797).”

Page 168. In the entry “Agathidiidae Westwood, 1838: 10…” replace “Type genus: *Agathidium* Illiger, 1798” with “Type genus: *Agathidium* Panzer, 1796.”

Page 168. Delete the entry “Anisotomidae Reitter, 1884: 6…”

Page 169. In the entry “Anisotomidae Erichson, 1845: 41…” delete “; an application will need to be submitted to the Commission to suppress this name for the Principles of Priority and Homonymy (Art. 65.2.1) if Anisotomidae Reitter, 1884 in Leiodinae: Agathidiini is to be used as valid in the future.”

Page 170. Replace the valid names “**Tribe Anemadini Hatch, 1928**” and “**Subtribe Anemadina Hatch, 1928**” with “**Tribe Anemadini Hatch, 1927**” and “**Subtribe Anemadina Hatch, 1927**” respectively.

Page 170. Replace “Anemadina Hatch, 1928: 159” with “Anemadina Hatch, 1927: 14” under the valid name “**Tribe Anemadini Hatch, 1927**” and “**Subtribe Anemadina Hatch, 1927**” respectively.

Page 170. At the end of entry “Anemadinae Jeannel, 1936: 179…” replace “Anemadina Hatch, 1928” with “Anemadina Hatch, 1927”.

Page 171. In the entry “Antroherpona Jeannel, 1910: 25...” replace the stem with “*Anthroherpon*-”.

Page 171. In the entry “Antroherpona Guéorguiev, 1974: 841, in key...” replace the stem with “*Anthroherpon*-” and add at the end of the “Comment” section “incorrect original stem formation, not in prevailing usage.”

**Family Staphylinidae Latreille, 1802**

Page 175. In the entry “Omalidae W. S. MacLeay, 1825…” replace “Type genus: *Omalium* Gravenhorst, 1802” with “Type genus: *Omalium* Gravenhorst, 1802 [placed on the Official List of Generic Names in Zoology (ICZN 2015b)].”

Page 176. In the entry “Omalidae W. S. MacLeay, 1825…” replace “Type genus: *Omalium* Gravenhorst, 1802” with “Type genus: *Omalium* Gravenhorst, 1802 [placed on the Official List of Generic Names in Zoology (ICZN 2015b)].” and at the end of the “Comment” section add: “; name placed on the Official List of Family-Group Names in Zoology (ICZN 2015b; as Omaliidae MacLeay, 1825)”.

Page 178. In the entry “Megarthrini Joy, 1932: 93…” replace “Type genus: *Megarthrus* Curtis, 1829” with “Type genus: *Megarthrus* Stephens, 1829”.

Page 181. In the entry “Neocerini Jeannel, 1954a: 316…” add the following at the end of the “Comment” section: “; the older family-group name Neocerini Salmon, 1941 (type genus *Neocerus* Salmon, 1941, preoccupied genus name, not *Neocerus* Wasmann, 1893) is available though permanently invalid (Art. 39) in Collembola.”

Page 182. Replace the valid name “**Tribe Jubini Raffray, 1904**” with “**Tribe Jubini Raffray, 1898**”.

Page 182. Replace “Jubini Raﬀray, 1904: 489, in key” with “Jubini Raffray, 1898: 199”.

Page 183. Replace the valid name “**Tribe Mayetiini Winkler, 1925**” with “**Tribe Mayetiini Scheerpeltz, 1925**”.

Page 183. Replace “Mayetiini Winkler, 1925: 348” with “Mayetiini Scheerpeltz, 1925: 348.” Note. The section on Staphylinidae, except for the Steninae (pp. 349–357), Euaesthetinae (pp. 357–358) and the genus *Staphylinus* (pp. 381–386), was compiled (“Conscripsit”) by Scheerpeltz (p. 323) and therefore he should be treated as the author of the new taxa in this section.

Page 184. Replace the valid name “**Subtribe Trimiina Bowman, 1934**” with “**Subtribe Trimiina Brendel and Wickham, 1890**”.

Page 184. Replace “Trimii Bowman, 1934: 8” with “Trimiini Brendel and Wickham, 1890: 225”.

Page 184. In the entry “Trimiina Jeannel, 1950a: 139…” replace “without reference to Trimii Bowman, 1934” with “without reference to Trimii Brendel and Wickham, 1890”.

Page 184. Replace the valid names “**Tribe Trogastrini Jeannel, 1949**” and “**Subtribe Trogastrina Jeannel, 1949**” with “**Tribe Trogastrini Brendel and Wickham, 1890**” and “**Subtribe Trogastrina Brendel and Wickham, 1890**” respectively.

Page 184. Below the valid names “**Tribe Trogastrini Jeannel, 1949**” and “**Subtribe Trogastrina Jeannel, 1949**” replace “Trogastrini Jeannel, 1949a: 41, in key” with “Trogasterini Brendel and Wickham, 1890: 225” and add “Comment: incorrect stem formation, not in prevailing usage.”

Page 186. In the entry “Bythinini Raffray, 1890: 83, in key…” under the valid names “**Tribe Bythinini Raffray, 1890**” and “**Subtribe Bythinina Raffray, 1890**” add “Comment: published March 1890; this family-group name was also proposed in the same year by Brendel and Wickham (1890 [June]: 224, as Bythinini).”

Page 188. At the end of the entry “Tychini Raffray, 1904: 490, in key…” add “Comment: junior homonym of Tychinae Dana, 1851 (type genus: *Tyche* Bell, 1835), which has been used as valid in Crustacea: Decapoda recently (e.g., Davie et al. 2015); this case is to be referred to the Commission to remove the homonymy (Art. 55.3.1).”

Page 193. In the entry “Athetae Casey, 1910: 2…” under the valid names “**Tribe Athetini Casey, 1910**” and “**Subtribe Athetina Casey, 1910**” replace the “Comment” section with “Comment: name placed on the Official List of Family-Group Names in Zoology (ICZN 2012e).” Note. The name “Athetae” used earlier by Rambousek (1907: 40) is considered a plural term for subgenera of *Atheta*, Rambousek’s name is therefore unavailable as a family-group name (Art. 11.7.1.2).

Page 193. In the entry “Callicerina Jakobson, 1908: 448…” replace “Type genus: *Callicerus* Gravenhorst, 1802” with “Type genus: *Callicerus* Gravenhorst, 1802 [placed on the Official List of Generic Names in Zoology (ICZN 2012e)].” and replace the “Comment” section with “Comment: following an application by Gusarov (2011) the names Callicerini Jakobson, 1908, Callicerini Horion, 1967 and Callicerini Lohse, 1969, which are junior homonyms of Callicerini Rondani, 1856 (type genus *Callicera* Panzer, 1806) in Diptera: Syrphidae, were suppressed for the purposes of both the Principle of Priority and the Principle of Homonymy and placed on the Official Index of Rejected and Invalid Family-Group Names in Zoology (ICZN 2012e).”

Page 194. At the end of the entry “Geostibae Seevers, 1978: 126…” add “Comment: following an application by Gusarov (2011) the name Geostibina Seevers, 1978 was placed on the Official List of Family-Group Names in Zoology (ICZN 2012e).”

Page 195. In the entries “Corotocini Fenyes, 1918: 61…” replace “Type genus: *Corotoca* Schiødte, 1847” with “Type genus: *Corotoca* Schiødte, 1853”.

Page 197. Replace the valid name “**Cryptonotopseini Pace, 2003**” with “**Cryptonotopsisini Pace, 2003**”.

Page 197. In the entry “Cryptonotopsisini Pace, 2003: 38...” replace the stem with “*Cryptonotopsis*-” and replace the “Comment” section with “Comment: incorrect original stem formation maintained under Art. 29.4 (should be *Cryptonotopse*-).” Note. See Newton (2017: 5).

Page 203. Replace the valid names “**Tribe Mimecitini Wasmann, 1917**” and “**Subtribe Mimecitina Wasmann, 1917**” with “**Tribe Mimecitini Wasmann, 1909**” and “**Subtribe Mimecitina Wasmann, 1909**” respectively.

Page 203. Replace “Mimecitonini Wasmann, 1917: 325” with “Mimecitonini Wasmann, 1909: 55”.

Page 205. Replace the valid name “**Tribe Oxypodinini Fenyes, 1921**” with “**Tribe Oxypodinini Fenyes, 1918**”.

Page 210. In the entry “Prognathites Blanchard, 1845a: 290…” replace “Type genus: *Prognathus* Berthold, 1827” with “Type genus: *Prognathus* Blondel, 1827”.

Page 215. In the entry “Chevrolatini Reitter, 1882c: 142…” replace “Type genus: *Chevrolatia* Jacquelin du Val, 1859” with “Type genus: *Chevrolatia* Jacquelin du Val, 1850”.

Page 215. Replace the valid name “**Tribe Cyrtoscydmini Schaufuss, 1889**” with “**Tribe Stenichnini Fauvel, 1885**” and move names for the entire tribe down to just above the valid name “**Subfamily Steninae MacLeay, 1825**” to maintain alphabetical order of valid tribes. Note. The name Cyrtoscydmini Schaufuss, 1889 was replaced by Glandulariini Schaufuss, 1889 by Newton (2015) but Glandulariini needs to be replaced by Stenichnini Fauvel, 1885 based on priority.

Page 215. Replace “Stenichnini Ganglbauer, 1898: 25” with “Stenichnini Fauvel, 1885: 182” and move the entry “Stenichnini Fauvel, 1885: 182…” above to immediately under the valid name “**Tribe Stenichnini Fauvel, 1885**”.

Page 216. In the entry “Austroaesthetini Cameron, 1944: 69...” replace “as *Austroaesthetus*, unjustifed emendation of genus name not in prevailing usage” with “as *Austroaesthetus*, incorrect subsequent spelling of genus name not in prevailing usage”.

Page 216. Replace the valid name “**Tribe Fenderiini Scheerpeltz, 1974**” with “**Tribe Stictocraniini Jakobson, 1914**”.

Page 216. Above the entry “Fenderiini Scheerpeltz, 1974: 103...” add:

“Stictocraniini Jakobson, 1914: 529 [stem: *Stictocrani*-]. Type genus: *Stictocranius* J. L. LeConte, 1866.” Note. See Newton (2017: 5).

Page 223. In the entry “Philonthidae Kirby, 1837: 91…” replace “Type genus: *Philonthus* Curtis, 1829” with “Type genus: *Philonthus* Stephens, 1829”.

Page 224. In the entry “Ocypina Hatch, 1957: 173, in key…” replace the “Comment” section with “Comment: incorrect original stem formation, not in prevailing usage; usage of the corrected stem *Ocypod*- proposed by Newton and Thayer (1992: 65) creates a homonymy problem with Ocypodidae Rafinesque, 1815 (type genus *Ocypode* Weber, 1795), which has been used as valid in Crustacea (e.g., Naderloo 2017), and therefore this case is to be referred to the Commission to remove the homonymy (Art. 55.3.1).”

Page 224. Replace “Tanygnathinini Reitter, 1909: 105” with “Tanygnathinini Reitter, 1909b: 164”.

**Series SCARABAEIFORMIA**

Page 225. In the entry “Scarabaeïdes Latreille, 1802…” replace “Type genus: *Scarabaeus* Linnaeus, 1758.” with “Type genus: *Scarabaeus* Linnaeus, 1758 [placed on the Official List of Generic Names in Zoology (ICZN 2014)].”

**Family Geotrupidae Latreille, 1802**

Page 226. Replace the valid name “**Tribe Bolbelasmini Nikolajev, 1996**” with “**Tribe Bolbelasmini Iablokoff-Khnzorian, 1977**”.

Page 226. Replace “Bolbelasmini Nikolajev, 1996: 96” with “Bolbelasmini Iablokoff-Khnzorian, 1977: 165”.

Page 227. Replace the valid name “**Tribe Odonteini Shokhin, 2007**” with “**Tribe Odonteini Streubel, 1846**”.

Page 227. Above the entry “Odonteini Shokhin, 2007: 111…” add:

“Odontaeidae Streubel, 1846: 960 [stem: *Odonte*-]. Type genus: *Odonteus* Samouelle, 1819 [as *Odontaeus*, incorrect subsequent spelling of type genus name, not in prevailing usage; placed on the Official List of Generic Names in Zoology (ICZN 2006a)]. Comment: incorrect original stem formation, not in prevailing usage.”

Page 227. In the entry “Odonteini Shokhin, 2007: 111…” replace “…*Bolboceras* Kirby, 1819 was fixed differently by the Commission (ICZN 2006a).” with “…*Bolboceras* Kirby, 1819 was fixed differently by the Commission (ICZN 2006a); family-group name proposed as new without reference to Odonteini Streubel, 1846.”

**Family Passalidae Leach, 1815**

Page 228. In the entry “Ceracupini Boucher, 2006: 319...” replace the stem with “*Ceracup*-” and replace the “Comment” section with “Comment: incorrect original stem formation maintained under Art. 29.4 (should be *Ceracuped*-).”

Page 228. Below the entry “Ceracupedini Boucher, 1006: 319...” add:

“**Tribe Cylindrocaulini Zang, 1905**

Cylindrocaulinae Zang, 1905: 229 [stem: *Cylindrocaul*-]. Type genus: *Cylindrocaulus* Fairmaire, 1880.” Note. Boucher et al. (2017) recently proposed the family-group name Ceracyclini for the genera *Cylindrocaulus* Fairmaire, 1880 and the new fossil genus *Ceracyclus*. However, Cylindrocaulini Zang, 1905 is older and should be used as valid instead of Ceracyclini.

Page 229. At the end of the entry “Gonatinae Kuwert, 1891: 169…” add “Comment: the family-group name Gonatidae Hoyle, 1886 (type genus: *Gonatus* Gray, 1849) is available in Cephalopoda; this case is to be referred to the Commission to remove the homonymy (Art. 55.3.1).”

Page 229. In the entry “Pelopinae Kuwert, 1896: 229…” replace “… [stem: *Pelopid*-]. Type genus: *Pelopides* Kuwert, 1896. Comment: incorrect original stem formation, not in prevailing usage.” with “…[stem: *Pelop*-]. Type genus: *Pelops* Kaup, 1871 [preoccupied genus name, not *Pelops* Koch, 1835 [Acari: Oribatida], syn. of *Protomocoelus* Zhang, 1905]. Comment: the older genus group-name *Pelops* Gistel, 1834 [Coleoptera: Tenebrionidae] was recently treated as a *nomen oblitum* by Bousquet and Bouchard (2017: 132); permanently invalid (Art. 39), based on preoccupied type genus.”

Page 229. Below the entry “Pleurariinae Kuwert, 1896: 224...” add:

“Kaupiolinae Zang, 1905: 227 [stem: *Kaupiol*-]. Type genus: *Kaupiolus* Zang, 1903 [syn. of *Labienus* Kaup, 1871].”

Page 229. Replace “Gnaphalocneminae Gravely, 1914: 194” with “Gnaphalocneminae Heller, 1900: 11”.

**Family Glaresidae Prudhomme de Borre, 1886**

Page 231. Replace the valid name “**Family Glaresidae Kolbe, 1905**” with “**Family Glaresidae Prudhomme de Borre, 1886**”.

Page 231. Replace the entry “Glaresini Kolbe, 1905: 543...” with: “Glaresini Prudhomme de Borre, 1886: 56 [stem: *Glares*-]. Type genus: *Glaresis* Erichson, 1848.”

**Family Lucanidae Latreille, 1804**

Page 232. Below the entry “Aesalidae W. S. MacLeay, 1819: 102…” under the valid name “**Tribe Aesalini MacLeay, 1819**” add:

“Plocasteidae Gistel, 1856a: 365 [stem = *Plocast*-]. Type genus: *Plocastes* Gistel, 1856 [Gistel (1856a: 365) included one species under the generic name *Plocastes*, *scaraboides*, which refers to *Lucanus scarabaeoides* Panzer, 1795 and this species is the type species by monotypy; **syn. nov.** of *Aesalus* Fabricius, 1801]. Comment: incorrect original stem formation, not in prevailing usage.”

Page 234. Replace the entry “*Chalcodinae Didier and Séguy, 1953: 91…” with:

“Chalcodinae Didier and Séguy, 1953: 12, in key, 91 [stem: *Chalcod*-]. Type species: *Chalcodes* H. C. C. Burmeister, 1847.” Note. This family-group name is available since the authors provided a description in the key.

Page 234. In the entry “*Prosopocoilini Benesh, 1960: 50...” remove the asterisk (*) and replace the “Comment” section with “Comment: name proposed after 1930 without description or bibliographic reference to such a description (Art. 13.1), however available because it was used as valid before 2000 as in Klausnitzer (1995: 10, as Prosopocoilini) and was not rejected by an author who, between 1961 and 1999, applied Article 13 of the then current edition of the Code (Art. 13.2.1).”

Page 234. In the entry “*Pseudodorcini Benesh, 1960: 97...” remove the asterisk (*) and replace the “Comment” section with “Comment: name proposed after 1930 without description or bibliographic reference to such a description (Art. 13.1), however available because it was used as valid before 2000 as in Klausnitzer (1995: 10, as Pseudodorcini) and was not rejected by an author who, between 1961 and 1999, applied Article 13 of the then current edition of the Code (Art. 13.2.1).”

Page 234. In the entry “*Rhyssonotini Benesh, 1960: 148...” remove the asterisk (*) and replace the “Comment” section with “Comment: name proposed after 1930 without description or bibliographic reference to such a description (Art. 13.1), however available because it was used as valid before 2000 as in Klausnitzer (1995: 10, as Rhyssonotini) and was not rejected by an author who, between 1961 and 1999, applied Article 13 of the then current edition of the Code (Art. 13.2.1); incorrect original stem formation, not in prevailing usage.”

Page 234. Delete the entry “*Chalcodinae J. P. Lacroix, 1979: 258…”

**Family Ochodaeidae Mulsant and Rey, 1871**

Page 235. Replace the valid names “**Family Ochodaeidae Mulsant and Rey, 1871**”, “**Subfamily Ochodaeinae Mulsant and Rey, 1871**” and “**Tribe Ochodaeini Mulsant and Rey, 1871**” with “**Family Ochodaeidae Streubel, 1846**”, “**Subfamily Ochodaeinae Streubel, 1846**” and “**Tribe Ochodaeini Streubel, 1846**” respectively.

Page 235. Below the valid names “**Family Ochodaeidae Streubel, 1846**”, “**Subfamily Ochodaeinae Streubel, 1846**” and “**Tribe Ochodaeini Streubel, 1846**” replace the entry “Ochodéens Mulsant and Rey, 1871b: 493…” with:

“Ochodaeidae Streubel, 1846: 960 [stem: *Ochodae*-]. Type genus: *Ochodaeus* Dejean, 1821.”

**Family Hybosoridae Erichson, 1847**

Page 237. Replace the entry “Acanthocérides Lacordaire, 1856: 155…” with:

“Acanthoceridae Streubel, 1846: 960 [stem: *Acanthocer*-]. Type genus: *Acanthocerus* W. S. MacLeay, 1819 [preoccupied genus name, not *Acanthocerus* Palisot de Beauvois, 1818 [Hemiptera]; syn. of *Ceratocanthus* A. White, 1842]. Comment: permanently invalid (Art. 39): based on preoccupied type genus.”

Page 237. In the entry “Ceratocanthini Martínez, 1968: 14…” replace “Acanthocerini Lacordaire, 1856” with “Acanthocerini Streubel, 1846”.

Page 237. Below the entry “Hybosoridae Erichson, 1847a: 104...” add: “Phaeochroinen Kolbe, 1912: 153 [stem: *Phaeochro*-]. Type genus: *Phaeochrous* Laporte, 1840. Comment: name listed as “Phaeochroinae” in the Sachregister (p. v), likely written by the editor Karl Grünberg.”

**Family Glaphyridae MacLeay, 1819**

Page 238. Below the entry “Glaphyridae W. S. MacLeay, 1819: 76…” add:

“Anthypnidae Chalande, 1884: 46, 99 [stem= *Anthypn*-]. Type genus: *Anthypna* Eschscholtz, 1818.”

**Family Scarabaeidae Latreille, 1802**

Page 238. In the entry “Scarabaeïdes Latreille, 1802…” replace “Type genus: *Scarabaeus* Linnaeus, 1758.” with “Type genus: *Scarabaeus* Linnaeus, 1758 [placed on the Official List of Generic Names in Zoology (ICZN 2014)].”

Page 239. Replace the valid name “**Subfamily Eremazinae Iablokoff-Khnzorian, 1977**” with “**Subfamily Eremazinae Stebnicka, 1977**”.

Page 239. Replace the entry “Eremazini Iablokoff-Khnzorian, 1977 [3 October]: 168…” with:

“*Eremazini Iablokoff-Khnzorian, 1977 [3 October]: 168 [stem: *Eremaz*-]. Type genus: *Eremazus* Mulsant, 1851. Comment: unavailable family-group name, proposed after 1930 without description or bibliographic reference to such a description (Art. 13.1).” Note. The only descriptive terms provided for this name are “*Bei den Eremizi ist das Metendosternit wie bei den Aphodiinae gebildet, denen sie viel näher stehen als die anderen Aegialitinae, auch ihrer Gesamtbildung nach*” [In the case of the Eremizi the metendosternite is formed as in the Aphodiinae, to which they stand much closer than the other Aegialitinae, also in their general structure]. This statement does not fulfill article 13.1.1, which states “be accompanied by a description or definition that states in words characters that are purported to differentiate the taxon.” Therefore this name is unavailable.

Page 239. In the entry “Eremazina Stebnicka, 1977 [“31 December”]: 412…” delete the “Comment” section.

Page 239. In the entry “Aphodida Leach, 1815: 97…” below the valid names “**Subfamily Aphodiinae Leach, 1815**”, “**Tribe Aphodiini Leach, 1815**” and “**Subtribe Aphodiina Leach, 1815**” replace “Type genus: *Aphodius* Illiger, 1798” with “Type genus: *Aphodius* Hellwig, 1798.” Note. See Alonso-Zarazaga and Krell (2011).

Page 242. In the entry “Scarabaeïdes Latreille, 1802…” replace “Type genus: *Scarabaeus* Linnaeus, 1758.” with “Type genus: *Scarabaeus* Linnaeus, 1758 [placed on the Official List of Generic Names in Zoology (ICZN 2014)].”

Page 243. Replace the valid name “**Tribe Gymnopleurini Lacordaire, 1856**” with “**Tribe Gymnopleurini Streubel, 1846**”.

Page 243. Replace the entry “Gymnopleurides Lacordaire, 1856: 72…” with:

“Gymnopleuridae Streubel, 1846: 961 [stem: *Gymnopleur*-]. Type genus: *Gymnopleurus* Illiger, 1803.”

Page 244. Replace the valid name “**Tribe Onthophagini Burmeister, 1846**” with “**Tribe Onthophagini Streubel, 1846**”.

Page 244. Replace the entry “Onthophagidae H. C. C. Burmeister, 1846: [1]…” with:

“Onthophagidae Streubel, 1846: 961 [stem: *Onthophag*-]. Type genus: *Onthophagus* Latreille, 1802. Comment: published by 13 August 1846; this family-group name was also used in the same year by Burmeister (1846 [by 26 November 1846]: [1], as Onthophagidae).”

Page 245. In the entry “Scarabaeïdes Latreille, 1802…” replace “Type genus: *Scarabaeus* Linnaeus, 1758.” with “Type genus: *Scarabaeus* Linnaeus, 1758 [placed on the Official List of Generic Names in Zoology (ICZN 2014)].” and at the end add: “Comment: name placed on the Official List of Family-Group Names in Zoology (ICZN 2014, as Scarabaeidae Latreille, 1802).”

Page 248. Replace the valid name “**Tribe Comophorinini Britton, 1957**” with “**Tribe Comophini Britton, 1978**”.

Page 248. Replace the entry “Comophorini Britton, 1957: 10…” with the following entries:

“Comophorini Britton, 1957: 10 [stem: *Comophor*-]. Type genus: *Comophorus* Blanchard, 1850 [preoccupied genus name, not *Comophorus* Agassiz, 1846 [Pisces]; syn. of *Comophorina* Strand, 1928]. Comment: permanently invalid (Art. 39): based on preoccupied type genus.

Comophini Britton, 1978: 7 [stem: *Comoph*-]. Type genus: *Comophus* Britton, 1978 [syn. of *Comophorina* Strand, 1928]. Comment: replacement name for Comophorini Britton, 1957 because of the homonymy of the type genus.

Comophorinini Britton, 1987: 761 [stem: *Comophorin*-]. Type genus: *Comophorina* Strand, 1928.”

Page 249. In the entry “Hétéronycides Lacordaire, 1856: 225...” at the end of the “Comment” section add “; the family-group name Heteronychidae André, 1891 (type genus *Heteronyx* Saussure, 1887) is available in Hymenoptera though permanently invalid (based on preoccupied type genus).”

Page 250. Below the entry “Lepisiidae H. C. C. Burmeister, 1844: 166…” add:

“Chasmidae Streubel, 1846: 960 [stem: *Chasm*-]. Type genus: *Chasme* Lepeletier and Audinet-Serville, 1828.”

Page 250. Replace the entry “Lepitrichiden Oken, 1843: 483…” with:

“Lepitrichina Perty, 1840: 933 [stem: *Lepitrich*-]. Type genus: *Lepitrix* Lepeletier and Audinet-Serville, 1828.” Note. The family-group name Lepitrichina Perty, 1840, published by 17 November 1840 (Bousquet 2016: 413), is a senior synonym of Pachycnemina Laporte, 1840, issued by 26 December 1840 (Bousquet 2016: 321). Reversal of Precedence (ICZN 1999: Article 23.9) or an application to the Commission is necessary to conserve usage of Pachycnemina Laporte as valid.

Page 250. Replace the entry “Haplonychidae H. C. C. Burmeister, 1855: 224…” with:

“Haplonychidae H. C. C. Burmeister, 1855: 224 [stem: *Aplonych*-]. Type genus: *Aplonycha* Boisduval, 1835 [as *Haplonycha*, unjustifed emendation of type genus name by Agassiz (1846b: 29)]. Comment: the unjustified emendation *Haplonycha* Agassiz, 1846 is in prevailing usage but attributed to Dejean (1836), not to Boisduval (1835) who first made the name *Aplonycha* available and therefore, Art. 33.2.3.1 (ICZN 1999) cannot be used to consider *Haplonycha* as a justified emendation; incorrect original stem formation, not in prevailing usage; the junior homonym Aplonychini De Stefani, 1908 (type genus *Aplonyx* De Stefani, 1908) is available in Diptera; this case is to be referred to the Commission to remove the homonymy (Art. 55.3.1).”

Page 252. In the entry “Rhizotrogidae H. C. C. Burmeister, 1855: 308…”, replace “Type genus: *Rhizotrogus* Latreille, 1825” with “Type genus: *Rhizotrogus* Berthold, 1827”

Page 257. Below the entry “Phyllurgaeidae Gistel, 1848: [5]…” add:

“Mimelidae Theobald, 1882: 112 [stem = *Mimel*-]. Type genus: *Mimela* Kirby, 1823.”

Page 257. Replace the valid name “**Subtribe Popilliina Ohaus, 1918**” with “**Subtribe Popilliina Ohaus, 1902**”.

Page 257. Replace “Popilliina Ohaus, 1918: 133” with “Popilliidae Ohaus, 1902: 270”

Page 258. Below the entry “Anoplognathidae W. S. MacLeay, 1819: 81…” under the valid name “**Subtribe Anoplognathina MacLeay, 1819**” add:

“Rhepsimidae Streubel, 1846: 960 [stem: *Repsim*-]. Type genus: *Repsimus* W. S. MacLeay, 1819. Comment: incorrect original stem formation, not in prevailing usage.”

Page 259. Replace “Macraspididae H. C. C. Burmeister, 1844: 343” with “Macraspida Perty, 1840: 933”.

Page 259. Below the entry “Pelidnotidae H. C. C. Burmeister, 1844: 388…” add:

“Ometidae Streubel, 1846: 960 [stem: *Omet*-]. Type genus: *Ometis* Latreille, 1829 [syn. of *Lagochile* Hoffmannsegg, 1817].”

Page 260. In the entry “Dynastidae W. S. MacLeay, 1819…” under the valid name “**Subfamily Dynastinae MacLeay, 1819**” replace “Type genus: *Dynastes* W. S. MacLeay, 1819.” with “Type genus: *Dynastes* W. S. MacLeay, 1819 [placed on the Official List of Generic Names in Zoology (ICZN 2014)].” and in the same entry under the valid name “**Tribe Dynastini MacLeay, 1819**” replace “Type genus: *Dynastes* W. S. MacLeay, 1819.” with “Type genus: *Dynastes* W. S. MacLeay, 1819 [placed on the Official List of Generic Names in Zoology (ICZN 2014)].” and at the end add: “Comment: name placed on the Official List of Family-Group Names in Zoology (ICZN 2014).”

Page 260. Replace “Chalepidae H. C. C. Burmeister, 1847: 71” with “Chalepidae Streubel, 1846: 960”.

Page 261. In the entry “Oryctésaires Mulsant, 1842: 372…” replace “Type genus: *Oryctes* Illiger, 1798” with “Type genus: *Oryctes* Hellwig, 1798.” Note. See Alonso-Zarazaga and Krell (2011).

Page 264. In the entry “Aspilina Krikken, 1984: 25, in key…” replace “Type genus: *Aspilus* Westwood in Schaum, 1848” with “Type genus: *Aspilus* Schaum, 1848”

Page 265. In the entry “Spilophorina Krikken, 1984: 25, in key…” replace “Type genus: *Spilophorus* Westwood in Schaum, 1848” with “Type genus: *Spilophorus* Schaum, 1848”.

Page 265. In the entry “Trogodina Krikken, 1984: 27, in key…” replace “Type genus: *Trogodes* Westwood, 1874” with “Type genus: *Trogodes* Boheman, 1851”.

Page 266. Replace the valid name “**Subtribe Coryphocerina Burmeister, 1842**” with “**Subtribe Rhomborhinina Westwood, 1842**”.

Page 266. In the entry “Coryphoceridae H. C. C. Burmeister, 1842: 215...” add “Comment: published by 8 December 1842.”

Page 266. Below the entry “Coryphoceridae H. C. C. Burmeister, 1842: 215...” add:

“Jumnidae H. C. C. Burmeister, 1842: 195 [stem: *Jumn*-]. Type genus: *Jumnos* Saunders, 1839. Comment: published by 8 December 1842; we act as First Revisers (Coryphoceridae H. C. C. Burmeister, 1842 vs Jumnidae H. C. C. Burmeister, 1842) and select Coryphoceridae H. C. C. Burmeister, 1842 to have precedence.

Page 266. Replace the entry “Rhomborrhinae Shoch, 1894: 170…” with:

“Rhomborrhinae Westwood, 1842: 340 [stem: *Rhomborhin*-]. Type genus: *Rhomborhina* Hope, 1837 [as *Rhomborrhina*, incorrect subsequent spelling of the type genus, not in prevailing usage]. Comment: published on 1 January 1842; incorrect original stem formation, not in prevailing usage.” and move entire entry above “Coryphoceridae H. C. C. Burmeister, 1842: 215...” to keep the chronological order. Note. We have decided to use Rhomborhinina Westwood, 1842 as valid (as determined by the Principle of Priority) based on the fact that Cetoniinae taxonomy is in flux and many changes to the classification will be necessary in the future (A. B. T. Smith, pers. comm. September 2019).

Page 271. Below the entry “Paniscidae Gistel, 1848: [5]…” add:

“Evambateidae Gistel, 1856a: 365 [stem = *Evambat*-]. Type genus: *Evambates* Gistel, 1856 [Gistel (1856a: 365) originally included *Scarabaeus fasciatus* Linnaeus, 1758 and *Trichius zonatus* Germar, 1831 in his genus *Evambates*; we hereby select *Scarabaeus fasciatus* Linnaeus, 1758 as the type species of *Evambates* Gistel, 1856; **syn. nov**. of *Trichius* Fabricius, 1775]. Comment: incorrect original stem formation, not in prevailing usage.”

Page 271. In the entry “Myodermini Péringuey, 1907: 313…” replace “[stem: *Myoderm*-]. Type genus: *Myodermum* H. C. C. Burmeister and Schaum, 1840.” with “[stem: *Myodermat*-]. Type genus: *Myoderma* Dejean, 1833. Comment: incorrect original stem formation, not in prevailing usage.” Note. See Bousquet and Bouchard (2013: 38) for comments regarding the correct spelling and authorship of the type genus.

**Family Eucinetidae Lacordaire, 1857**

Page 273. In the entry “Cryptomeridae Broun, 1893: 1358…” replace “Type genus: *Cryptomera* Broun, 1893 [syn. of *Eucinetus* Germar, 1818]” with “Type genus: *Cryptomera* Broun, 1893 [preoccupied genus name, not *Cryptomera* Rafinesque, 1820 [Chilopoda]; syn. of *Eucinetus* Germar, 1818]. Comment: permanently invalid (Art. 39): based on preoccupied type genus.”

**Family Buprestidae Leach, 1815**

Page 278. In the entry “Xenopsina Volkovitsh, 2008: 628...” replace the stem with “*Xenops*-” and replace the “Comment” section with “Comment: incorrect original stem formation maintained under Art. 29.4 (should be *Xenopse*-).”

Page 281. In the entry “Polybothrisidae Gistel, 1848…” replace “Type genus: *Polybothris* Spinola, 1837.” with “Type genus: *Polybothris* Dupont, 1833 [placed on the Official List of Generic Names in Zoology (ICZN 2015a)].”

Page 281. Replace “Capnodina Jakobson, 1913: 779” with “Capnodini Csiki, 1909c: 168”.

Page 285. In the entry “Melobasini Bílý, 2000: 113...” replace the stem with “*Melobas*-” and replace the “Comment” section with “Comment: incorrect original stem formation maintained under Art. 29.4 (should be *Melobase*-).”

Page 288. Under the valid name “**Subtribe Aphanisticina Jacquelin du Val, 1859**” in the entry “Aphanisticites Jacquelin du Val, 1859: 104…” replace “Type genus: *Aphanisticus* Latreille, 1829” with “Type genus: *Aphanisticus* Latreille, 1810.” Note. The genus *Aphanisticus* is often credited in the literature to Latreille (1829a: 448). However, the name was first made available by Latreille (1810: 169).

Page 288. Replace the valid name “**Subtribe Amorphosomatina Majer, 2000**” with “**Subtribe Amorphosomina Majer, 2000**”.

Page 288. In the entry “Amorphosomina Majer, 2000: 210...” replace the stem with “*Amorphosom*-” and replace the “Comment” section with “Comment: incorrect original stem formation maintained under Art. 29.4 (should be *Amorphosomat*-).”

Page 289. Replace the valid name “**Subtribe Clematina Majer, 2000**” with “**Subtribe Clemina Majer, 2000**”.

Page 289. In the entry “Clemina Majer, 2000: 215...” replace the stem with “*Clem*-” and replace the “Comment” section with “Comment: incorrect original stem formation maintained under Art. 29.4 (should be *Clemat*-).”

**Family Byrrhidae Latreille, 1804**

Page 291. Replace “Lioonini Leng, 1920: 193” with “Lioonini Casey, 1912: 59”.

Page 291. At the end of the entry “Microchaetini Paulus, 1973: 353, in key…” add “Comment: the senior homonym Microchaetini Beddard, 1895 (type genus *Microchaetus* Rapp, 1849) in Oligochaeta is currently used as valid (see Plisko 2013: 85); this case is to be referred to the Commission to remove the homonymy (Art. 55.3.1).”

**Superfamily Byrrhoidea Latreille, 1804**

Page 292. Above the valid name “**Family Elmidae Curtis, 1830**” add:

“**Family Protelmidae Jeannel, 1950**

Protelmini Jeannel, 1950: 170, in key [stem: *Protelm*-]. Type genus: *Protelmis* Grouvelle, 1911.”

**Family Elmidae Curtis, 1830**

Page 293. Replace the entry “Limniidae Hope 1838a: 153…” with:

“Limniidae C. G. Thomson, 1859: 21 [stem: *Limni*-]. Type genus: *Limnius* Illiger, 1802.” Note. Limniidae Hope, 1838a: 153 is unavailable since it was not based on a genus used as valid at the time. Hope (1838a) listed *Limnius* (as “*Limneus*”) as a synonym of *Stenelmis* Dufour, 1835.

**Family Limnichidae Erichson, 1846**

Page 295. Above the entry “Thaumastodinae Champion, 1924: 25…” add:

“Pseudeucinetini Csiki, 1924: 14 [stem: *Pseudeucinet*-]. Type genus: *Pseudeucinetus* Heller, 1921. Comment: the names Thaumastodinae Champion and Pseudeucinetini Csiki were published in the same year, Csiki’s publication was issued on 24 February 1924 (verso of title page) while that of Champion was published in the February 1924 issue of the journal *The Entomologist’s Monthly Magazine*; Article 35.5 of the Code (ICZN 1999), proposed by Hernando and Ribera (2016: 32) as a reason to preserve usage of the subfamily name Thaumastodinae, cannot be used in this case, the main reason is that Thaumastodini and Pseudeucinetini are not, and cannot, be used as two separate valid tribes within the subfamily Thaumastodinae since they are based on a single taxonomic entity (i.e., the genus *Pseudeucinetus* of which *Thaumastodus* Champion, 1924 is a junior synonym); an application to the Commission is necessary to maintain usage of Thaumastodinae Champion, 1924 name as valid.”

**Family Eulichadidae Crowson, 1973**

Page 297. Delete the entry “*Lichadiden Kolbe, 1908: 249...”.

Page 298. Replace “Lichadidae Forbes, 1926: 102” with “Lichadidae Csiki, 1902: 191”.

**Superfamily Elateroidea Leach, 1815**

Page 298. In the entry “Elaterides Leach, 1815: 85…” replace the “Comment” section with “Comment: Elateroidea Leach, 1815 given precedence for superfamily name over Cebrionoidea Latreille, 1802 (Art. 35.5; ICZN secretariat pers. comm.).” Note. See Kundrata et al. (2019: 87).

**Family Brachypsectridae Horn, 1881**

Page 299. Replace the valid name “**Family Brachypsectridae LeConte and Horn, 1883**” with “**Family Brachypsectridae Horn, 1881**”.

Page 299. Replace “Brachypsectrini J. L. LeConte and G. H. Horn, 1883: 170” with “Brachypsectrini G. H. Horn, 1881b: 87”.

**Family Eucnemidae Eschscholtz, 1829**

Page 301. Replace the entry “Arhipini Cobos, 1965: 396…” with:

“Arhipini Cobos, 1965: 396 [stem: *Arrhipid*-]. Type genus: *Arrhipis* Bonvouloir, 1871 [as *Arhipis*, unjustified emendation of type genus name by Fleutiaux (1921: 173), not in prevailing usage; preoccupied genus name, not *Arrhipis* Agassiz, 1846, unjustified emendation of *Arripis* Jenyns, 1840 in Pisces]. Comment: permanently invalid (Art. 39): based on preoccupied type genus; incorrect original stem formation, not in prevailing usage.” Note. Since *Arrhipis* Bonvouloir, 1871 is a junior homonym, Reversal of Precedence of the generic name (ICZN 1999: Article 23.9) or an application to the Commission is necessary to conserve usage of this family-group name. Alternatively, a replacement name will be needed.

Page 302. At the end of the entry “Epiphanini Muona, 1993: 45…” add “Comment: the senior homonym Epiphanidae Harring, 1913 (type genus *Epiphanes* Ehrenberg, 1832) in Rotifera is currently used as valid (e.g., Wilts et al. 2012: 180); this case is to be referred to the Commission to remove the homonymy (Art. 55.3.1).”

Page 302. In the entry “Hylocharites Jacquelin du Val, 1859: 119...” replace in the “Comment” section “original vernacular name available (Art. 11.7.2): generally accepted as in Muona (2007: 84, as Hylocharini)” with “original vernacular name available (Art. 11.7.2): first used in latinized form and generally accepted as in Muona (1993: 43)”.

Page 302. In the entries “*Neocharini Muona, 1991a: 167…” and “Neocharini Muona, 1993: 44…” replace “Type genus: *Neocharis* Sharp, 1887” with “Type genus: *Neocharis* Sharp, 1877”.

Page 303. In the entry “*Galbites Blanchard, 1845b: 71…” replace “Comment: original vernacular name unavailable (Art. 11.7.2): not subsequently latinized; if found to be available in the future then permanently invalid (Art. 39): based on preoccupied type genus.” with “Comment: original vernacular name unavailable (Art. 11.7.2): not subsequently latinized and attributed to Blanchard (1845).”

Page 303. Under the entry “*Galbites Blanchard, 1845b: 71…” add:

“Gabini [sic] Beaulieu, 1919: 191 [stem: *Galb*-]. Type genus: *Galba* Latreille, 1829 [preoccupied genus name, not *Galba* Schrank, 1803 [Mollusca]; syn. of *Galbites* Fleutiaux, 1918]. Comment: incorrect original stem formation; permanently invalid (Art. 39): based on preoccupied type genus.”

Page 305. Above the entry “Macraulacinae Fleutiaux, 1923: 304...” under the valid name “**Tribe Macraulacini Fleutiaux, 1923**” add the following entries:

“Fornacini Beaulieu, 1919: 191 [stem: *Fornac*-]. Type genus: *Fornax* Laporte, 1835.

Dromaeolini Beaulieu, 1919: 191 [stem: *Dromaeol*-]. Type genus: *Dromaeolus* Kiesenwetter, 1858.”

Page 305. At the end of the entry “Macraulacinae Fleutiaux, 1923: 304…” under the valid name “**Tribe Macraulacini Fleutiaux, 1923**” add “Comment: Fornacini Beaulieu, 1919 and Dromaeolini Beaulieu, 1919 take precedence over the valid subfamily and tribe names Macraulacinae/-ini Fleutiaux, 1923, an application to the Commission is necessary to preserve usage of Macraulacinae/-ini Fleutiaux, 1923 as valid.”

Page 305. In the entry “Fornaxini Cobos, 1965: 294...” add at the end of the “Comment” section “; family-group name proposed as new without reference to Fornacini Beaulieu, 1919”.

Page 305. Delete the entry “Dromaeolini Leiler, 1976: 48…”

Page 306. In the entry “Nematodini Leiler, 1976: 48…” replace “Type genus: *Nematodes* Berthold, 1827” with “Type genus: *Nematodes* Guérin-Méneville, 1827.” Note. The name *Nematodes* is usually credited to Berthold (1827: 335). However, it was made available first by Guérin-Méneville in Volume 11 (p. 498) of Bory de Saint-Vincent’s *Dictionnaire classique d’histoire naturelle* published on January 1827 (title page). The earliest known date of publication for Berthold’s publication is 22 September 1827 (Bousquet 2016: 73).

**Family Throscidae Laporte, 1840**

Page 306. In the entry “Stereolia Rafinesque, 1815: 112...” replace “[unjustified emendation of *Throscus* Latreille, 1797 not in prevailing usage;” with “[unnecessary replacement name for *Throscus* Latreille, 1797;”.

**Family Elateridae Leach, 1815**

Pages 306–307. In the entry “Elaterides Leach, 1815: 85…” replace the “Comment” section with “Comment: Elateridae Leach, 1815 given precedence for family name over Cebrionidae Latreille, 1802 (Art. 35.5; ICZN secretariat pers. comm.).” Note. See Kundrata et al. (2019: 87).

Page 309. In the entry “Oophoridae Gistel, 1848: [5]…” replace “Type genus: *Oophorus* Eschscholtz, 1833” with “Type genus: *Oophorus* Dejean, 1833.”

Page 310. In the entry “Nyctophyxina C. Costa, 1975: 85…” replace “Type genus: *Nyctophysis* C. Costa, 1975” with “Type genus: *Nyctophyxis* C. Costa, 1975”.

Page 312. In the entry “Campyloxeninae C. Costa, 1975: 114…” replace “Type genus: *Campyloxenus* Fairmaire, 1860” with “Type genus: *Campyloxenus* Fairmaire and Germain, 1860”.

Page 313. At the end of the entry “Limoniina Jakobson, 1913: 755…” add “Comment: the senior homonym Limoniidae/-inae Speiser, 1909 (type genus *Limonia* Meigen, 1803) in Diptera is currently used as valid (e.g., Ribeiro and Amorin 2002: 1); this case is to be referred to the Commission to remove the homonymy (Art. 55.3.1).”

Page 316. Replace the valid name “**Subtribe Loebliquasimusina Schimmel and Tarnawski, 2009**” with “**Subtribe Loebliquasina Schimmel and Tarnawski, 2009**”

Page 316. In the entry “Loebliquasina Schimmel and Tarnawski, 2009: 18...” delete the “Comment” section.

Page 316. Replace the valid name “**Subtribe Striatoquasimusina Schimmel and Tarnawski, 2009**” with “**Subtribe Striatoquasimina Schimmel and Tarnawski, 2009**”.

Page 316. In the entry “Striatoquasina Schimmel and Tarnawski, 2009: 20...” replace the stem with “*Striatoquasim*-”

Page 316. Replace the valid name “**Subtribe Wittmeroquasimusina Schimmel and Tarnawski, 2009**” with “**Subtribe Wittmeroquasimina Schimmel and Tarnawski, 2009**”.

Page 316. In the entry “Wittmeroquasina Schimmel and Tarnawski, 2009: 20...” replace the stem with “*Wittmeroquasim*-” and the type-genus with “*Wittmeroquasimus* Dolin, 1993”.

Page 319. Replace the entry “Dicronychidae Schwarz, 1897: 11…” with:

“Dicronychidae Schwarz, 1897: 11 [stem: *Dicronych*-]. Type genus: *Dicronychus* sensu Laporte, 1840 [not *Dicronychus* Brullé, 1832; syn. of *Eudicronychus* Méquignon, 1931]. Comment: based on a misidentified type genus, name treated here as invalid until an application is submitted to the Commission to suppress it for the Principle of Priority (Art. 65.2.1).”

Page 319. Replace the valid name “**Subfamily Morostomatinae Dolin, 2000**” with “**Subfamily Morostominae Dolin, 2000**”.

Page 319. In the entry “Morostominae Dolin, 2000: 18...” replace the stem with “*Morostom*-” and replace the “Comment” section with “Comment: incorrect original stem formation maintained under Art. 29.4 (should be *Morostomat*-).”

**Family Lycidae Laporte, 1836**

Page 321. In the entry “Libnetinina Bocák and Bocáková, 1990: 652…” replace “Type genus: *Libnetis* C. O. Waterhouse, 1878.” with “Type genus: *Libnetis* C. O. Waterhouse, 1878 [incorrect subsequent spelling of *Libnetus* by C. O. Waterhouse (1879: 77), incorrect subsequent spelling in prevailing usage, treated as correct original spelling (Art. 33.3.1)].”

Page 321 Replace the entry “Lycoprogenthini Bocák and Bocáková, 2008: 709…” with:“Lycoprogenthini Bocák and Bocáková, 2008: 709 [stem: *Lycoprogenth*-]. Type genus: *Lycoprogenthes* Pic, 1915 [incorrect subsequent spelling of *Lycoprogentes* Pic (1915b: 6), incorrect subsequent spelling in prevailing usage, treated as correct original spelling (Art. 33.3.1)].”

Page 322. In the entry “Paralycinae L. N. Medvedev and Kazantsev, 1992: 59…” replace “Type genus: *Paralycus* L. N. Medvedev and Kazantsev, 1992 [syn. of *Lyropaeus* C. O. Waterhouse, 1878].” with “Type genus: *Paralycus* L. N. Medvedev and Kazantsev, 1992 [preoccupied genus name, not *Paralycus* Womersley, 1944 [Acarina]; syn. of *Lyropaeus* C. O. Waterhouse, 1878]. Comment: permanently invalid (Art. 39): based on preoccupied type genus.” Note. The name *Paralycus* Womersley, 1944 is not listed in Neave’s *Nomenclator Zoologicus*. It was made available in Transactions of the Royal Society of South Australia 68: 135.

**Family Lampyridae Rafinesque, 1815**

Page 327. Replace the valid name “**Tribe Cratomorphini Greene, 1948**” with “**Tribe Cratomorphini Olivier, 1911**”.

Page 327. Replace “Cratomorphi Green, 1948: 68, in key” with “Cratomorphinae Olivier, 1911: 75”.

Page 327. In the entry “Dadophorini E. Olivier, 1907: 26…” replace “Type genus: *Dadophora* E. Olivier, 1907” with “Type genus: *Dadophora* Duponchel, 1844.” Note. The genus *Dadophora* is credited to Olivier (1907: 27) in the literature but the generic name was first made available by Duponchel (1844: 574) when he described the species *Dadophora hyalina*.

Page 328. Replace the entry “Photini J. L. LeConte, 1881: 30…” with:“Photini J. L. LeConte, 1881: 30 [stem: *Photin*-]. Type genus: *Photinus* Laporte, 1833 [placed on the Official List of Generic Names in Zoology (ICZN 2018)]. Comment: placed on the Official List of Family-Group Names in Zoology (as Photinini LeConte, 1881) and correct stem of senior homonym (based on the type genus *Photina* Burmeister, 1838) in Mantodea determined to be *Photina*- to remove the homonymy (ICZN 2018).”

Page 329. In the entry “Photurides Lacordaire, 1857: 338…” replace “Type genus: *Photuris* J. L. LeConte, 1851” with “Type genus: *Photuris* Dejean 1833”.

**Family Cantharidae Imhoff, 1856 (1815)**

Page 331. At the end of the entry “Ichthyurini Champion, 1915: 128…” add the following: “Comment: the senior homonym Ichthyurinae Packard, 1895 (type genus *Ichthyura* Hübner, 1819) in Lepidoptera is currently a junior synonym of Pygaerinae Duponchel, 1845 (see Schintlmeister 2013: 17); this case is to be referred to the Commission to remove the homonymy (Art. 55.3.1).”

**Series ELATERIFORMIA**

Page 331. Above the valid name “**Subfamily Cydistinae Paulus, 1972**” add the following: “**Elateriformia *incertae sedis***”. Note. The unassigned family-group taxa Cydistinae Paulus, 1972, Pterotinae LeConte, 1861, Ototretinae McDermott, 1964, Ototretadrilinae Crowson, 1972, and †Lasiosynidae Kirejtshuk, Chang, Ren and Kun, 2010 belong to Elateriformia *incertae sedis* (Lawrence et al. 2010a) and are not actually considered subfamilies of Cantharidae.

**Family Jacobsoniidae Heller, 1926**

Page 333. In the entry “Derolathriinae Sen Gupta, 1979: 692…” replace “Type genus: *Derolathrus* Sharp, 1900” with “Type genus: *Derolathrus* Sharp, 1908”.

**Family Bostrichidae Latreille, 1802**

Page 335. Replace the valid name “**Subfamily Dysidinae Lesne, 1921**” with “**Subfamily Dysidinae Lesne, 1894**”

Page 335. Replace “Dysididae Lesne, 1921b: 286” with “Dysidini Lesne, 1894: 20”

Page 337. Under the entry “Lyctides Billberg, 1820a: 48…” add:

“Xylotrogidae Schönfeldt, 1887: 128 [stem: *Xylotrog*-]. Type genus: *Xylotrogus* Stephens, 1830.”

Page 337. Move the entry “Tristariini Lesne, 1921b: 287…” above the entry “Trogoxylini Lesne, 1921a: 231…” and, at the end of the entry “Tristariini Lesne, 1921b: 287…”, add “Comment: Tristariini was proposed before Trogoxylini since Lesne (1921a: 231) included the proper citation and page of Tristariini; in fact, Lesne unnecessarily changed his name Tristariini to Trogoxylini simply because *Trogoxylon* LeConte, 1862 was older than *Tristaria* Reitter, 1878; however, we recommend that an application be submitted to the Commission to conserve usage of the well-established name Trogoxylini Lesne, 1921.”

**Family Ptinidae Latreille, 1802**

Page 338. Above the valid name “**Subfamily Ptininae Latreille, 1802**” add:

“†**Subfamily Mesernobiinae Engel, 2010**

Mesernobiinae Engel, 2010: 32 [stem: *Mesernobi*-]. Type genus: *Mesernobius* Engel, 2010.”

Page 338. In the entry “Meziini Bellés, 1985: 37, in key…” replace “Type genus: *Mezium* Curtis, 1828” with “Type genus: *Mezium* Samouelle, 1819.” Note. The generic name *Mezium* is credited to Curtis in 1828 in the literature but the name was first made available by Samouelle in 1819 (p. 180) with *Ptinus sulcatus* Fabricius, 1781 as type species by original designation.

Page 338. Replace “Gnostidae Gemminger and Harold, 1868: 700” with “Gnostidae King, 1866: 317”.

Page 339. Replace the entry “Cosmoceroideos Solier, 1849: 476...” with:

“*Cosmoceroideos Solier, 1849: 476 [stem: *Cosmocer*-]. Type genus: *Cosmocerus* Solier, 1849 [preoccupied genus name, not *Cosmocerus* Guérin-Méneville, 1844 [Coleoptera: Cerambycidae]; syn. of *Cerocosmus* Gemminger, 1873]. Comment: original vernacular name unavailable (Art. 11.7.2): subsequently latinized and attributed to Solier (e.g., Lawrence and Newton (1995: 864), as Cosmocerinae) but not generally accepted as valid; if found to be available, permanently invalid (Art. 39): based on preoccupied type genus.”

Page 339. Replace “Ernobiinae Pic, 1912: 12” with “Ernobiinae Pic, 1912b: 55.”

Page 342. In the entry “Fabiinae Martínez and Viana, 1964: 7…” replace “Type genus: *Fabia* Martínez and Viana, 1964” with “Type genus: *Fabia* Martínez and Viana, 1964 [preoccupied genus name, not *Fabia* Dana, 1851 [Crustacea]; syn. of *Fabrasia* Martínez and Viana, 1965]” and add “Comment: permanently invalid (Art. 39): based on preoccupied type genus.”

Page 342. Below the entry “Fabiinae Martínez and Viana, 1964: 7…” add:

“Fabrasiinae Lawrence and Reichardt, 1966: 32 [stem: *Fabrasi*-]. Type genus: *Fabrasia* Martínez and Viana, 1965. Comment: replacement name for Fabiinae Martínez and Viana, 1964 because of the homonymy of the type genus.”

**Family Lymexylidae Fleming, 1821**

Page 342. In the entry “Hylecoeti Germar, 1818: 344…” replace “Type genus: *Hylecoetus* Latreille, 1806” with “Type genus: *Hylecoetus* Latreille, 1806 [syn. of *Elateroides* Schaeffer, 1777].”

**Family Trogossitidae Latreille, 1802**

Page 343. Replace the valid names “**Subfamily Peltinae Latreille, 1806**” and “**Tribe Peltini Latreille, 1806**” with “**Subfamily Peltinae Kirby, 1837**” and “**Tribe Peltini Kirby, 1837**” respectively.

Page 343. Below the valid name “**Subfamily Peltinae Kirby, 1837**” replace the entry “Peltides Latreille, 1806: 8…” with:

“Peltidae Kirby, 1837: 104 [stem: *Pelt*-]. Type genus: *Peltis* Kugelann, 1792 [placed on the Official List of Generic Names in Zoology (ICZN 1994a)].”

Page 343. At the end of the entry “Decamerinae Crowson, 1964: 287…” add “Comment: the junior homonym Decameridae Rasmussen, 1978 (type genus *Decameros* d’Orbigny, 1850) is available in Echinodermata; this case is to be referred to the Commission to remove the homonymy (Art. 55.3.1).”

Page 343. In the entry for “Peltides Latreille, 1806: 8…” under the valid name “**Tribe Peltini Kirby, 1837**” add an asterisk (*) in front of the name and replace the comment with the following: “Comment: family-group name unavailable (Art. 11.7.1.1): not based on a genus used as valid at the time (see Lawrence and Newton 1995: 868).”

Page 343. Below the entry “*Peltides Latreille, 1806: 8…” add:

“Peltidae Kirby, 1837: 104 [stem: *Pelt*-]. Type genus: *Peltis* Kugelann, 1792 [placed on the Official List of Generic Names in Zoology (ICZN 1994a)].”

Page 344. Replace the valid name “**Tribe Calityini Reitter, 1922**” with “**Tribe Calityini Houlbert, 1922**”.

Page 344. Replace “Calityni Reitter, 1922a: 66” with “Calityni Houlbert, 1922a: 103” and add at the beginning of the “Comment” section “issued by 15 July 1922; this family-group name was also used the same year by Reitter (1922a [“31 December 1922”] 66, as Calityni;”.

Page 344. In the entry “Gymnochilides Lacordaire, 1854b: 344…” replace “Type genus: *Gymnochila* Klug, 1844” with “Type genus: *Gymnochila* Erichson, 1844 [syn. of *Gymnocheilis* Dejean, 1835].”

Page 345. Replace the valid name “†**Tribe Lithostomatini Kolibáč and Huang, 2008**” with “†**Tribe Lithostomini Kolibáč and Huang, 2008**”.

Page 345. In the entry “Lithostomini Kolibáč and Huang, 2008: 142...” replace the stem with “*Lithostom*-” and replace the “Comment” section with “Comment: incorrect original stem formation maintained under Art. 29.4 (should be *Lithostomat*-).”

**Family Chaetosomatidae Crowson, 1952**

Page 345. Replace the entry “Chaetosomatidae Crowson, 1952: 66…” with:

“Chaetosomatidae Crowson, 1952: 66 [stem: *Chaetosomat*-]. Type genus: *Chaetosoma* Westwood, 1851 [the senior homonym *Chaetosoma* Chevrolat, 1843 in Cerambycidae was recently suppressed for both the Principle of Priority and the Principle of Homonymy, placed on the Official Index of Rejected and Invalid Generic Names in Zoology, and *Chaetosoma* Westwood, 1851 placed on the Official List of Generic Names in Zoology (ICZN 2011b)]. Comment: the senior homonym Chaetosomatidae Claus, 1872 (type genus *Chaetosoma* Claparède, 1863) in Nematoda was recently suppressed for both the Principle of Priority and the Principle of Homonymy, placed on the Official Index of Rejected and Invalid Family-Group Names in Zoology and Chaetosomatidae Crowson, 1952 placed on the Official List of Family-Group Names in Zoology (ICZN 2011b).”

**Family Cleridae Latreille, 1802**

Page 346. In the entry “Cylidrina Reitter, 1894: 38…” replace “Type genus: *Cylidrus* Latreille, 1829” with “Type genus: *Cylidrus* Latreille, 1817.” Note. The genus-group name *Cylidrus* was first made available by Latreille (1817: 43).

Page 347. Replace the entry “*Trichodites Blanchard, 1845b: 84…” with:

“Trichodidae Streubel, 1839: 136 [stem: *Trichod*-]. Type genus: *Trichodes* Herbst, 1792 [placed on the Official List of Generic Names in Zoology (ICZN 1984a)]. Comment: the younger name originally proposed as Trichodina van der Hoeven, 1849 (type genus *Trichoda* Müller, 1773) is available in Protozoa: Ciliophora; this case is to be referred to the Commission to remove the homonymy (Art. 55.3.1).”

Page 347. Delete the entry “Trichodini Portevin, 1931: 457, in key…”

Page 348. In the entry “Necrobiaeidae Gistel, 1848: [6]...” replace “Type genus: *Necrobia* A. G. Olivier, 1795 [placed on the Official List of Generic Names in Zoology (ICZN 1961c)]” with “Type genus: *Necrobia* Latreille, 1797 [placed on the Official List of Generic Names in Zoology (ICZN 1961c) as “*Necrobia* A. G. Olivier, 1795”].” Note. The genus-group name *Necrobia* was first made available by Latreille (1797: 35), not by Olivier since Olivier’s section Nécrobie [No 76bis] in the fourth volume of his *Entomologie, ou histoire naturelle des insectes* was issued in 1800, not in 1795 (Bousquet 2018: 140).

Page 348. In the entry “Dermestoidini Jakobson, 1911a: 719…” replace “Type genus: *Dermestoides* Schaeffer, 1771” with “Type genus: *Dermestoides* Schaeffer, 1777”.

**Family Melyridae Leach, 1815**

Page 350. In the entry “Pelecophorini Majer, 1987: 797, in key…” replace “Type genus: *Pelecophora* Lepeletier and Audinet-Serville, 1825” with “Type genus: *Pelecophora* Dejean, 1821.”

Page 352. Replace the entry “*Troglopates Mulsant and Rey, 1867b: 281…” with:

“Troglopates Mulsant and Rey, 1867b: 281 [stem: *Troglop*-]. Type genus: *Troglops* Erichson, 1840. Comment: original vernacular name available (Art. 11.7.2): first used in latinized form by Porta (1929: 84) and generally accepted as in Mayor (2007: 451, as Troglopini).”

Page 353. In the entry “Laiina Jakobson, 1911a: 688…” replace “Type genus: *Laius* Guérin-Méneville, 1838” with “Type genus: *Laius* Guérin-Méneville, 1830.” Note. The generic name *Laius* Guérin-Méneville was made available through an illustration (pl. 2, fig. 10) of *Laius cyaneus* issued in November 1830.

**Family Byturidae Gistel, 1848**

Page 354. Below the entry “Byturidae Gistel, 1848: [3]...” under the valid name “**Byturinae Gistel, 1848**” add:

“Homoeoplastidae Gistel, 1856a: 360 [stem = *Homoeoplast*-]. Type genus: *Homoeoplastus* Gistel, 1856 [Gistel (1856a: 360) included two available species under the generic name *Homoeoplastus*, *tomentosus*, which refers to *Dermestes tomentosus* DeGeer, 1774, and *fumatus*, which refers to *Dermestes fumatus* Linnaeus, 1767; we here select *Dermestes tomentosus* DeGeer, 1774 as type species; **syn. nov.** of *Byturus* Latreille, 1797].”

**Family Sphindidae Jacquelin du Val, 1860**

Page 355. In the entry “Odontosphindini Sen Gupta and Crowson, 1979: 180, in key…” replace “Type genus: *Odontosphindus* Sen Gupta and Crowson, 1979” with “Type genus: *Odontosphindus* J. L. LeConte, 1878.”

**Family Erotylidae Latreille, 1802**

Page 358. In the entry “Encaustini Crotch, 1876: 476...” replace in the “Comment” section “published after February 1876” with “published in February 1876”

Page 358. Replace the entry “Triplacinae Erichson, 1847a: 179…” with:

“Triplacina Streubel, 1839: 135 [stem: *Triplac*-]. Type genus: *Triplax* Herbst, 1793.”

**Family Cryptophagidae Kirby, 1826**

Page 360. Replace “Paramecosomina Reitter, 1875: 4” with “Paramecosomini Reitter, 1874: 381”.

Page 360. Replace the valid name “**Tribe Picrotini Crowson, 1980**” with “**Tribe Picrotini Sen Gupta and Crowson, 1971**”.

Page 360. Replace “Picrotini Crowson, 1980: 283” with “Picrotinae Sen Gupta and Crowson, 1971: 30”.

**Family Phalacridae Leach, 1815**

Page 364. In the entry “Eustilbini Guillebeau, 1892: 149…” replace “Type genus: *Eustilbus* Sharp, 1888” with “Type genus: *Eustilbus* Sharp, 1888 [syn. of *Stilbus* Seidlitz, 1872].”

**Family Laemophloeidae Ganglbauer, 1899**

Page 364. Move the entry “Nartheciinae Grouvelle, 1908: 453…” above the entry “Laemophloeini Ganglbauer, 1899: 606…” and replace it with:

“Nartheciini Casey, 1890: 497 [stem: *Nartheci*-]. Type genus: *Narthecius* J. L. LeConte, 1861. Comment: this name is older than the well-established family name Laemophloeidae Ganglbauer, 1899; an application to the Commission is necessary to preserve usage of Laemophloeidae Ganglbauer, 1899 over Nartheciidae Casey, 1890.”

**Family Nitidulidae Latreille, 1802**

Page 367. Replace the valid name “**Arhinini Kirejtshuk, 1987**” with “**Arhinopini Kirejtshuk and Bouchard, 2018**”.

Page 367. In the entry “Arhinini Kirejtshuk, 1987: 63…” replace “Type genus: *Arhina* Murray, 1876” with “Type genus: *Arhina* Murray, 1867 [preoccupied genus name, not *Arhina* Agassiz, 1846 (an unjustified emendation for *Arina* Robineau-Desvoidy, 1830) [Diptera]; syn. of *Arhinops* Kirejtshuk and Bouchard, 2018]. Comment: permanently invalid (Art. 39): based on preoccupied type genus.”

Page 367. Below the entry “Arhinini Kirejtshuk, 1987: 63…” add:

“Arhinopini Kirejtshuk and Bouchard, 2018: 157 [stem: *Arhinop*-]. Type genus: *Arhinops* Kirejtshuk and Bouchard, 2018.”

Page 367. Replace “Pityophagini Reitter, 1891: 163” with “Pityophagini Schilsky, 1888: 60”.

Page 368. Replace “Glischrochilini Iablokoff -Khnzorian, 1966: 314” with “Glischrochilini Chagnon, 1934: 309, in key”.

**Family Cerylonidae Billberg, 1820**

Page 369. Below the entry “Tachyoryctidiini Jeannel and Paulian, 1945…” add:

“Hypodacninae Dajoz, 1976: 184, in key [stem: *Hypodacn*-]. Type genus: *Hypodacne* J. L. LeConte, 1875.”

Page 370. Above the entry “Murmidiides Jacquelin du Val, 1858: 227…” add:

“Ceuthocera Mannerheim, 1852: 383 [stem: *Ceutocer*-]. Type genus: *Ceutocerus* Germar, 1823 [as *Ceuthocerus*, incorrect subsequent spelling of type genus name, not in prevailing usage; syn. of *Murmidius* Leach, 1822]. Comment: incorrect original stem formation, not in prevailing usage; the discovery of this family-group name threatens the well-established name Murmidiinae Jacquelin du Val, 1858; as far as we know, a family-group name based on *Ceutocerus* has not been used as valid after 1899 and we found 25 references, published by at least 10 authors in the immediately preceding 50 years and encompassing a span of not less than 10 years, using the family-group name based on *Murmidius* as valid; therefore Reversal of Precedence (Art. 23.9) is used (see Appendix 1 for supporting references) to qualify Murmidiinae as *nomen protectum* and Ceutocerinae as *nomen oblitum*.

**Family Endomychidae Leach, 1815**

Page 372. Below the valid name “**Subfamily Anamorphinae Strohecker, 1953**” add:

“Symbiotinae Joy, 1932: 558, in key [stem: *Symbiot*-]. Type genus: *Symbiotes* Redtenbacher, 1849.”

Page 372. At the end of the entry “Anamorphini Strohecker, 1953: 15, in key…” add “Comment: the genus *Symbiotes* Redtenbacher, 1849 is currently placed in the subfamily Anamorphinae Strohecker, 1953 (e.g., Tomaszewska 2007: 559), therefore the older name Symbiotinae Joy, 1932 takes precedence over Anamorphinae based on the Principle of Priority; an application to the Commission is necessary to preserve usage of Anamorphinae Strohecker, 1953 as valid.”

Page 373. In the entry “Epipocidae Gorham, 1873: 20…” replace “Type genus: *Epipocus* Germar, 1843” with “Type genus: *Epipocus* Chevrolat, 1836”.

Page 373. In the entry “Corynomalidae Gorham, 1873: 14…” replace “Type genus: *Corynomalus* Gerstaecker, 1857” with “Type genus: *Corynomalus* Chevrolat, 1836”.

**Family Coccinellidae Latreille, 1807**

Page 374. Replace the valid name “**Tribe Serangiini Pope, 1962**” with “**Tribe Serangiini Blackwelder, 1845**”.

Page 374. In the entry “*Serangiini Blackwelder, 1945: 450…” remove the asterisk (*) and replace the “Comment” section with “Comment: name proposed after 1930 without description or bibliographic reference to such a description (Art. 13.1), however available because it was used as valid before 2000 as in Pope (1962: 627, as Serangiini) and was not rejected by an author who, between 1961 and 1999, applied Article 13 of the then current edition of the Code (Art. 13.2.1).”

Page 374. Delete the entry “Serangiini Pope, 1962: 627…”

Page 376. In the entry “*Mysiates Mulsant, 1846: 125…” replace “[stem: *Myzi*-]. Type genus: *Myzia* Mulsant, 1846 [as *Mysia*, alternative original spelling of type genus name; we follow Kovář (2007: 620) in using *Myzia* as the correct spelling for this genus]. Comment: original vernacular name unavailable (Art. 11.7.2): not subsequently latinized; incorrect original stem formation, not in prevailing usage.” with “[stem: *Mysi*-]. Type genus: *Mysia* Mulsant, 1846. Comment: original vernacular name unavailable (Art. 11.7.2): not subsequently latinized.” Note. Mulsant (1846) used two original spellings, *Myzia* and *Mysia*. Subsequently Mulsant (1850: 137) used the spelling *Mysia* and so acted as First Reviser (Art. 24.2.4); *Mysia* is therefore considered the correct original spelling.

Page 377. Below the entry “Synonychini Weise, 1885: 7…” add:

“Hippodamiini Weise, 1885: 11 [stem: *Hippodami*-]. Type genus: *Hippodamia* Chevrolat, 1836.”

Page 377. Above the entry “Anisolemniina Mader, 1954: 93, in key…” add:

“Cheilomenini F. A. Schilder and M. Schilder, 1928: 218 [stem: *Cheilomen*-]. Type genus: *Cheilomenes* Chevrolat, 1836.

Veraniini F. A. Schilder and M. Schilder, 1928: 218 [stem: *Verani*-]. Type genus: *Verania* Mulsant, 1850 [preoccupied genus name, not *Verania* Krohn, 1846 [Mollusca]; syn. of *Micraspis* Chevrolat, 1836]. Comment: permanently invalid (Art. 39): based on preoccupied type genus.”

Page 378. In the entry “Subcoccinellini Jakobson, 1915: 968…” replace “Type genus: *Subcoccinella* Huber, 1841” with “Type genus: *Subcoccinella* Agassiz, 1846.” Note. Huber (1841) proposed the new genus-group name in a vernacular form only (i.e., Subcoccinelle) and therefore the name is unavailable. Agassiz (1846a: 156) is the first who used the name as valid, latinized it, and provided a reference to a previously published description.

Page 379. At the end of the entry “Monocorynini Miyatake, 1988: 28…” add: “Comment: the family group name Monocoryninae Rees, 1956 (type genus *Monocoryne* Broch, 1910) is available in Cnidaria; this case is to be referred to the Commission to remove the homonymy (Art. 55.3.1).”

**Family Corylophidae LeConte, 1852**

Page 382. In the entry “Periptyctinae Ślipiński *et al*., 2001: 312…” replace “Type genus: *Periptyctus* Blackburn, 1825” with “Type genus: *Periptyctus* Blackburn, 1895.”

Page 382. Replace “Clypeastres L. Redtenbacher, 1845: 122” with “Clypeastrina Perty, 1840: 928”.

Page 383. Replace the valid name “**Tribe Sericoderini Matthews, 1888**” with “**Tribe Sericoderini Matthews, 1886**”.

Page 383. Replace “Sericoderina A. Matthews, 1888: 103” with “Sericoderina A. Matthews, 1886: 224”.

**Family Akalyptoischiidae Lord, Hartley, Lawrence, McHugh, Whiting and Miller, 2010**

Page 383. Replace the valid name “**Family Akalyptoischiidae Lord, Hartley, Lawrence, McHugh and Miller, 2010**” with “**Family Akalyptoischiidae Lord, Hartley, Lawrence, McHugh, Whiting and Miller, 2010**”.

**Family Latridiidae Erichson, 1842**

Page 384. Under the valid name “**Family Latridiidae Erichson, 1842**” replace the entry “Lathridien Erichson, 1842: 122…” with:

“Lathridien Erichson, 1842: 122 [stem: *Latridi*-]. Type genus: *Latridius* Herbst, 1793 [placed on the Official List of Generic Names in Zoology (ICZN 2011c)]. Comment: family name Latridiidae given precedence over Corticariidae Curtis, 1829 and other family-group names based on *Corticaria* Marsham, 1802 whenever their type genera are placed in the same family-group taxon, and placed on the Official List of Family-Group Names in Zoology (ICZN 2011c).”

Page 384. Under the valid name “**Subfamily Latridiinae Erichson, 1842**” replace the entry “Lathridien Erichson, 1842: 122…” with:

“Lathridien Erichson, 1842: 122 [stem: *Latridi*-]. Type genus: *Latridius* Herbst, 1793 [placed on the Official List of Generic Names in Zoology (ICZN 2011c)]. Comment: original vernacular name available (Art. 11.7.2): first used in latinized form by L. Redtenbacher (1845: 123, as Lathridii), generally accepted as in Lawrence and Newton (1995: 886, as Latridiidae); incorrect original stem formation, not in prevailing usage.” Note. The name “Latridii” used earlier by Westerhauser (1832: 151) is considered a plural term for the members of the genus *Latridius*, Westerhauser’s name is therefore unavailable as a family-group name (Art. 11.7.1.2).

Page 384. Replace the entry “Corticaridae Curtis, 1829: pl. 283…” with:

“Corticaridae Curtis, 1829: pl. 283 [stem: *Corticari*-]. Type genus: *Corticaria* Marsham, 1802 [placed on the Official List of Generic Names in Zoology (ICZN 2011c)]. Comment: incorrect original stem formation, not in prevailing usage; Corticaridae Curtis, 1829 placed on the Official List of Family-Group Names in Zoology, with the endorsement that it and other family-group names based on *Corticaria* are not to be given priority over Latridiidae Erichson, 1842 and other family-group names based on *Latridius* Herbst, 1793 whenever their type genera are placed in the same family-group taxon (ICZN 2011c).”

Page 384. Replace the entry “*Melanophthalmidae Arnett, 1962b: 835…” with:

“Melanophthalmidae Stickney, 1923: 45 [stem: *Melanophthalm*-]. Type genus: *Melanophthalma* Motschulsky, 1866.”

Page 384. Replace the valid name “†**Subfamily Tetrameropseinae Kirejtshuk and Azar, 2008**” with “†**Subfamily Tetrameropsinae Kirejtshuk and Azar, 2008**”

Page 384. In the entry “Tetrameropsinae Kirejtshuk and Azar, 2008: 36...” replace the stem with “*Tetramerops*-” and replace the “Comment” section with “Comment: incorrect original stem formation maintained under Art. 29.4 (should be *Tetrameropse*-).”

**Family Mycetophagidae Leach, 1815**

Page 385. Replace “Triphyllini Harold, 1880: 757” with “Triphyllidae Crotch, 1873a: 82”.

**Family Ciidae Leach, 1819**

Page 386. Below the entry “Cisidae Leach, 1819: 206…” under the valid name “**Tribe Ciini Leach, 1819**” add:

“Ennearthroninae Chûjô, 1939a: 9 [stem: *Ennearthr*-]. Type genus: *Ennearthron* Mellié, 1847. Comment: Ennearthroninae was also used later in the same year by Chûjô (1939b: 24, in key); incorrect original stem formation, not in prevailing usage.”

**Family Mordellidae Latreille, 1802**

Page 390. Above the valid name “**Tribe Mordellini Latreille, 1802**” add:

“**Tribe Curtimordini Odnosum, 2010**

Curtimordini Odnosum, 2010: 69, 119 [stem: *Curtimord*-]. Type genus: *Curtimorda* Méquignon, 1946.”

Page 390. Above the valid name “**Tribe Reynoldsiellini Franciscolo, 1957**” add:

“**Tribe Mordellochroini Odnosum, 2010**

Mordellochroini Odnosum, 2010: 71, 125 [stem: *Mordellochro*-]. Type genus: *Mordellochroa* Emery, 1876.”

**Family Ripiphoridae Laporte, 1840**

Page 390. Replace the valid name “**Family Ripiphoridae Gemminger, 1870 (1855)**” with “**Family Ripiphoridae Laporte, 1840**”.

Page 390. Replace the entry “Rhipiphoridae Gemminger, 1870: 2117…” with:

“Rhipiphorites Laporte, 1840: 261 [stem: *Ripiphor*-]. Type genus: *Ripiphorus* Bosc, 1791 [as *Rhipiphorus*, unjustified emendation of type genus name by Duméril (1827: 374), not in prevailing usage].”

Page 391. Replace the valid name “**Subfamily Pelecotominae Seidlitz, 1875**” with “**Subfamily Pelecotominae Guérin-Méneville, 1857**”.

Page 391. Replace “Pelecotomini Seidlitz, 1875 [Gatt.]: 104” with “Pelecotomini Guérin-Méneville, 1857: 91”.

Page 391. Replace the valid name “**Subfamily Ripiphorinae Gemminger, 1870 (1855)**” with “**Subfamily Ripiphorinae Laporte, 1840**”.

Page 391. Replace the entry “Rhipiphoridae Gemminger, 1870: 2117…” with:

“Rhipiphorites Laporte, 1840: 261 [stem: *Ripiphor*-]. Type genus: *Ripiphorus* Bosc, 1791 [as *Rhipiphorus*, unjustified emendation of type genus name by Duméril (1827: 374), not in prevailing usage].”

Page 391. Below the valid name “**Tribe Macrosiagonini Heyden, 1908**” delete the entry “Rhipiphorites Laporte, 1840b: 261…”

Page 392. In the entry “Macrosiagonini L. Heyden, 1908: 45…” delete the “Comment” section.

Page 392. Replace the valid name “**Tribe Ripiphorini Gemminger, 1870 (1855)**” with “**Tribe Ripiphorini Laporte, 1840**”.

Page 392. In the entries “*Mioditini A. Costa, 1853: 2…” and “Myoditini Gerstaecker, 1855: 15…” replace “Type genus: *Myodites* Latreille, 1829” with “Type genus: *Myodites* Latreille, 1819”.

Page 392. In the entry “Myoditini Gerstaecker, 1855: 15…” delete the “Comment” section.

Page 392. Replace the entry “Rhipidophoridae Gemminger, 1870: 2117…” with:

“Rhipiphorites Laporte, 1840: 261 [stem: *Ripiphor*-]. Type genus: *Ripiphorus* Bosc, 1791 [as *Rhipiphorus*, unjustified emendation of type genus name by Duméril (1827: 374), not in prevailing usage]. Comment: original vernacular name available (Art. 11.7.2): first used in latinized form by Gerstaecker (1855: 5, as Rhipiphoridum [incorrect stem formation]), generally accepted as in Telnov (2004: 78, as Rhipiphoridae [incorrect stem formation]; an application to the Commission was published recently (Bousquet and Bouchard 2018b) requesting the Commission to rule that Laporte (1840: 261), when proposing the new name Rhipiphorites, used the genus *Ripiphorus* in the currently-accepted sense.” and move entire entry above “*Mioditini A. Costa, 1853: 2...” to keep the chronological order.

**Family Zopheridae Solier, 1834**

Page 394. Replace the valid name “**Tribe Synchitini Erichson, 1845**” with “**Tribe Synchitini L. Redtenbacher, 1845**”.

Page 394. Replace the entry “Synchitini Erichson, 1845: 254…” with:

“Synchitae L. Redtenbacher, 1845: 123 [stem: *Synchit*-]. Type genus: *Synchita* Hellwig, 1792. Comment: published by September 1845; this family-group name was also used in the same year by Erichson (1845 [by 15 October]: 254, as Synchitini).”

Page 394. Replace the entry “Corticini Ganglbauer, 1899: 870…” with:

“Corticides Reitter, 1882a: 116 [stem: *Cortic*-]. Type genus: *Corticus* Germar, 1823.”

Page 395. Replace the entry “Endophloeini Reitter, 1922a: 17...” with:

“Endophloeidae Wollaston, 1868: 264 [stem: *Endophloe*-]. Type genus: *Endophloeus* Dejean, 1834” and move the entry above “Coxelini Seidlitz, 1872 [Gatt.]: 38...” on the previous page.

**Family Promecheilidae Lacordaire, 1859**

Page 396. Below the entry “Proméchilides Lacordaire, 1859: 698...” add:

“Chanopterinae Borchmann, 1915: 47, in key [stem: *Chanopter*-]. Type genus: *Chanopterus* Boheman, 1858.” Note. The genus *Chanopterus* is currently placed in the family Promecheilidae (Lawrence *et al*. 2010b: 563).

**Family Tenebrionidae Latreille, 1802**

Page 398. In the entry “*Phobéliides Lacordaire, 1859: 393…” replace “Type genus: *Phobelius* Blanchard, 1845” with “Type genus: *Phobelius* Blanchard, 1842” Note. The generic name *Phobelius* is credited to Blanchard (1845b: 39) in the literature but it was made available first by Blanchard (1842: pl. 14, fig. 9) when he illustrated the species *Phobelius crenatus*.

Page 398. Below the entry “*Phobéliides Lacordaire, 1859: 393…” add:

“Phobeliides F. Bates, 1890: 76 [stem: *Phobeli*-]. Type genus: *Phobelius* Blanchard, 1842.”

Page 398. Delete the entry “Phobeliina Ardoin, 1961: 33...”

Page 399. Replace the valid name “**Tribe Lupropini Ardoin, 1958**” with “**Tribe Lupropini Lesne, 1926**”.

Page 399. Replace the entry “Lupropsini Ardoin, 1958: 59…” with:

“Lypropini Lesne, 1926: 68 [stem: *Luprop*-]. Type genus: *Luprops* Hope, 1833 [as *Lyprops*, incorrect subsequent spelling not in prevailing usage]. Comment: incorrect original stem formation, not in prevailing usage.”

Page 404. Replace “Eusatti Doyen, 1984: 11” with “Eusatti Casey, 1908: 55”.

Page 408. In the entry “Nyctélites Solier, 1834…” replace “Type genus: *Nyctelia* Latreille, 1825” with “Type genus: *Nyctelia* Berthold, 1827”.

Page 409. In the entry “Leucolaephusini Pierre, 1961: 558…” replace “Type genus: *Leucolaephus* Lucas, 1859. Comment: incorrect original stem formation, not in prevailing usage.” with “Type genus: *Leucolaephus* Lucas, 1859 [*Leucolaephus* is an incorrect subsequent spelling of the original spelling *Leucoloephus*, in prevailing usage and so deemed to be the correct original spelling (Art. 33.3.1)]. Comment: incorrect original stem formation, not in prevailing usage.”

Page 415. Below the entry “Eutomides Lacordaire, 1865: 369...” add:

“Heptaphyllini Prudhomme de Borre, 1886: 56 [stem: *Heptaphyll*-]. Type genus: *Heptaphylla* Friedenreich, 1883 [syn. of *Rhipidandrus* J. L. LeConte, 1862].”

Page 416. In the entry “Heleadae Fleming, 1821: 51” replace “Type genus: *Helea* Latreille, 1816” with “Type genus: *Helea* Latreille, 1804”.

Page 417. In the entry “Hypulia Rafinesque, 1815…” replace “[syn. of *Helops* Fabricius, 1775].” with “[preoccupied genus name, not *Hypulus* Paykull, 1798 [Coleoptera: Melandryidae]; syn. of *Helops* Fabricius, 1775]. Comment: permanently invalid (Art. 39): based on preoccupied type genus.”

Page 421. In the entry “*Omocratates Mulsant and Rey, 1854: 266, in key…” replace “syn. of *Phylan* Dejean, 1821” with “syn. of *Phylan* Sturm, 1826”.

Page 421. In the entry “Pandarites Mulsant & Rey, 1854: 153…” replace “[stem: *Pandar*-]” with “[stem: *Dendar*-]”.

Page 421. Replace the entry “*Micrositates Mulsant and Rey, 1854: 274…” with:

“Micrositates Mulsant and Rey, 1854: 274 [stem: *Microsit*-]. Type genus: *Micrositus* Mulsant and Rey, 1854. Comment: original vernacular name available (Art. 11.7.2): first used in latinized form and generally accepted as in Baudi di Selve (1875: 158, as Micrositarii).”

Page 422. Replace the entry “*Héliopathaires Mulsant and Rey, 1854: 265…” with:

“Héliopathaires Mulsant and Rey, 1854: 265 [stem: *Heliopat*-]. Type genus: *Heliopates* Dejean, 1834 [as *Heliopathes*, incorrect subsequent spelling of type genus name, not in prevailing usage]. Comment: original vernacular name available (Art. 11.7.2): first used in latinized form and generally accepted as in Baudi di Selve (1875: 159, as Heliopatharii).”

Page 422. Above the entry “Bioplanina A. N. Reichardt, 1936: 24…” add:

“Olocratarii Baudi di Selve, 1875: 158 [stem = *Olocrat*-]. Type genus: *Olocrates* Mulsant, 1854 [syn. of *Phylan* Sturm, 1826].” Note. *Olocrates* Mulsant, 1854 (p. 383) was proposed in the *Errata and addenda* of Mulsant’s work as a replacement name for *Omocrates* Mulsant, 1854 (p. 150) a junior homonym of *Omocrates* H. C. C. Burmeister, 1844 [Coleoptera: Scarabaeidae].

Page 422. In the entry “Phylacides Lacordaire, 1859: 270…” replace “Type genus: *Phylax* Brullé, 1832 [syn. of *Dendarus* Dejean, 1821].” with “Type genus: *Phylax* Brullé, 1832 [unnecessary replacement name for *Phylan* Sturm, 1826; syn. of *Phylan* Sturm, 1826].” Note. It is evident, particularly from the footnote attached to the generic name, that *Phylax* Brullé, 1832 is an unnecessary replacement name for *Phylan* Sturm, 1826. This means that *Phylax* Brullé is not a junior synonym of *Dendarus* Dejean, 1821 but a junior synonym of *Phylan* Sturm, 1826.

Page 422. Replace “Psectropini Kaszab, 1941: 33” with “Psectropini Kaszab, 1940: 141”.

Page 422. In the entry “Leichenaires Mulsant, 1854: 179…” replace “Type genus: *Leichenum* Blanchard, 1845” with “Type genus: *Leichenum* Dejean, 1834”.

Page 423. Replace the entry “*Hétéroscélites Solier, 1836: 502…” with:

“*Hétéroscélites Solier, 1836: 502 [stem: *Heteroscelid*-]. Type genus: *Heteroscelis* Latreille, 1828 [*nomen oblitum*; this genus name is a senior subjective synonym of *Anomalipus* Guérin-Méneville, 1831 *nomen protectum*; we provide references to support the conservation of *Anomalipus* Guérin-Méneville, 1831 as the valid name for this genus (Art. 23.9.1) (see Appendix 1)]. Comment: original vernacular name unavailable (Art. 11.7.2): not subsequently latinized; incorrect original stem formation, not in prevailing usage.” Note. The Coleoptera genus-group name *Heteroscelis* Latreille (1828: 574), previously credited to Latreille (1829b: 18), is the senior homonym of the name *Heteroscelis* Latreille (1829b: 194) in Hemiptera, and not the reverse as has been accepted in the literature.

Page 423. Below the entry “Trigonopaires Mulsant and Rey, 1853: 104…” add:

“Opatrinaires Mulsant and Rey, 1853: 294 [stem = *Opatrin*-]. Type genus: *Opatrinus* Dejean, 1821. Comment: original vernacular name available (Art. 11.7.2): first used in latinized form and generally accepted as in Giebel (1855: 90, as Opatrinini).”

Page 425. Replace the valid name “**Subtribe Eudysantina Bouchard, Lawrence, Davies and Newton, 2005**” with “**Subtribe Dysantina Gebien, 1922**”.

Page 425. Replace the entry “Dysantinae Gebien, 1922: 289…” with:

“Dysantinae Gebien, 1922: 289 [stem: *Dysant*-]. Type genus: *Dysantes* Pascoe, 1869.” Note. The genus *Dysantes* Pascoe (1869a: 31) was first made available in 1869 [on 1 January], not in 1871 as previously noted. The name is a senior homonym of the Hymenoptera name *Dysantes* Förster, 1869 [May] which has been incorrectly dated 1868 in some works.

Page 425. In the entry “Eudysantina Bouchard et al., 2005: 508…” replace the “Comment” section with “Comment: unnecessary replacement name for Dysantina Gebien, 1922”.

Page 427. In the entry “Mycetocharisidae Gistel, 1848: [10]…” replace “Type genus: *Mycetochara* Berthold, 1827” with “Type genus: *Mycetochara* Guérin-Méneville, 1827”. Note. The name *Mycetochara* is usually credited to Berthold (1827: 371). However the name was made available first by Guérin-Méneville in Volume 11 (p. 346) of Bory de Saint-Vincent’s *Dictionnaire classique d’histoire naturelle* published on January 1827 (see Bousquet and Bouchard 2016: 138).

Page 428. Above the entry “Omophliens Mulsant, 1856a: 65…” add:

“Telacianae Poey, 1854: 322 [stem: *Telac*-]. Type genus: *Telacis* Poey, 1854 [syn. of *Cteniopus* Solier, 1835]. Comment: incorrect original stem formation, not in prevailing usage.”

Page 428. Replace “Petriidae Semenov, 1893b: 359” with “Petriidae Semenov, 1894a: 359”

Page 433. In the entry “Stenochiadae Kirby, 1837: 238…” replace “Type genus: *Stenochia* Kirby, 1819.” with “Type genus: *Stenochia* Kirby, 1819 [syn. of *Strongylium* Kirby, 1819].”

Page 433. Below the heading “Tenebrionidae *incertae sedis*” add:

“Ancylopominae Pascoe, 1871: 354 [stem: *Ancylopomat*-]. Type genus: *Ancylopoma* Pascoe, 1871. Comment: incorrect original stem formation, not in prevailing usage.” Note. The systematic position of the genus *Ancylopoma* has not been studied after its description except for comments by Bates (1872: 97) who mentioned that “it should be placed after *Anaedus*, Blanch.” It was included in Heterotarsinae by Gebien (1911: 472) and Blackwelder (1945: 537) along with *Anaedus*. Recently, the genus *Anaedus* has been included in the tribe Goniaderini Lacordaire, 1859 (e.g., Doyen and Tschinkel 1982: 182, Bousquet et al. 2018: 29) or Lupropini Ardoin, 1958 (e.g., Schawaller 2011: 271).

**Family Oedemeridae Latreille, 1810**

Page 435. In the entry “Ascleraeidae Gistel, 1848: [11]…” replace “Type genus: *Asclera* Stephens, 1839” with “Type genus: *Asclera* Dejean, 1834 [syn. of *Ischnomera* Stephens, 1832]”.

Page 435. In the entry “Ganglbaueriidae Semenov, 1894: 450…” replace “[stem: *Ganglebaueri*-]” with “[stem: *Ganglbaueri*-].”

Page 435. Below the entry “*Hypasclerini Macnamara, 1971: 164, in key…” add:

“Oxaciini Arnett, 1984: 4, in key [stem: *Oxacid*-]. Type genus: *Oxacis* J. L. LeConte, 1866. Comment: incorrect original stem formation, not in prevailing usage.” Note. The genus *Oxacis* is currently placed in the tribe Asclerini Gistel, 1848 (e.g., Kriska 2002: 518).

Oxycopiini Arnett, 1984: 4, in key [stem: *Oxycopid*-]. Type genus: *Oxycopis* Arnett, 1951. Comment: incorrect original stem formation, not in prevailing usage.” Note. The genus *Oxycopis* is currently placed in the tribe Asclerini Gistel, 1848 (e.g., Kriska 2002: 518).”

Page 435. Replace the entry “*Hypasclerini Švihla, 1986: 161…” with:

“Hypasclerini Arnett, 1984: 4, in key [stem: *Hypascler*-]. Type genus: *Hypasclera* Kirsch, 1866.”

Page 435. Delete the entry “*Oxacini Švihla, 1986: 161…”

**Family Meloidae Gyllenhal, 1810**

Page 437. In the entry “Spasticina Kaszab, 1959: 72…” replace “Type genus: *Spastica* Lacordaire, 1859” with “Type genus: *Spastica* Lucas, 1859.” Note. The genus *Spastica* is currently credited to Lacordaire (1859 [issued by 27 June]: 679). However, the name was first treated as a junior synonym of *Gnathium* Kirby, 1819 by Lucas (1859 [issued by 7 March 1859]: 147). Since the name has been treated as an available name by Lacordaire (1859: 679), it is made available but dates from its publication as a synonym (Art. 11.6.1).

Page 439. Replace the valid name “**Tribe Lyttini Solier, 1851**” with “**Tribe Lyttini Streubel, 1846**”.

Page 439. Delete the entry “*Lyttes Motschulsky, 1849: 59…”

Page 439. Replace the entry “Lyttoides Solier, 1851: 278…” with:

“Lyttidae Streubel, 1846: 961 [stem: *Lytt*-]. Type genus: *Lytta* Fabricius, 1775. Comment: although this is not the oldest name for the tribe, we recommend that an application be submitted to the Commission to suppress Cantharini Latreille, 1802 because it is based on a misidentifed type genus (Art. 65.2.1).”

**Family Mycteridae Perty, 1840**

Page 443. Replace the valid names “**Family Mycteridae Oken, 1843**” and “**Subfamily Mycterinae Oken, 1843**” with “**Family Mycteridae Perty, 1840**” and “**Subfamily Mycterinae Perty, 1840**” respectively.

Page 443. Below the valid names “**Family Mycteridae Perty, 1840**” and “**Subfamily Mycterinae Perty, 1840**” replace “Mycteriden Oken, 1843: 484” with “Mycterina Perty, 1840: 919” and delete the “Comment” section.

Page 443. Below the entry “Artaxidae Gistel, 1848: [8]…” add:

“Eutrypteidae Gistel, 1856a: 375 [stem = *Eutrypt*-]. Type genus: *Eutryptes* Gistel, 1856 [subgenus of *Mycterus* Clairville, 1798]. Comment: incorrect original stem formation, not in prevailing usage.”

Page 443. Replace the entry “*Stilpnonotinae Blackwelder, 1945: 503…” with:

“Stilpnonotinae Borchmann, 1936: 13, in key [stem: *Stilpnonot*-]. Type genus: *Stilpnonotus* Gray, 1832.”

**Family Pythidae Solier, 1834**

Page 444. Replace “Osphyoplesiini Reitter, 1917: 59, in key” with “Osphyoplesiini A. Winkler, 1915: 333”.

**Family Pyrochroidae Latreille, 1806**

Page 445. Above the valid name “**Subfamily Agnathinae Lacordaire, 1859**” add:

“**Subfamily Pogonocerinae Iablokoff-Khnzorian, 1985.**

Pogonocerinae Iablokoff-Khnzorian, 1985: 197 [stem: *Pogonocer*-]. Type genus: *Pogonocerus* Fischer von Waldheim, 1812.”

Page 445. Replace “Elacatidae Cockerell, 1906: 242” with “Elacatidae Reitter, 1879: 212”.

**Family Salpingidae Leach, 1815**

Page 446. In the entry “*Trogocryptinae Crowson, 1953: 51…” delete “; this name has been used subsequently, e.g., Lawrence (1977: 43, 1980: 307), Lawrence and Newton (1995: 900), but it has not been made available”.

Page 446. Below the entry “*Trogocryptinae Crowson, 1953: 51…” add:

“Trogocryptinae Lawrence, 1991: 260, 294, in key [stem: *Trogocrypt*-]. Type genus: *Trogocryptus* Sharp, 1900.”

Page 446. In the entries “*Rhinosimites Solier, 1834: 496…” and “Rhinosimidae Hope, 1840a: 134…” replace “Type genus: *Rhinosimus* Latreille, 1802” with “Type genus: *Rhinosimus* Latreille, 1802 [syn. of *Salpingus* Illiger, 1802].”

Page 446. Replace “Lissodemina Seidlitz, 1917b: 422” with “Lissodemina Seidlitz, 1916b: 337”.

**Family Anthicidae Latreille, 1819**

Pages 446 and 448 (twice). Replace “Anthicites Latreille, 1819: 363” with “Anthicites Latreille, 1819: 437” and replace “Type genus: *Anthicus* Paykull, 1798.” with “Type genus: *Anthicus* Paykull, 1798 [placed on the Official List of Generic Names in Zoology (ICZN 2017)].”

Page 447. Replace the valid name “**Tribe Mitraelabrini Abdullah, 1969**” with “**Tribe Mitraelabrini Abdullah and Abdullah, 1968**”.

Page 447. Replace “Mitraelabrini M. Abdullah, 1969a: 350” with “Mitraelabrini M. Abdullah and A. Abdullah, 1968: 73, in key”.

Page 447. Replace the valid name “†**Tribe Camelomorphini Kirejtshuk and Azar, 2008**” with “†**Tribe Camelomorphini Kirejtshuk, Azar and Telnov, 2008**”

Page 447. Replace “Camelomorphini Kirejtshuk and Azar, 2008: 40” with “Camelomorphini Kirejtshuk, Azar and Telnov, 2008: 40” and “Type genus: *Camelomorpha* Kirejtshuk and Azar, 2008” with “Type genus: *Camelomorpha* Kirejtshuk, Azar and Telnov, 2008”

Page 447. Replace the valid name “**Subfamily Lemodinae Lawrence and Britton, 1991**” with “**Subfamily Lemodinae Matthews, 1987**”

Page 447. Replace “Lemodinae Lawrence and Britton, 1991: 603, in key” with “Lemodinae Matthews, 1987: 40”

Page 448. Below the entry “Anthicini Latreille, 1819: 363...” under the valid name “**Tribe Anthicini Latreille, 1819**” add:

“Amblyderini Desbrochers des Loges, 1899: 28 [stem: *Amblyder*-]. Type genus: *Amblyderus* LaFerté-Sénectère, 1847.”

Page 448. In the entry “Microhorini Bonadona, 1974: 110, in key…” replace “Type genus: *Microhoria* Chevrolat, 1877” with “Type genus: *Microhoria* Chevrolat, 1877 [placed on the Official List of Generic Names in Zoology (ICZN 2017)].”

**Family Aderidae Csiki, 1909**

Page 449. In the entry “Xylophilidae Shuckard, 1839b: 47…” replace “Type genus: *Xylophilus* Latreille, 1825” with “Type genus: *Xylophilus* Latreille, 1829”.

Page 449. Replace “Hylophilidae Pic, 1900: 754” with “Hylophilidae Streubel, 1846: 961”.

**Family Scraptiidae Gistel, 1848**

Page 450. Below the entry “Scraptiaeidae Gistel, 1848: [11]…” under the valid name “**Tribe Scraptiini Gistel, 1848**” add:

“Trotommideini Pic, 1903: 76 [stem: *Trotommide*-]. Type genus: *Trotommidea* Reitter, 1883.”

**Superfamily Tenebrionoidea Latreille, 1802**

Page 451. Insert “**Tenebrionoidea *incertae sedis***” above the valid name “**Subfamily Lagrioidinae Abdullah and Abdullah, 1968**”. Note. The unassigned family-group taxa Lagrioidinae Abdullah and Abdullah, 1968, Afreminae Levey, 1985, and Ischaliinae Blair, 1920 belong to Tenebrionoidea *incertae sedis* (Lawrence et al. 2010c).

**Family Cerambycidae Latreille, 1802**

Page 457. Replace the entry “*Ancistrotides Lacordaire, 1868: 81…” with:

“Ancistrotides Lacordaire, 1868: 81 [stem: *Ancistrot*-]. Type genus: *Ancistrotus* Audinet-Serville, 1832. Comment: original vernacular name available (Art. 11.7.2): first used in latinized form by Lameere (1919: 90, as Ancistrotini), generally accepted as in Cerda (1986: 30, as Ancistrotini).”

Page 457. Delete the entry “Ancistrotini Lameere, 1919: 90…”

Page 460. In the entries “*Cyrtognathites Blanchard, 1845b: 138...” and “Cyrthognathitae J. Thomson, 1861: 328…” replace “*Cyrtognathus* Faldermann, 1835” with “*Cyrtognathus* Dejean, 1835.” Note. For priority of author’s name, see Bousquet and Bouchard (2013: 79–80).

Page 462. Below the entry “*Grammoptérates Mulsant, 1863b: 569...” add:

“Grammopterini Della Beffa, 1915: 42 [stem: *Grammopter*-]. Type genus: *Grammopterus* Audinet-Serville, 1835.”

Page 463. Below the entry “Toxoti J. L. LeConte and Horn, 1883: 313...” add:

“Acmaeopsini Della Beffa, 1915: 42 [stem: *Acmaeop*-]. Type genus: *Acmaeops* J. L. LeConte, 1850. Comment: incorrect original stem formation, not in prevailing usage.”

Page 464. In the entry “*Criocéphalites Fairmaire, 1864: 125…” replace “Type genus: *Criocephalus* Mulsant, 1839” with “Type genus: *Criocephalum* Dejean, 1835”.

Page 464. Replace the entry “Criocephalinae Sharp, 1905: 147…” with:

“Criocephalinae Perrier, 1893: 1262 [stem: *Criocephal*-]. Type genus: *Criocephalum* Dejean, 1835 [syn. of *Arhopalus* Audinet-Serville, 1834].”

Page 467. Replace the valid name “**Tribe Auxesini Lepesme and Breuning, 1952**” with “**Tribe Auxesini Lacordaire, 1872**”.

Page 467. Replace the entry “*Auxésides Lacordaire, 1872: 463…” with:

“Auxésides Lacordaire, 1872: 463 [stem: *Auxes*-]. Type genus: *Auxesis* J. Tomson, 1858. Comment: original vernacular name available (Art. 11.7.2): first used in latinized form and generally accepted as in Gahan (1902: 278, as Auxesinae); current spelling maintained (Art. 29.5): incorrect original stem formation in prevailing usage (should be *Auxese*-).”

Page 467. Delete the entry “Auxesina Lepesme and Breuning, 1952: 140…”

Page 467. Replace the valid name “**Tribe Brachypteromatini Sama, 2008**” with “**Tribe Brachypteromini Sama, 2008**”.

Page 467. In the entry “Brachypteromini Sama, 2008: 229...” replace the stem with “*Brachypterom*-” and replace the “Comment” section with “Comment: incorrect original stem formation maintained under Art. 29.4 (should be *Brachypteromat*-).”

Page 473. Replace the valid name “**Tribe Hexoplini Martins, 2006**” with “**Tribe Hexoplonini Martins, 2006**”.

Page 473. In the entry “Hexoplonini Martins, 2006: 22...” replace the stem with “*Hexoplon*-” and replace the “Comment” section with “Comment: incorrect original stem formation maintained under Art. 29.4 (should be *Hexopl*-).”

Page 474. Replace the valid name “**Tribe Hylotrupini Zagajkevich, 1991**” with “**Tribe Hylotrupini Rose, 1983**”.

Page 474. Replace “Hylotrupini Zagajkevich, 1991: 67” with “Hylotrupini Rose, 1983: 48”.

Page 474. Replace the valid name “**Tribe Ibidionini Thomson, 1861**” with “**Tribe Tropidini Martins and Galileo, 2007**” and move all associated records above “**Tribe Tropocalymmatini Lacordaire, 1868**” on page 485 in order to maintain the alphabetical order of valid tribes.

Page 474. Replace “Ibidionitae J. Thomson, 1861: 199...” under the valid name “**Tribe Tropidini Martins and Galileo, 2007**” with:

“Tropidina Martins and Galileo, 2007: 7 [stem: *Tropid*-]. Type genus: *Tropidion* J. Thomson, 1867.”

Page 474. Replace the valid name “**Subtribe Ibidionina Thomson, 1861**” with “**Subtribe Neoibidionina Monné, 2012**”.

Page 474. Replace the entry “Ibidionitae J. Thomson, 1861: 199...” under the valid name “**Subtribe Neoibidionina Monné, 2012**” with:

“Ibidionitae J. Thomson, 1861: 199 [stem: *Ibidion*-]. Type genus: *Ibidion* Audinet-Serville, 1834 [preoccupied genus name, not *Ibidion* Gory, 1833; syn. of *Neoibidion* Monné, 2012]. Comment: incorrect stem formation (should be *Ibidi*-); permanently invalid (Art. 39): based on preoccupied type genus.”

Page 474. Below the entry “*Sydacini Martins, 2003a: 204...” add:

“Neoibidionini Monné, 2012: 35 [stem: *Neoibidion*-]. Type genus: *Neoibidion* Monné, 2012. Comment: incorrect original stem formation maintained under Art. 29.4 (should be *Neoibidi*-).” Note. Monné (2012) proposed the replacement name Neoibidionini for the tribe Ibidionini J. Thomson because of the homonymy of the type genus *Ibidion* Audinet-Serville. Howewer, the replacement name could apply only to the rank of subtribe since there are two older available names which could be used as valid for the tribe, Compsina Martins and Galileo, 2007 and Tropidina Martins and Galileo, 2007. We here select Tropidini Martins and Galileo, 2007 as the valid name for the tribe.

Page 479. Replace “Leptideina Reitter, 1913a: 24” with “Leptideinae Perrier, 1893: 1262”.

Page 482. In the entry “*Ptérosténides Lacordaire, 1868: 410…” replace “Type genus: *Pterostenus* Laporte, 1840” with “Type genus: *Pterostenus* Dejean, 1835” Note. See Bousquet and Bouchard (2013: 91).

Page 485. Above the valid name “**Tribe Tropocalymmatini Lacordaire, 1868**” add:

“**Tribe Trigonarthrini Villiers, 1984.**

Trigonarthrini Villiers, 1984: 1 [stem: *Trigonarthr*-]. Type genus: *Trigonarthron* Boppe, 1912.”

Page 486. In the entry “Trypanidiitae J. Thomson, 1860a: 7” replace “Type genus: *Trypanidius* Blanchard, 1846” with “Type genus: *Trypanidius* Blanchard, 1842” Note. The generic name *Trypanidius* is usually attributed to Blanchard (1846 [should be 1847]: 209) in the literature but it was made available first by Blanchard (1842: pl. 22, fig. 6) when he illustrated the species *Trypanidius andicola*.

Page 486. In the entry “Liopi J. L. LeConte, 1873: 338…” add at the end of the “Comment” section “; the family-group name Leiopidae [should be Leiopodidae] Lang, 1970 (type genus *Leiopus* Beddard, 1886) is available in Crustacea though permanently invalid (based on preoccupied type genus).”

Page 486. Below the entry “Liopi J. L. LeConte, 1873: 338…” add:

“Aedilinae Perrier, 1893: 1263 [stem: *Aedil*-]. Type genus: *Aedilis* Audinet-Serville, 1835.”

Page 488. In the entry “Ancitini Aurivillius, 1917: 28…” replace “Type genus: *Ancita* J. Thomson, 1864” with “Type genus: *Ancita* J. Thomson, 1864 [syn. of *Hebecerus* Dejean, 1835].” See additional notes for name Hebesecinae Pascoe, 1871 below.

Page 491. Move the entry “Hebesecinae Pascoe, 1871: 277…” to page 488 above the entry “Ancitini Aurivillius, 1917: 28…” Note. The type genus *Hebesecis* Pascoe, 1865 is a replacement name for *Hebecerus* Dejean, 1835, which used to be attributed to “Thomson, 1864” and treated as a junior homonym of *Hebecerus* Kolenati, 1845 [Hemiptera] until recently (see Bousquet and Bouchard 2013: 83). *Hebecerus* Dejean, 1835 is a senior synonym of *Ancita* Thomson, 1864 and Hebesecini Pascoe, 1871 is a senior synonym of Ancitini Aurivillius, 1917. An application to the Commission is necessary to conserve usage of *Ancita* Thomson, 1864 and Ancitini Aurivillius, 1917, which have been treated as valid in recent literature (e.g., Ślipiński and Escalona 2013: 93).

Page 492. In the entry “Crinotarsides Lacordaire, 1872: 475…” replace “Type genus: *Crinotarsus* Blanchard, 1853” with “Type genus: *Crinotarsus* Blanchard, 1846” Note. The generic name *Crinotarsus* is usually attributed to Blanchard (1853: 275) in the literature but it was made available first by Blanchard (1846: pl. 16, fig. 10) when he illustrated the species *Crinotarsus plagiatus*.

Page 492. Replace the entry “Apodasyides Lacordaire, 1872: 623 …” with:

“Apodasyides Lacordaire, 1872: 623 [stem: *Apodasy*-]. Type genus: *Apodasya* Pascoe, 1863 [the senior objective synonym *Chaetosoma* Chevrolat, 1843 was recently suppressed for both the Principle of Priority and the Principle of Homonymy, placed on the Official Index of Rejected and Invalid Generic Names in Zoology and *Apodasya* Pascoe, 1863 placed on the Official List of Generic Names in Zoology (ICZN 2011b)]. Comment: original vernacular name available (Art. 11.7.2): first used in latinized form by Kolbe (1897: 321, as Apodasinae [incorrect stem formation]), generally accepted as in Aurivillius (1922a: 305, as Apodasyini).”

Page 492. Below the entry “Eupogonii J. L. LeConte, 1873: 342…” add:

“Anaesthetinae Perrier, 1893: 1263 [stem: *Anaesthet*-]. Type genus: *Anaesthetis* Dejean, 1835.”

Page 494. Above the valid name “**Eupromerini Galileo and Martins, 1995**” add:

“**Tribe Eunidiini Téocchi, Sudre and Jiroux, 2010.**

Eunidiini Téocchi, Sudre and Jiroux, 2010: 13 [stem: *Eunidi*-]. Type genus: *Eunidia* Erichson, 1843.”

Page 500. At the end of the entry “Niphoninae Pascoe, 1864: 56…” add “Comment: the family-group name Niphonidae Jordan, 1923 (type genus *Niphon* Cuvier et Valenciennes, 1828) is available in Pisces but has been incorrectly formed in the literature, the correct stem based on *Niphon* is *Niphont*- (Alonso-Zarazaga pers. comm. December 2019).”

Page 503. In the entry “Ichthyosomitae J. Thomson, 1864: 33…” replace “[stem: *Ichthyosom*-]. Type genus: *Ichthyosomus* Boisduval, 1835” with “[stem: *Ichthyosomat*-]. Type genus: *Ichthyosoma* Boisduval, 1835. Comment: incorrect original stem formation, not in prevailing usage.”

**Family Chrysomelidae Latreille, 1802**

Page 506. In the entry “Amblycerinae Bridwell, 1932: 103, in key…” under the valid name “**Subtribe Amblycerina Bridwell, 1932**” replace “although this is the oldest name for the tribe” in the “Comment” section with “although this is not the oldest name for the tribe”.

Page 509. Below the entry “Haemoniini Chen, 1941: 8…” add:

“Macropleini Lopatin, 1977: 53 [stem: *Macrople*-]. Type genus: *Macroplea* Samouelle, 1819”.

Page 510. Replace “Aprioidini Weise, 1911: 41” with “Aproidini Weise, 1911: 41”.

Page 513. Replace the valid name “**Tribe Chalepini Weise, 1910**” with “**Tribe Chalepini Weise, 1910 *nomen protectum***”.

Page 513. Above the entry “Chalepini Weise, 1910: 69…” add the entries:

“Stenopodiides Horn, 1883: 290 [stem: *Stenopodi*-]. Type genus: *Stenopodius* Horn, 1883. Comment: this and the following family-group name have precedence over Chalepini Weise, 1910; as far as we know family-group names based on *Stenopodius* Horn and *Microrhopala* Chevrolat have not been used as valid after 1899 and we found 25 references (Appendix 1), using Chalepini as valid, in the preceding 50 years published by at least 10 authors and encompassing a span of not less than 10 years; therefore Stenopodiides Horn, 1883 and Microrhopalides Horn, 1883 are *nomina oblita* and Chalepini Weise, 1910 a *nomen protectum* following Art. 23.9.2 (ICZN 1999).”

“Microrhopalides Horn, 1883: 290 [stem: *Microrhopal*-]. Type genus: *Microrhopala* Chevrolat, 1836.”

Page 513. In the entry “Chalepini Weise, 1910: 69…” replace “Comment: Chalepini Weise, 1910 is a junior homonym of Chalepidae H. C. C. Burmeister, 1847…” with “Comment: *nomen protectum* (see Appendix 1); Chalepini Weise, 1910 is a junior homonym of Chalepidae Streubel, 1846…”

Page 515. In the entry “Hispoleptites Chapuis, 1875: 283…” replace “Type genus: *Hispopleptis* Baly, 1864” with “Type genus: *Hispoleptis* Baly, 1864”.

Page 516. Replace the entry “*Physonotitae Spaeth, 1942: 32…” with:

“Physonotitae Spaeth, 1942: 32 [stem: *Physonot*-]. Type genus: *Physonota* Boheman, 1854. Comment: name proposed after 1930 without description or bibliographic reference to such a description (Art. 13.1), however available because it was used as valid before 2000 as in Balsbaugh and Hays (1972: 191) and was not rejected by an author who, between 1961 and 1999, applied Article 13 of the then current edition of the Code (see Art. 13.2.1).”

Page 516. Replace the valid name “**Tribe Nothosacanthini Gressitt, 1952 (1929)**” with “**Tribe Notosacanthini Gressitt, 1952 (1929)**”

Page 517. Replace the entry “Oncocéphalites Chapuis, 1875: 308…” with:

“Oncocéphalites Chapuis, 1875: 308 [stem: *Oncocephal*-]. Type genus: *Oncocephala* Agassiz, 1846. Comment: original vernacular name available (Art. 11.7.2): first used in latinized form by Weise (1911: 50, as Oncocephalini), generally accepted as in Świętojańska et al. (2006: 49, as Oncocephalini).” Note. *Oncocephala* Agassiz, 1846 is an unjustified emendation of *Onchocephala* Guérin-Méneville, 1844, which is a junior homonym of *Onchocephala* de Blainville, 1828 (Annelides).

Page 518. In the entry “Promecothécites Chapuis, 1875: 300…” replace “Type genus: *Promecotheca* Chevrolat, 1847” with “Type genus: *Promecotheca* Guérin-Méneville, 1840” Note. This genus name is credited by most authors to Blanchard (1853: 312) or Chevrolat (1847b: 482); however, it was first made available by Guérin-Méneville (1840: 334).

Page 518. In the entry “Prosopodontini Weise, 1910: 69…” replace “Type genus: *Prosopodonta* Baly, 1885” with “Type genus: *Prosopodonta* Baly, 1858”.

Page 519. Below the entry “*Octotomites Chapuis, 1875: 310…” add:

“Octotomides Horn, 1883: 290 [stem: *Octotom*-]. Type genus: *Octotoma* Dejean, 1836. Comment: this family-group name has precedence over Uroplatini Weise, 1910; Octotomidae was used as valid after 1899 (e.g., Ienistea 1986: 31) and therefore Octotomini Horn, 1883 cannot be treated as a *nomen oblitum* (Art. 23.9.1.1); an application to the Commission is necessary to conserve usage of the well-established name Uroplatini Weise, 1910.”

Page 520. Above the entry “*Australicites Chapuis, 1874: 428…” add:

“Colaphidae Siegel, 1866: 102 [stem: *Colaph*-]. Type genus: *Colaphus* L. Redtenbacher, 1845 [preoccupied genus name, not *Colaphus* Dejean, 1836 [Coleoptera: Chrysomelidae]; syn. of *Colaphellus* Weise, 1916]. Comment: permanently invalid (Art. 39): based on preoccupied type genus.”

Page 522. At the end of the entry “Gastrophysina Kippenberg, 2010a: 68…” add: “Comment: the senior homonym Gastrophysini Harting, 1864 (type genus *Gastrophysus* Müller, 1843) is available in Pisces; this case is to be referred to the Commission to remove the homonymy (Art. 55.3.1).”

Page 522. Delete the entry “Hesperidae Swainson, 1840: 310…”

Page 524. In the entry “Oxygonites Chapuis, 1875: 43…” at the end of the comment for the type genus replace “…Chevrolat (1847: 368)].” with “…Chevrolat (1847: 368); syn. of *Platiprosopus* Chevrolat, 1834].”

Page 527. In the entry “Chorini Weise, 1923: 124…” add at the end of the “Comment” section “; the family group name Chorininae Dana, 1851 (type genus *Chorinus* Latreille, 1825) is available in Crustacea but currently considered a synonym of Pisinae Dana, 1851; this case is to be referred to the Commission to remove the homonymy (Art. 55.3.1).”

Page 530. Replace “Androlyperini Leng, 1920: 298” with “Androlyperites G. H. Horn, 1893: 59” and move the entry above “Phyllecthrites G. H. Horn, 1893: 60, in key…”

Page 533. At the end of the entry “Monachites Chapuis, 1874: 172…” replace “Monachinae Trouessart, 1897 (type genus *Monachus* Fleming, 1822) is available in Mammalia.” with “Monachinae Trouessart, 1897 (type genus *Monachus* Fleming, 1822) is available in Mammalia but a junior homonym of the Coleoptera family-group name based on *Monachus* Chevrolat, 1836; an application to the Commission is needed to conserve usage of the well established name in Mammalia.”

Page 533. Below the entry “Monachulini Leng, 1920: 290…” add:

“Lexiphanini Wilcox, 1954: 379, in key [stem: *Lexiphan*-]. Type genus: *Lexiphanes* Gistel, 1848.”

Page 534. In the entry “Eumolpidae Hope, 1840a: 162…” replace “Type genus: *Eumolpus* Kugelann, 1798 [an application to suppress *Eumolpus* Kugelann, 1798 and conserve *Eumolpus* Weber, 1801 was recently submitted to the Commission by Moseyko et al. (2010)].” with “Type genus: *Eumolpus* Weber, 1801 [placed on the Official List of Generic Names in Zoology (ICZN 2012b)]. Comment: it appears that Hope (1840) intended to create the family-group name Eumolpidae based on *Eumolpus* Weber, 1801, not *Eumolpus* Kugelann, 1798 (M. Alonso-Zarazaga, pers. comm. 2016); since *Eumolpus* Kugelann, 1798 (as “*Eumolpus* Illiger, 1798”) has been suppressed for the purposes of both the Principle of Priority and the Principle of Homonymy and placed on the Official Index of Rejected and Invalid Generic Names in Zoology in Opinion 2298 (ICZN 2012b), *Eumolpus* Weber, 1801 is not a junior homonym and the name Eumolpidae Hope, 1840 can be retained as valid.”

Page 534. In the entry “Bromiinae Baly, 1865: 438…” replace “Type genus: *Bromius* Chevrolat, 1836 [an application to conserve *Bromius* Chevrolat, 1836, threatened by the older name *Eumolpus* Kugelann, 1798, was recently submitted to the Commission by Moseyko et al. (2010)].” with “Type genus: *Bromius* Chevrolat, 1836 [placed on the Official List of Generic Names in Zoology (ICZN 2012b)]”.

Page 536. Replace the entry “Eumolpidae Hope, 1840a: 162…” with:

“Eumolpidae Hope, 1840a: 162 [stem: *Eumolp*-]. Type genus: *Eumolpus* Weber, 1801 [placed on the Official List of Generic Names in Zoology (ICZN 2012b)]. Comment: First Reviser (Eumolpini Hope, 1840 vs Colaspidini Hope, 1840) not determined, current usage maintained.” Note. See additional comments in suggested correction for the entry “Eumolpidae Hope, 1840a: 162...” on page 534 above.

Page 536. Replace the entry “Edusites Chapuis, 1874: 306…” with:

“Edusites Chapuis, 1874: 306 [stem: *Edus*-]. Type genus: *Edusa* Chevrolat, 1836. Comment: original vernacular name available (Art. 11.7.2): first used in latinized form and generally accepted as in Lefèvre (1885: 111, as Edusitae).” Note. As mentioned by Bousquet and Bouchard (2013: 111) the type genus *Edusa* was first made available in Coleoptera by Chevrolat (1836), as opposed to Chapuis (1874) as previously understood. This removes the homonymy problem with other genera named *Edusa* after 1836 and therefore the family-group name based on *Edusa* Chevrolat, 1836 can be used as valid in the future.

**Family Nemonychidae Bedel, 1882**

Page 541. Replace the valid name “†**Tribe Kuschelomacrini Riedel, 2010**” with “†**Tribe Kuschelomacerini Riedel, 2010**”.

Page 541. In the entry “Kuschelomacerini Riedel, 2010: 31...” replace the stem with “*Kuschelomacer*-” and replace the “Comment” section with “Comment: incorrect original stem formation maintained under Art. 29.4 (should be “*Kuschelomacr*-”).”

Page 544. Above the valid name “†**Subfamily Cretonemonychinae Gratshev and Legalov, 2009**” add the following entries:

“†**Tribe Metrioxenoidini Legalov, 2009**

Metrioxenoidinae Legalov, 2009c: 288 [stem: *Metrioxenoid*-]. Type genus: *Metrioxenoides* Gratshev et al., 1998.”

“†**Tribe Medmetrioxenoidesini Legalov, 2010**

Medmetrioxenoidesini Legalov, 2010: 471 [stem: *Medmetrioxenoides*-]. Type genus: *Medmetrioxenoides* Gratshev and Legalov, 2009. Comment: incorrect original stem formation maintained under Art. 29.4 (should be “*Medmetrioxenoid*-”).”

†**Tribe Megametrioxenoidesini Legalov, 2010**

Megametrioxenoidesini Legalov, 2010: 471 [stem: *Megametrioxenoides*-]. Type genus: *Megametrioxenoides* Gratshev and Legalov, 2009. Comment: incorrect original stem formation maintained under Art. 29.4 (should be “*Megametrioxenoid*-”).”

**Family Anthribidae Billberg, 1820**

Page 546. At the end of the entry “Meconemini Pierce, 1930: 4, in key…” add: “Comment: the junior homonym Meconemini Burmeister, 1838 (type genus *Meconema* Audinet-Serville, 1831) is available in Orthoptera; this case is to be referred to the Commission to remove the homonymy (Art. 55.3.1).”

**Family Caridae Thompson, 1992**

Page 553. Replace the valid name “**Tribe Carodini Legalov, 2009**” with “**Tribe Carodesini Legalov, 2009**”.

Page 553. In the entry “Carodesina Legalov, 2009: 126...” replace the stem with “*Carodes*-” and replace the “Comment” section with “Comment: incorrect original stem formation maintained under Art. 29.4 (should be *Carod*-).”

**Family Attelabidae Billberg, 1820**

Page 555. In the entry “Archeuopsina Legalov, 2003: 359...” replace the stem with “*Archeuops*-” and replace the “Comment” section with “Comment: proposed as a subtribe of Euopini; incorrect original stem formation maintained under Art. 29.4 (should be *Archeuop*-).”

Page 556. In the entry “Suniopsina Legalov, 2003: 364...” replace the stem with “*Suniops*-” and replace the “Comment” section with “Comment: proposed as a subtribe of Euopini; incorrect original stem formation maintained under Art. 29.4 (should be *Suniop*-).”

Page 556. In the entry “Synaptopsina Legalov, 2003: 368...” replace the stem with “*Synaptops*-” and replace the “Comment” section with “Comment: proposed as a subtribe of Euopini; incorrect original stem formation maintained under Art. 29.4 (should be *Synaptop*-).”

Page 556. In the entry “Ljudmilinina Legalov, 2007: 219...” replace the stem with “*Ljudmilin*-” and replace the “Comment” section with “proposed as a subtribe of Euopini; incorrect stem formation maintained under Art. 29.4 (should be *Ljudmilini*-).”

Page 556. In the entry “Parasynaptopsisina Legalov, 2007: 227...” replace the stem with “*Parasynaptopsis*-” and replace the “Comment” section with “proposed as a subtribe of Euopini; incorrect stem formation maintained under Art. 29.4 (should be *Parasynaptopse*-).”

Page 556. In the entry “Riedelinina Legalov, 2007: 218...” replace the stem with “*Riedelin*-” and replace the “Comment” section with “proposed as a subtribe of Euopini; incorrect stem formation maintained under Art. 29.4 (should be *Riedelini*-).”

Page 556. In the entry “Sawadaeuopsina Legalov, 2007: 241...” replace the stem with “*Sawadaeuops*-” and replace the “Comment” section with “Comment: proposed as a subtribe of Euopini; incorrect original stem formation maintained under Art. 29.4 (should be *Sawadaeuop*-).”

Page 558. Replace the valid name “**Subtribe Auletobiina Legalov, 2001**” with “**Subtribe Auletobiina Voss, 1930**”.

Page 558. Above the entry “Auletobiina Legalov, 2001: 37…” add:

“Auletobini Voss, 1930: 60 [stem: *Auletobi*-]. Type genus: *Auletobius* Desbrochers des Loges, 1869. Comment: incorrect original stem formation, not in prevailing usage.”

Page 558. At the end of the entry “Auletobiina Legalov, 2001: 37…” add “Comment: family-group name proposed as new without reference to Auletobiina Voss, 1930.”

Page 559. Move the entry “*Rhynchitallini Voss, 1960: 415...” to before the entry “Rhynchitallina Legalov, 2003: 226...” on page 560.

Page 559. Delete the entry “Proteugnamptini Legalov, 2003: 80...”

Page 560. In the entry “†Sanyrevilleina Legalov, 2003: 85…” replace “[stem: *Sanyreville*-]. Type genus: *Sanyrevilleus* Gratshev and Zherikhin, 2000. Comment: proposed as a subtribe of Auletanini; raised to tribal level by Legalov (2007).” with “[stem: *Sayreville*-]. Type genus: *Sayrevilleus* Gratshev and Zherikhin, 2000. Comment: proposed as a subtribe of Auletanini; raised to tribal level by Legalov (2007); incorrect original stem formation, not in prevailing usage.”

**Family Brentidae Billberg, 1820**

Page 569. Replace the valid name “**Tribe Noterapiini Kissinger, 2004**” with “**Tribe Noterapionini Kissinger, 2004**”.

Page 569. In the entry “Noterapionini Kissinger, 2004: 243...” replace the stem with “*Noterapion*-” and replace the “Comment” section with “Comment: incorrect original stem formation maintained under Art. 29.4 (should be *Noterapi*-).”

**Family Curculionidae Latreille, 1802**

Page 579. Replace “Brachonychina Voss, 1944: 38” with “Brachonychinae Joy, 1932: 159, in key”.

Page 579. In the entry “Camarotides Schönherr, 1833: 4…” replace “Type genus: *Camarotus* Germar, 1817” with “Type genus: *Camarotus* Germar, 1833”

Page 582. Below the entry “Otidocéphalides Lacordaire, 1863: 568…” add:

“Myrmecinae Tanner, 1966: 6, in key [stem: *Myrmec*-]. Type genus: *Myrmex* Sturm, 1826.”

Page 585. Replace “Orthocaetina Morimoto, 1962a: 56, in key” with “Orthochaetinae Joy, 1932: 159 (in key), 208” and remove the “Comment” section.

Page 591. Below the entry “Poophagidae Schultze, 1902: 226…” add:

“Tapinotinae Joy, 1932: 160, in key [stem: *Tapinot*-]. Type genus: *Tapinotus* Schönherr, 1826.” Note. See Colonnelli (2013: 58) for a discussion regarding the correct spelling of the type genus name.

Page 594. Below the entry “*Sympiézopides Lacordaire, 1865: 166…” add:

“Sympiezopinorum Faust, 1886b: 367. [stem: *Sympiezopod*-]. Type genus: *Sympiezopus* Schönherr, 1837. Comment: Sympiezopinorum is the genitive of Sympiezopini (Alonso-Zarazaga pers. comm. December 2019); incorrect original stem formation, not in prevailing usage.”

Pages 599–600. Below the entry for “Tylodides Lacordaire, 1865: 90” add:

“Acallinae Joy, 1932: 160, in key [stem: *Acall*-]. Type genus: *Acalles* Schönherr, 1825.”

Page 600. In the entry “Psépholacides Lacordaire, 1865: 72…” replace “Type genus: *Psepholax* Lacordaire, 1865” with “Type genus: *Psepholax* White, 1843”.

Page 602. In the entry “Hipporhinides Lacordaire, 1863: 323…” replace “Type genus: *Hipporhinus* Schönherr, 1823” with “Type genus: *Hipporhis* Billberg, 1820 [as *Hipporhinus*, unjustified emendation of the type genus by Schönherr, 1823, not in prevailing usage; syn. of *Bronchus* Germar, 1817]”.

Page 604. In the entry “Strophosomidae Gistel, 1848: [8]…” replace “Type genus: *Strophosomum* Gistel, 1856 [syn. of *Strophosoma* Billberg, 1820]” with “Type genus: *Strophosoma* Billberg, 1820.”

Page 610. Replace the valid name “**Tribe Mesostylini Reitter, 1913**” with “**Tribe Mesostyloidini Bouchard & Bousquet, *nomen novum***”

Page 610. Replace the entry “Mesostylini Reitter, 1913b: 8...” with:

“Mesostylini Reitter, 1913b: 8 [stem: *Mesostyl*-]. Type genus: *Mesostylus* Faust, 1894 [preoccupied genus name, not *Mesostylus* Bronn and Roemer, 1852 [Crustacea]; syn. of *Mesostyloides* Bouchard and Bousquet, ***nomen*** [***novum***]. Comment: permanently invalid (Art. 39); based on preoccupied type genus.” Note. Although the crustacean name *Mesostylus* Bronn and Roemer, 1852 was treated as a nomen oblitum by Karasawa (2003: 181), this genus was used as valid more recently by Schweitzer and Feldmann (2012: 17).

Page 610. Below the entry “Mesostylini Reitter, 1913b: 8...” add:

“Mesostyloidini Bouchard and Bousquet, ***nomen novum*** for Mesostylini Reitter, 1913 [stem: *Mesostyloid*-]. Type genus: *Mesostyloides* Bouchard and Bousquet, ***nomen novum*** for *Mesostylus* Faust, 1894.”

Page 612. Replace the entry “Loborhynchides Schönherr, 1823: column 1144…” with:

“Loborhynchides Schönherr, 1823: column 1144 [stem: *Loborhynch*-]. Type genus: *Loborhynchus* Dejean, 1821 [placed on the Official Index of Rejected and Invalid Generic Names in Zoology (ICZN 1972, ICZN 2012c)]. Comment: Loborhynchinae Schönherr, 1823 placed on the Official Index of Rejected and Invalid Family-Group Names in Zoology (ICZN 1972).”

Page 613. Replace the entry “Otiorhynchides Schönherr, 1826: 203…” with:

“Otiorhynchides Schönherr, 1826: 203 [stem: *Otiorhynch*-]. Type genus: *Otiorhynchus* Germar, 1822 [placed on the Official List of Generic Names in Zoology (ICZN 1972, ICZN 2012c)]. Comment: name placed on the Official List of Family-Group Names in Zoology (ICZN 1972, as Otiorhynchinae Schönherr, 1826).” Note. The entry on the Official List of Generic Names in Zoology for the genus *Otiorhynchus* was emended from “*Otiorhynchus* Germar, 1824, *Insectorum species novae aut minus cognitae, descriptionibus illustratae*, vol. 1.” to “*Otiorhynchus* Germar, 1822, *Fauna Insectorum Europae*, 7: no. 12” (ICZN 2012c).

Page 615. In the entry “Sitonisidae Gistel, 1848: [8]…” replace “Type genus: *Sitones* Schönherr, 1840 [syn. of *Sitona* Germar, 1817]” with “Type species: *Sitona* Germar, 1817 [as *Sitones*, unjustified emendation of the type genus by Schönherr (1840b: 253), not in prevailing usage]”.

Page 616. Below the entry for “Pandeleteini Pierce, 1913: 399…” add:

“Cycloderini Hoffmann, 1950: 417 [stem: *Cycloder*-]. Type genus: *Cycloderes* C. R. Sahlberg, 1823.”

Pages 617. In the entry “Tropiphoridae Marseul, 1863: 220…” delete the “Comment” section. Note. The date of 15 June 1863 on the first page of Marseul’s *Catalogue* does not pertain to the date of publication but to the date of the preliminaries. The work was published in November 1863 (Marseul 1864: xxv, as “Nov. 1843” [sic]). The new date of publication found means that the family-group names Strangaliodini Lacordaire, 1863, Byrsopagini Lacordaire, 1863, Pantopoeini Lacordaire, 1863 and Synaptonychini Lacordaire, 1863 have precedence over Marseul’s Tropiphorini since Lacordaire’s book was issued by 1 August 1863 (Bousquet 2016: 314). An application to the Commission is necessary to preserve usage of Tropiphorini Marseul, 1863 in Curculionidae: Entiminae, or alternatively, one or more of the available family-group names proposed by Lacordaire (1863) could be used as valid (e.g., Alonso-Zarazaga et al. 2017: 543).

Page 618. In the entry “Stenocorynini McKeown, 1939: 408…” replace “Type genus: *Stenocorynus* Schönherr, 1842” with “Type genus: *Stenocorynus* Schönherr, 1823”

Page 619. Replace the valid name “**Tribe Hyperini Marseul, 1863 (1848)**” with “**Tribe Hyperini Lacordaire, 1863 (1848)**”.

Page 619. Replace the entry “Hyperidae Marseul, 1863: 224…” with:

“Hypérides Lacordaire, 1863: 395 [stem: *Hyper*-]. Type genus *Hypera* Germar, 1817. Comment: original vernacular name available (Art. 11.7.2): first used in latinized form by Marseul (1863: 224, as Hyperidae), generally accepted as in Morrone and Roig Juñent (1995: 12, as Hyperinae).” Note. As mentioned above the date of 15 June 1863 on the first page of Marseul’s *Catalogue* does not pertain to the date of publication but to the date of the preliminaries. The work was published in November 1863 and so Lacordaire’s name was proposed earlier [by 10 August 1863] and takes priority.

Page 619. In the entry “Macrotarrhusina Legalov, 2007: 401...” replace the stem with “*Macrotarrhus*-” and replace the “Comment” section with “Comment: incorrect original stem formation maintained under Art. 29.4 (should be *Macrotarrh*-).”

Page 623. In the entry “Brachyceropseinae Aurivillius, 1926b: 2…” replace “Type genus: *Brachyceropsis* Aurivillius, 1926” with “Type genus: *Brachyceropsis* Aurivillius, 1886”.

Page 628. Replace the entry “Plinthides Lacordaire, 1863: 359…” with:

“Plinthides Lacordaire, 1863: 359 [stem: *Plinth*-]. Type genus: *Plinthus* Germar, 1817 [placed on the Official List of Generic Names in Zoology (ICZN 2012d)]. Comment: name placed on the Official List of Family-Group Names in Zoology (ICZN 2012d, as Plinthini Lacordaire, 1863).”

Page 628. Below the entry “Minyopidae Marseul, 1863: 221…” add:

“**Subtribe Sthereina Hatch, 1971**

Sthereini Hatch, 1971: 309 [stem: *Sthere*-]. Type genus: *Sthereus* Motschulsky, 1845.”

Page 635. In the entry “*Sueinae Murayama, 1959: 26...” remove the asterisk (*) and replace the “Comment” section with “Comment: name proposed after 1930 without description or bibliographic reference to such a description (Art. 13.1), however available because it was used as valid before 2000 as in Murayama (1963: 4, as Sueinae) and was not rejected by an author who, between 1961 and 1999, applied Article 13 of the then current edition of the Code (Art. 13.2.1).”

Page 635. Delete the entry “Sueinae Murayama, 1963: 4…”

Page 637. Replace the entry “*Eccoptopterina Browne, 1961: 49…” with:

“Eccoptopterini Kalshoven, 1959: 166 [stem: *Eccoptopter*-]. Type genus: *Eccoptopterus* Motschulsky, 1863. Comment: name proposed after 1930 without description or bibliographic reference to such a description (Art. 13.1), however available because it was used as valid before 2000 as in Browne (1961: 49, as Eccoptopterina) and was not rejected by an author who, between 1961 and 1999, applied Article 13 of the then current edition of the Code (Art. 13.2.1).”

**Coleoptera *incertae sedis***

Page 639. Insert an asterisk (*) in front of “Serratopalpidae Gistel, 1856a: 384” since the family-group name is not available.

Page 639. Delete the entry “Homoeoplastidae Gistel, 1856a: 360…” since this family-group name is available (see above, under p. 354).

Page 639. Delete the entry “Plocasteidae Gistel, 1856a: 365…” since this family-group name is available (see above, under p. 232).

## References

**Delete the following references:**

Biström O, Nilsson AN, Wewalka G (1997)…

Hatch MH (1928)…

Latreille PA (1819)…

Régimbart M (1882)…

Winkler A (1925)…

Zimmermann A (1919)…

**Add the following references:**

Alonso-Zarazaga MA, Barrios H, Borovec R, Bouchard P, Caldara R, Colonnelli E, Gültekin L, Hlaváč P, Korotyaev B, Lyal CHC, Machado A, Meregalli M, Pierotti H, Ren L, Sánchez-Ruiz M, Sforzi A, Silfverberg H, Skuhrovec J, Trýzna M, Velázquez de Castro AJ, Yunakov NN (2017) Cooperative catalogue of Palaearctic Coleoptera Curculionoidea. Monografías electrónicas SEA (Sociedad Entomológica Aragonesa) 8: 1–729.

Alonso-Zarazaga MA, Krell F-T (2011) Change of authorship of *Aphodius* and *Oryctes* to Hellwig, 1798 (Insecta: Coleoptera: Scarabaeidae). Zootaxa 3060: 67–68. https://doi.org/10.11646/zootaxa.3060.1.5

Arnett RH (1984) The false blister beetles of Florida (Coleoptera: Oedemeridae). Entomology Circular No. 259, 4 pp.

Balsbaugh Jr EU, Hays KL (1972) The leaf beetles of Alabama (Coleoptera: Chrysomelidae). Agricultural Experiment Station, Auburn University, Bulletin 441, 223 pp.

Bates F (1872) Notes on Heteromera, and descriptions of new genera and species (no. 1). The Entomologist’s Monthly Magazine 9 [1872–73]: 97–99. https://doi.org/10.5962/bhl.part.4726

Bates F (1890) Heteromera. In: Scientifc results of the second Yarkand mission; based upon the collections and notes of the late Ferdinand Stoliczka, Ph.D. Coleoptera. Published by order of the government of India. Ofce of Superintendent of Government printing, Calcutta, 55–79. [+ 1 pl]

Baudi di Selve F (1875) Coleotteri Tenebrioniti delle collezioni italiane. Bullettino de la Società Entomologica Italiana 7: 137–165.

Beaulieu G (1919) Monographie des melasides du Canada. Le Naturaliste Canadien 46: 185–191.

Berthold AA (1827) Latreille’s Natürliche Familien des Thierreichs. Aus dem Französischen. Mit Anmerkungen und Zusätzen. Landes-Industrie-Comptoirs, Weimar, x + 606 pp. https://doi.org/10.5962/bhl.title.11652

Blanchard CÉ (1842) Insectes de l’Amérique méridionale. Recueillis par Alcide d’Orbigny et décrits par Emile Blanchard et Auguste Brullé [pls 14, 22] In: Voyage dans l’Amérique méridionale (le Brésil, la République orientale de l’Uruguay, la République Argentine, la Patagonie, la République du Chili, la République de Bolivie, la République du Pérou), exécuté pendant les années 1826, 1827, 1828, 1829, 1830, 1831, 1832 et 1833 par Alcide d’Orbigny. Tome sixième. 2.e Partie: Insectes. P. Bertrand, Paris [&] V. Levrault, Strasbourg, [4] + 222 pp. [+ 32 associated pls]

Blanchard CÉ (1846) Voyage au Pole Sud et dans l’Océanie sur les corvettes l’Astrolabe et la Zélée; exécuté par ordre du roi pendant les années 1837–1838–1839–1840. Zoologie Atlas. Paris. [pls 16–17]

Blanchard CÉ (1847) Insectes de l’Amérique méridionale. Recueillis par Alcide d’Orbigny et décrits par Emile Blanchard et Auguste Brullé [pp. 185–222] In: Voyage dans l’Amérique méridionale...Tome sixième. 2.e Partie: Insectes. P. Bertrand, Paris [&] V. Levrault, Strasbourg, [4] + 222 pp. [+ 32 associated pls].

Blanchard CÉ (1853) Voyage au Pole Sud et dans l’Océanie sur les corvettes l’Astrolabe et la Zélée; exécuté par ordre du Roi pendant les années 1837–1838–1839–1840, sous le commandement de M.J. Dumont-d’Urville, Capitaine de vaisseau; publié par ordre du gouvernement, sous la direction supérieure de M. Jacquinot, Capitaine de vaisseau, commandant de la Zélée. Zoologie par MM. Hombron et Jacquinot. Tome quatrième. Gide et J. Baudry, Paris, 422 pp.

Borchmann F (1915) Die Lagriinae (Unterfamilie der Lagriidae). Archiv für Naturgeschichte 81A (6): 46–186.

Bouchard P, Bousquet Y, Davies AE, Alonso-Zarazaga MA, Lawrence JF, Lyal CHC, Newton AF, Reid CAM, Schmitt M, Ślipiński SA, Smith ABT (2011) Family-group names in Coleoptera (Insecta). ZooKeys 88: 1–972. https://doi.org/10.3897/zookeys.88.807

Boucher S, Bai M, Wang B, Montreuil O (2017) Ceracyclini, tribe nov. of Passalidae Aulacocyclinae for *Cylindrocaulus* Fairmaire and †*Ceracyclus*, gen. nov., with two new species from the Cenomanian Burmese amber (Coleoptera, Scarabaeoidea). Les Cahiers Magellanes 37: 1–13.

Bousquet Y (2002) Additions and corrections to the world catalogue of genus-group names of Geadephaga (Coleoptera) published by Wolfgang Lorenz (1998). Folia Heyrovskyana Supplementum 9, 78 pp.

Bousquet Y (2016) Litteratura Coleopterologica (1758–1900): a guide to selected books related to the taxonomy of Coleoptera with publication dates and notes. ZooKeys 583: 1–776. https://doi.org/10.3897/zookeys.583.7084

Bousquet Y (2018) The dating of the fourth volume of Guillaume-Antoine Olivier’s “Entomologie, ou histoire naturelle des insectes”. ZooKeys 734: 137–148. https://doi.org/10.3897/zookeys.734.22901

Bousquet Y, Bouchard P (2013) The genera in the second catalogue (1833–1836) of Dejean’s Coleoptera collection. ZooKeys 282: 1–219. https://doi.org/10.3897/zookeys.282.4401

Bousquet Y, Bouchard P (2016) Catalogue of genus-group names in Alleculinae (Coleoptera: Tenebrionidae). The Coleopterists Society Monograph 14: 131–151. https://doi.org/10.1649/0010-065X-69.mo4.131

Bousquet Y, Bouchard P (2017) Status of the new genera in Gistel’s “Die Insecten-Doubletten aus der Sammlung des Herrn Grafen Rudolph von Jenison Walworth” issued in 1834. ZooKeys 698: 113–145. https://doi.org/10.3897/zookeys.698.14913

Bousquet Y, Bouchard P (2018a) Case 3744 – *Bidessus* Sharp, 1880 (Coleoptera, Dytiscidae, Bidessini): proposed conservation of usage by designation of *Dytiscus unistriatus* Goeze, 1777 as the type species. The Bulletin of Zoological Nomenclature 75: 32–35. https://doi.org/10.21805/bzn.v75.a009

Bousquet Y, Bouchard P (2018b) Case 3746 – Ripiphoridae Laporte, 1840 and *Ripiphorus* Bosc, 1791 (Insecta, Coleoptera): proposed conservation of usage by designating *Ripiphorus subdipterus* Fabricius, 1792 as the type species of *Ripiphorus* and proposed ruling that Laporte (1840) used the type genus *Ripiphorus* in the sense defined by the type species *Ripiphorus subdipterus*. The Bulletin of Zoological Nomenclature 75: 36–43. https://doi.org/10.21805/bzn.v75.a010

Bousquet Y, Thomas DB, Bouchard P, Smith AD, Aalbu RL, Johnston MA, Steiner Jr WE (2018) Catalogue of Tenebrionidae (Coleoptera) of North America. ZooKeys 728: 1–455. https://doi.org/10.3897/zookeys.728.20602

Branden C van den (1882) Gyrinides. Revue Coléoptérologique 1: 48.

Branden C van den (1884) Catalogue des coléoptères carnassiers aquatiques (Haliplidae, Amphizoïdae, Pelobiidae & Dytiscidae). P. Weissenbruch, Bruxelles, 118 pp.

Brendel E, Wickham HF (1890) The Pselaphidae of North America. Bulletin from the Laboratories of Natural History of the State University of Iowa 1 [1888–1890]: 216–304. [+ pls 6–9]

Britton EB (1987) A revision of the Australian chafers (Coleoptera: Scarabaeidae: Melolonthinae). Vol. 5. tribes Scitalini and Comophorinini. Invertebrate Taxonomy 1: 685–799.

Caldara R, Alonso-Zarazaga MA (2018) Case 3783 – *Orchestes* Illiger, 1798 (Insecta, Coleoptera, Curculionoidea, Curculionidae): proposed precedence over *Salius* Schrank, 1798. Bulletin of Zoological Nomenclature. 75: 247–250. https://doi.org/10.21805/bzn.v75.a050

Casey TL (1908) A revision of the tenebrionid subfamily Coniontinae. Proceedings of the Washington Academy of Sciences 10: 51–166. https://doi.org/10.5962/bhl.title.8935

Cerda M (1986) Lista sistemática de los cerambícidos chilenos (Coleoptera: Cerambycidae). Revista Chilena de Entomología 14: 29–39.

Chagnon G (1934) Contribution a l’étude des coléoptères de la Province de Québec (suite). Le Naturaliste Canadien 61: 309–319.

Chalande J (1884) Les lamellicornes français. Bulletin de la Société d’Histoire Naturelle de Toulouse 18: 43–121.

Chevrolat LAA (1847b) Promecotheca [p. 482]. In d’Orbigny C (Ed) Dictionnaire universel d’histoire naturelle… Tome dixième [Livraisons 109–120]. MM. Renard, Martinet et Cie., Paris, 760 pp.

Chûjô M (1939a) On the Japanese Ciidae (Coleoptera). Mushi 12: 1–10.

Chûjô M (1939b) Family Ciidae. In: Okada Y, Uchida T, Esaki T (Eds) Fauna Nipponica. Class Insecta: Coleopteroidea-Coleoptera. Volume X, Fascicle VIII, Number XIII. Sanseido, Tokyo, 63 pp. [cites article Chûjô (1939a) on page 58; in Japanese]

Colonnelli E (2013) New acts and comments. Curculionidae: Ceutorhynchinae. In: Löbl I, Smetana A (Eds) Catalogue of Palaearctic Coleoptera. Volume 8. Curculionoidea II. Brill, Leiden/Boston, 56–58.

Csiki E (1902) A bogarak systematikájáról (Az ujabb vizsgálatok eredményeinek ismertetése). III. Rovartani Lapok 9: 190–192. https://doi.org/10.3406/linly.1976.10251

Csiki E (1909c) Magyarország Buprestidái. Rovartani Lapok 16: 161–184.

Csiki E (1924) Pars 77. Serropalpidae. In Schenkling S (Ed) Coleopterorum Catalogus. Volume XVII. W. Junk, Berlin, 62 pp.

Dajoz R (1976) Les coléoptères Murmidiidae et Euxestidae de la faune paléarctique. Morphologie, biologie, systématique. Bulletin Mensuel de la Société Linnéenne de Lyon 45: 182–192.

Davie PJF, Guinot D, Ng PKL (2015) Systematics and classification of Brachyura. In: Castro P, Davie PJF, Guinot D, Schram FR, von Vaupe Klei JC (Eds) The Crustacea, complementary to the Traité de Zoologie, Volume 9 Part C-II. Brill, Koninklijke, 1049–1130. https://doi.org/10.1163/9789004190832_021

Della Beffa G (1915) I Coleotteri italiani nocivi alle piante coltivate. Sistematica – biologia – agraria. Verderi e C, Salsomaggiore, 68 pp.

Desbrochers des Loges J (1899) Faunule des Coléoptères de la France et de la Corse. Anthicidae (suite). Le Frelon 8: 17–32.

Doyen T, Tschinkel (1982) Phenetic and cladistic relationships among tenebrionid beetles (Coleoptera). Syst. Ent. 7: 127–183. https://doi.org/10.1111/j.1365-3113.1982.tb00129.x

Duméril A-M-C (1827) Rhipiphore, *Rhipiphorus* [pp. 374–375]. In: d’Orbigny C (Ed) Dictionnaire universel d’histoire naturelle... . Tome quarante-cinquième. MM. Renard, Martinet et Cie., Paris.

Duponchel PAJ (1844) Dadophora [pp. 574–575]. In: D’Orbigny C (Ed.) Dictionnaire universel d’histoire naturelle… Tome quatrième [Livraisons 43–47]. MM. Renard, Martinet et Cie, Paris, 752 pp.

Engel MS (2010) A primitive anobiid beetle in mid-Cretaceous amber from Myanmar (Coleoptera: Anobiidae). Alavesia 3: 31–34. https://doi.org/10.1016/j.cretres.2009.09.009

Fauvel A (1885) Rectifications au *Catalogus Coleopterorum Europae et Caucasi* Cicindelidae. Pythidae. Revue d’Entomologie 4: 174–187.

Fery H, Grygier MJ (2019) Comment (Case 3744) – Alternative proposals to conserve usage of *Bidessus* Sharp, 1880, and newly proposed suppression of *Dyticus parvulus* Müller, 1776 (Coleoptera, Dytiscidae, Bidessini). Bulletin of Zoological Nomenclature 76: 62–67. https://doi.org/10.21805/bzn.v76.a017

Fleutiaux E (1921) Études sur les Melasidae (Coleoptera-Serricornia). Sixíème partie. Annales de la Société Entomologique de Belgique 61: 169–192.

Gahan CJ (1902) Descriptions of additional species mentioned and figured in the accompanying paper. 2. Coleoptera Longicornia. Proceedings of the Zoological Society of London 2: 275–278.

Gené CG (1839) De quibusdam insectis Sardiniae novis aut minus cognitis. Fasciculus II. Memorie della Reale Accademia delle Scienze di Torino (serie seconda) 1: 43–84. [+ 2 pls]

Giebel C (1855) Literatur. Zeitschrift für die Gesammten Naturwissenschaften 5: 45–96.

Guérin-Méneville FE (1840) Note monographique sur le genre de coléoptères nommé *Alurnus*, par Fabricius, et sur quelques groupes voisins. Revue et Magasin de Zoologie Pure et Appliquée 1840: 330–334.

Guérin-Méneville FE (1857) Rhipiphoridum Coleopterorum familiae dispositio systematica; auctore A. Gerstaecker. Berlin. 1855, in-4o, avec 1 planche gravée. Revue et Magasin de Zoologie pure et appliquée (2e série) 9: 90–91.

Guignot F (1954) Quarantième note sur les hydrocanthares. Bulletin et Annales de la Société Entomologique de Belgique 90: 40–45.

Gusarov VI (2011) Case 3537. Athetini Casey, 1910 and Geostibina Seevers, 1978 (Insecta, Coleoptera, Staphylinidae, Aleocharinae): proposed conservation. The Bulletin of Zoological Nomenclature 68: 54–60. https://doi.org/10.21805/bzn.v68i1.a12

Gustafson GT, Miller KB (2013) On the family- and genus-series nomina in Gyrinidae Latreille, 1810 (Coleoptera, Adephaga). Zootaxa 3731: 77–105. https://doi.org/10.11646/zootaxa.3731.1.3

Hatch MH (1971) The beetles of the Pacific northwest. Part V: Rhipiceroidea, Sternoxi, Phytophaga, Rhynchophora, and Lamellicornia. University of Washington Publications in Biology 16: xiv + 662 pp.

Hayek CMF von (1983) The date of publication of the fourth volume of Silbermann’s *Revue Entomologigue*. Archives of Natural History 11: 207–208. https://doi.org/10.3366/anh.1983.11.2.207

Heller KM (1900) Neue Käfer von Celebes IV. Abhandlungen und Berichte des Königl. Zoologischen und Arthropologisch-Ethnographischen Museums zu Dresden 9(5): 1–46. [+ 1 pl]

Hernando C, Ribera I (2016) Family Limnichidae Erichson, 1846. In: Löbl I, Löbl D (Eds) Catalogue of Palaearctic Coleoptera. Vol. 3. Scarabaeoidea, Scirtoidea, Dascilloidea, Buprestoidea and Byrrhoidea. Revised and Updated Edition. Brill, Leiden/Boston, 607–610.

Horaninow PF (1834) Primae lineae systematis naturae, nexui naturali omnium evolutionique progressivae per nixus reascendentes superstructi. Karoli Krajanis, Petropoli, xiii + 142 + xxi (Index) + [1 (Errata)] + [2 (Explicatio schematis)] pp. [+ 1 pl.]

Horn GH (1881b) Notes on Elateridae, Cebrionidae, Rhipiceridae and Dascyllidae. Transactions of the American Entomological Society 9: 76–90. https://doi.org/10.2307/25076401

Horn GH (1883) Miscellaneous notes and short studies of North American Coleoptera. Transactions of the American Entomological Society 10 [1882–83]: 269–312. https://doi.org/10.2307/25076425

Iablokoff-Khnzorian SM (1985) Les Pythidae paléarctiques (Coleoptera). Deutsche Entomologische Zeitschrift (Neu Folge) 32: 193–229. https://doi.org/10.1002/mmnd.19850320129

ICZN [International Commission on Zoological Nomenclature] (2011a) Opinion 2272 (Case 3484) Nomiidae Gozis, 1875 (Insecta, Coleoptera): spelling emended to Nomiusidae to remove homonymy with Nomiinae Robertson, 1904 (Insecta, Hymenoptera). The Bulletin of Zoological Nomenclature 68: 147–149. https://doi.org/10.21805/bzn.v68i2.a2

ICZN [International Commission on Zoological Nomenclature] (2011b) Opinion 2287 (Case 3513) *Chaetosoma* Westwood, 1851, *Apodasya* Pascoe, 1863 and Chaetosomatidae Crowson, 1952 (Insecta, Coleoptera): usage conserved. The Bulletin of Zoological Nomenclature 68: 303–305. https://doi.org/10.21805/bzn.v68i4.a11

ICZN [International Commission on Zoological Nomenclature] (2011c) Opinion 2288 (Case 3517) Latridiidae Erichson, 1842 (Insecta, Coleoptera): precedence given over Corticariidae Curtis, 1829, and *Corticaria* Marsham, 1802: usage conserved by designation of *Corticaria ferruginea* Marsham, 1802 as the type species. The Bulletin of Zoological Nomenclature 68: 306–308. https://doi.org/10.21805/bzn.v68i4.a12

ICZN [International Commission on Zoological Nomenclature] (2012a) Opinion 2297 (Case 3514) Enhydrini Régimbart, 1882 (Insecta, Coleoptera): spelling emended to Enhydrusini to remove homonymy with Enhydrini Gray, 1825 (Mammalia, Mustelidae). The Bulletin of Zoological Nomenclature 69: 145–146. https://doi.org/10.21805/bzn.v69i2.a5

ICZN [International Commission on Zoological Nomenclature] (2012b) Opinion 2298 (Case 3519) *Eumolpus* Weber, 1801, *Chrysochus* Chevrolat in Dejean, 1836 and *Bromius* Chevrolat in Dejean, 1836 (Insecta, Coleoptera, Chrysomelidae): usage conserved. The Bulletin of Zoological Nomenclature 69: 147–149. https://doi.org/10.21805/bzn.v69i2.a6

ICZN [International Commission on Zoological Nomenclature] (2012c) Opinion 2299 (Case 3529) *Otiorhynchus* Germar, 1824 and *Loborhynchus* Schoenherr, 1823 (Insecta, Coleoptera): emendation of entries on the Official List of Generic Names in Zoology. The Bulletin of Zoological Nomenclature 69: 150–151. https://doi.org/10.21805/bzn.v69i2.a7

ICZN [International Commission on Zoological Nomenclature] (2012d) Opinion 2300 (Case 3530) Plinthini Lacordaire, 1863 (Insecta, Coleoptera) and *Plinthus* Germar, 1817 conserved by designation of *Curculio megerlei* Panzer, 1803 as the type species. The Bulletin of Zoological Nomenclature 69: 152–154. https://doi.org/10.21805/bzn.v69i2.a1

ICZN [International Commission on Zoological Nomenclature] (2012e) Opinion 2305 (Case 3537) Athetini Casey, 1910 and Geostibina Seevers, 1978 (Insecta, Coleoptera, Staphylinidae, Aleocharinae): family-group names conserved. The Bulletin of Zoological Nomenclature 69: 237–239. https://doi.org/10.21805/bzn.v69i3.a5

ICZN [International Commission on Zoological Nomenclature] (2014) Opinion 2344 (Case 3590) *Scarabaeus* Linnaeus, 1758, *Dynastes* MacLeay, 1819, Scarabaeinae Latreille, 1802 and Dynastinae MacLeay, 1819 (Insecta, Coleoptera, Scarabaeoidea): usage conserved. The Bulletin of Zoological Nomenclature 71: 257–258. https://doi.org/10.21805/bzn.v71i4.a1

ICZN [International Commission on Zoological Nomenclature] (2015a) Opinion 2366 (Case 3615) *Polybothris* Dupont, 1833 (Insecta, Coleoptera): spelling conserved. The Bulletin of Zoological Nomenclature 72: 235–236. https://doi.org/10.21805/bzn.v72i3.a5

ICZN [International Commission on Zoological Nomenclature] (2015b) Opinion 2370 (Case 3634) Omaliidae Handlirsch, 1904 (Insecta, Archaeorthoptera) and Xenopteridae Pinto, 1986 (Insecta, Megasecoptera): emended to Omaliaidae and Xenopteraidae respectively. The Bulletin of Zoological Nomenclature 72: 318–320. https://doi.org/10.21805/bzn.v72i4.a13

ICZN [International Commission on Zoological Nomenclature] (2017) Opinion 2377 (Case 3624) A proposal for the rejection of 38 names in Anthicidae (Coleoptera): approved. The Bulletin of Zoological Nomenclature 73: 65–69. https://doi.org/10.21805/bzn.v73i1.a8

ICZN [International Commission on Zoological Nomenclature] (2018) Opinion 2397 (Case 3402) Photinini LeConte, 1881 (Insecta, Coleoptera) and Photininae Giglio-Tos, 1915 (Insecta, Mantodea): resolution of homonymy between family-group names. The Bulletin of Zoological Nomenclature 74: 111–114. https://doi.org/10.21805/bzn.v74.a027

Jakobson GG (1914) Review critico-bibliographique: Coleopterorum Catalogus, auspiciis et auxilio W. Junk editus a S. Schenkling. Berlin, 8°, 1911–1913 [in Russian]. Russkoe Entomologicheskoe Obozrenie 13 (3–4) [1913]: 523–534.

Jeannel R (1950) Un elmide cavernicole du Congo Belge [Coleoptera Dryopidae]. Revue Française d’Entomologie 17: 168–172.

Kalshoven LGE (1959) Studies on the biology of Indonesian Scolytoidea 4. Data on the habits of Scolytidae. Second part. Tijdschrift voor Entomologie 102: 135–173.

Karasawa H (2003) *Mesostylus* Bronn and Roemer, 1852, a senior subjective synonym of *Protocallianassa* Beurlen, 1930 (Crustacea: Decapoda: Thalassinidea): reversal of precedence. Paleontological Research 7: 181–182. https://doi.org/10.2517/prpsj.7.181

Kaszab Z (1940) Revision der Tenebrioniden-Tribus Platyscelini (Col. Teneb.). Mitteilungen der Münchner Entomologischen Gesellschaft 30: 119–235.

King RL (1866) Description of *Anapestus kreusleri*: a species of Coleopterous insect inhabiting ants’ nests in South Australia. The Transactions of the Entomological Society of New South Wales 1: 316–318.

Kirejtshuk AG, Azar D, Telnov D (2008) [new taxa] In: Kirejtshuk AG, Azar D (Eds) New taxa of beetles (Insecta, Coleoptera) from Lebanese amber with evolutionary and systematic comments. Alavesia 2: 15–46.

Kirejtshuk AG, Bouchard P (2018) *Arhinops*, a new name for the genus *Arhina* Murray, 1876, non *Arhina* Agassiz, 1846 (Insecta: Diptera), and notes on the tribe Arhinopini nom.n. (Insecta: Coleoptera: Nitidulidae: Cryptarchinae). Caucasian Entomological Bulletin 14: 157–159. https://doi.org/10.23885/181433262018142-157159

Klausnitzer B (1995) Die Hirschkäfer: Lucanidae. 2. überarbeitete Auflage. Westarp Wissenschaften, Magdeburg, 109 pp.

Kolbe HJ (1883b) Ueber die madagascarischen Dytisciden des Königl. entomologischen Museums zu Berlin. Archiv für Naturgeschichte 49(1): 383–427. https://doi.org/10.5962/bhl.part.5845

Kolbe H (1912) Die tiergeographischen Verhältnisse der Scarabaeidengruppe der Phaeochroinen. Entomologische Rundschau 29: 153–155.

Kundrata R, Kubaczkova M, Prosvirov AS, Douglas HB, Fojtikova A, Costa C, Bousquet Y, Alonso-Zarazaga MA, Bouchard P (2019) World catalogue of the genus-group names in Elateridae (Insecta, Coleoptera). Part I: Agrypninae, Campyloxeninae, Hemiopinae, Lissominae, Oestodinae, Parablacinae, Physodactylinae, Pityobiinae, Subprotelaterinae, Tetralobinae. ZooKeys 839: 83–154. https://doi.org/10.3897/zookeys.839.33279

Latreille PA (1817) Cylidre, *Cylidrus*, Latr. Nouveau dictionnaire d’histoire naturelle appliquée aux arts, à l’agriculture, à l’économie rurale et domestique, à la médecine, etc. par une société de naturalistes et d’agriculteurs: nouvelle édition presqu’entièrement refondue et considérablement augmentée; avec des figures tirées des trois règnes de la nature. Tome IX. Déterville, Paris, 43–44.

Latreille PA (1819) Scraptie, *Scraptia* [pp. 437–438]. Nouveau dictionnaire d’histoire naturelle, appliquée aux arts, à l’agriculture, à l’économie rurale et domestique, à la médecine, etc. Par une société de naturalistes et d’agriculteurs. Nouvelle édition. Tome XXX. Déterville, Paris, 592 pp. [+ 6 pls] [1 May 1819]

Latreille PA (1828) Piméliaires. Pimeliariae. Ins. Dictionnaire classique d’histoire naturelle, par Messieurs Audouin, Isid. Bordon, Ad. Brongniart, De Candolle, Dandebard de Férussac, A. Desmoulins, Drapiez, Edwards, Flourens, Geoﬀroy de Saint-Hilaire, A. De Jussieu, Kunth, G. de Lafosse, Lamouroux, Latreille, Lucas fls, Presle-Duplessis, C. Prévost, A. Richard, Tiébaut de Berneaud, et Bory de Saint-Vincent. Ouvrage dirigé par ce dernier collaborateur, et dans lequel on a ajouté, pour le porter au niveau de la science, un grand nombre de mots qui n’avaient pu faire partie de la plupart des dictionnaires antérieurs. Tome treizième. PAN–PIV. Ray et Gravier [&] Baudouin Frères, Paris, 573–581.

Lawrence JF, Escalona E, Leschen RAB (2010c) Tenebrionoidea *Incertae sedis* [pp. 750–760]. Part 38. Coleoptera, beetles. Volume 2: Morphology and systematics (Elateroidea, Bostrichiformia, Cucujiformia partim). In: Leschen RAB, Beutel RG, Lawrence JF, Ślipiński SA (Eds) Handbook of zoology. Volume IV. Arthropoda: Insecta. W. de Gruyter, New York and Berlin, xiii + 786 pp. https://doi.org/10.1515/9783110911213.487

Lawrence JF, Kawashima I, Branham MA (2010a) Elateriformia *Incertae sedis* [pp. 162–177]. Part 38. Coleoptera, beetles. Volume 2: Morphology and systematics (Elateroidea, Bostrichiformia, Cucujiformia partim). In: Leschen RAB, Beutel RG, Lawrence JF, Ślipiński SA (Eds) Handbook of zoology. Volume IV. Arthropoda: Insecta. W. de Gruyter, New York and Berlin, xiii + 786 pp.

Lawrence JF, Reichardt H (1966) Revision of the genera *Gnostus* and *Fabrasia* (Coleoptera: Ptinidae). Psyche 73: 30–45. https://doi.org/10.1155/1966/89786

Lawrence JF, Ślipiński SA, Elgueta M (2010b) Promecheilidae Lacordaire 1859 [pp. 563–567]. Part 38. Coleoptera, beetles. Volume 2: Morphology and systematics (Elateroidea, Bostrichiformia, Cucujiformia partim). In: Leschen RAB, Beutel RG, Lawrence JF, Ślipiński SA (Eds) Handbook of zoology. Volume IV. Arthropoda: Insecta. W. de Gruyter, New York and Berlin, xiii + 786 pp. https://doi.org/10.1515/9783110911213.563

Legalov AA (2010) Phylogeny of the family Nemonychidae (Coleoptera) with descriptions of new taxa. Eurosian Entomological Journal 9: 457–473.

Lesne P (1894) Le genre *Dysides* Pert. (*Apoleon* Gorh. in part). Annales de la Société Entomologique de France 63: 18–21.

Lesne P (1926) Sur le genre *Lyprochelida* Fairm. (Tenebrionidae, Lypropini). Encyclopédie Entomologique (Série B) 1 (Coleoptera): 68.

Lopatin IK (1977) Leaf beetles (Chrysomelidae) of Central Asia and Kazakhstan [in Russian]. Nauka, Leningrad, 268 pp.

Lucas PH (1859) Animaux nouveaux ou rares recueillis pendant l’expédition dans les parties centrales de l’Amérique du Sud, de Rio de Janeiro à Lima, et de Lima au Para; exécutée par ordre du gouvernement Français pendant les années 1843 a 1847, sous la direction du Comte Francis de Castelnau. Entomologie [Livraison 28]. P. Bertrand, Paris, 113–160.

Mandl K (1954) Aedoeagus-Studien an Cicindeliden-Gattungen (Col.). Entomologische Arbeiten aus dem Museum G. Frey 5: 1–19.

Mannerheim CG von (1852) Zweiter Nachtrag zur Kaefer-Fauna der nord-amerikanischen Laender des russischen Reiches. Bulletin de la Société Impériale des Naturalistes de Moscou 25(2): 283–387.

Marseul S de (1864) Catalogue des coléoptères d’Europe et du bassin de la Méditéranée, par M. S.-A. de Marseul. Nov. 1843, in-12. L’Abeille Mémoires d’Entomologie 1: 25–33.

Matthews A (1886) Description of a new genus, and some new species of Corylophidae. The Entomologist’s Monthly Magazine 22: 224–228.

Matthews EG (1987) A guide to the genera of beetles of South Australia. Part 5 Polyphaga: Tenebrionoidea. South Australian Museum, Special Educational Bulletin No. 8, v + 67 pp.

Morrone JJ, Roig Juñent S (1995) The diversity of Patagonian weevils: An illustrated checklist of the Patagonian Curculionoidea (Insecta: Coleoptera). Ediciones L.O.L.A., Buenos Aires, 189 pp.

Naderloo R (2017) Atlas of crabs of the Persian Gulf. Springer, Cham, 290 pp. https://doi.org/10.1007/978-3-319-49374-9

Newton AF (2015) Cyrtoscydmini Schaufuss, 1889 replaced by Glandulariini Schaufuss, 1889 (Coleoptera: Staphylinidae: Scydmaeninae). The Coleopterists Bulletin 69: 758–759. https://doi.org/10.1649/0010-065X-69.4.758

Newton AF (2017) Nomenclatural and taxonomic changes in Staphyliniformia (Coleoptera). Insecta Mundi 0595: 1–52.

Ochs G (1926) Die Dineutini. 2. Tribus der Unterfamilien Enhydrinae Fam. Gyrinidae (Col.). A. Allgemeiner Teil. Entomologische Zeitschrift 40: 61–74.

Ochs G (1953) Gyrinidae (Coleoptera Adephaga). Exploration du Parc National de l’Upemba. Fascicle 16. Institut des Parcs Nationaux du Congo Belge, Bruxelles, 56 pp.

Odnosum VK (2010) Zhuki-Gorbatki (Coleoptera, Mordellidae). Fauna of Ukraine (in 40 volumes), Volume 19: Zhestkokrilie (Coleoptera), issue 9. Kiev, Naukova Dumka, 263 pp. [in Russian]

Ohaus F (1902) Beitrag zur Kenntniss der afrikanischen Popillien. Deutsche Entomologische Zeitschrift (Jahrgang 1901): 257–271.

Olivier E (1911) Révision des lampyrides. Revue Scientifique du Bourbonnais et du Centre de la France 24: 24–27, 39–58, 63–85, 98–112.

Panzer GWF (1796) Faunae insectorum Germanicae initia. Deutschlands Insecten. [Heft 37] Felsecker, Nürnberg. [24 pls + text]

Pascoe FP (1871) Notes on Coleoptera, with descriptions of new genera and species. Part I. The Annals and Magazine of Natural History (fourth series) 8: 345–361. https://doi.org/10.1080/00222937108696503

Perrier EJO (1893) Traité de zoologie. Première partie. Zoologie générale: protozoaires et phytozoaires; arthropodes. Avec 980 figures dans le texte. [Livraison 3]. G. Masson, Paris, 865–1344.

Perty M (1840) Allgemeine Naturgeschichte, als philosophische und Humanitätswissenschaft für Naturforscher, Philosophen und das höher gebildete Publikum. III. Band. [Lieferungen 6–7]. C. Fischer, Bern, 721–1119. [1841]

Pic M (1903) Contribution a l’étude générale des Hylophilidae. Annales de la Société Entomologique de France 72: 65–107.

Pic M (1912b) Quelques mots sur la classification des « Anobiides ». L’Échange, Revue Linnéenne 28: 53–55.

Pic M (1915b) Mélanges exotico-entomologiques. Seizième fascicule. Imprimerie Étienne Auclaire, Moulins, 24 pp.

Plisko JD (2013) A new family Tritogeniidae for the genera *Tritogenia* and *Michalakus*, earlier accredited to the composite Microchaetidae (Annelida: Oligochaeta). African Invertebrates 54: 69–92. https://doi.org/10.5733/afin.054.0107

Ponomarenko AG, Prokin AA (2015) Review of paleontological data on the evolution of aquatic beetles (Coleoptera). Paleontological Journal 49(13): 1383–1412. https://doi.org/10.1134/S0031030115130080

Porta A (1929) Fauna coleopterorum Italica. Vol. III. Diversicornia. Stabilimento Tipographico Piacentino, Piacenza, 466 pp.

Prena J (2018) An annotated inventory of the weevils (Coleoptera: Curculionoidea) described by Thomas Say. Bulletin of the Museum of Comparative Zoology 161: 323–401. https://doi.org/10.3099/MCZ161-09.1

Prudhomme de Borre A (1886) Catalogue des Trogides décrits jusqu’à ce jour, procédé d’un synopsis de leurs genres et d’une esquisse de leur distribution géographique. Annales de la Société Entomologique de Belgique 30: 54–82. https://doi.org/10.5962/bhl.part.26377

Raffray A (1898) Notes sur les psélaphides. Révision générique de la tribu des Euplectini. Descriptions d’espèces nouvelles. Revue d’Entomologie 17: 198–273.

Rambousek FJ (1907) Coleoptera Saviňský Alp. Časopis České Společnosti Entomologické 4: 36–41.

Reitter E (1874) Beitrag zur Kenntniss der japanesischen Cryptophagen. Verhandlungen der kaiserlich-königlichen Zoologisch-Botanischen Gesellschaft in Wien 24: 379–382.

Reitter E (1879) Verzeichniss der von H. Christoph in Ost-Sibirien gesammelten Clavicornier etc. Deutsche Entomologische Zeitschrift 23: 209–226. https://doi.org/10.1002/mmnd.48018790203

Reitter E (1909b) Die Süsswasserfauna Deutschlands: eine Exkursionsfauna. Heft 3 u. 4: Coleoptera. Gustav Fischer, Jena, 235 pp.

Ribeiro GC, Amorin DS (2002) A review of the genus *Elephantomyia* Osten Sacken in Brazil, with description of two new species (Diptera: Tipulomorpha, Limoniidae). Zootaxa 46: 1–16. https://doi.org/10.11646/zootaxa.46.1.1

Riley EG, Clark SM, Gilbert AJ (2001) New records, nomenclatural changes, and taxonomic notes for select North American leaf beetles (Coleoptera: Chrysomelidae). Insecta Mundi 15(1): 1–17.

Rose LJ (1983) Notes et rèflexion sur la classification et la nomenclature des coléoptères Cerambycidae de la faune de France (suite). Bulletin de la Société Entomologique de Mulhouse [juillet–septembre 1982]: 43–48.

Samouelle G (1819) The entomologist’s useful compendium; or an introduction to the knowledge of British insects, comprising the best means of obtaining and preserving them, and a description of the apparatus generally used; together with the genera of Linné, and the modern method of arranging the classes Crustacea, Myriapoda, spiders, mites and insects, from their affinities and structure, according to the views of Dr. Leach. Also an explanation of the terms used in entomology; a calendar of the times of appearance and usual situations of near 3,000 species of British insects; with instructions for collecting and fitting up objects for the microscope. Illustrated with twelve plates. Tomas Boys, London, 496 pp. [+ 12 pls] https://doi.org/10.5962/bhl.title.120094

Schawaller W (2011) Revision of the genera *Anaedus*, *Dichastops*, *Luprops* and *Sphingocorse* from South Africa and adjacent regions, with description of *Capeluprops* n. gen. (Coleoptera: Tenebrionidae: Lagriinae: Lupropini). Stuttgarter Beiträge zur Naturkunde A, (Neue Serie) 4: 269–288.

Scheerpeltz O (1925) Staphylinidae [pp. 323–447 except Steninae (pp. 349–357), Euaesthetinae (pp. 357–358) and *Staphylinus* (pp. 381–386)]. In: Winkler A (Ed.) Catalogus Coleopterorum regionis palaearcticae. I. A. Caraboidea, B. Palpicornia, Staphilinoidea, C. Diversicornia. Part 3. Albert Winkler, Wien, 241–368.

Schilder FA, Schilder M (1928) Die nahrung der Coccinelliden und ihre Beziehung zur Verwandtschaft der Arten. Arbeiten aus der Biologischen Reichsanstalt für Land- und Forstwirtschaft 16: 213–282.

Schilsky J (1888) Systematisches Verzeichnis der Käfer Deutschlands mit besonderer Berücksichtigung ihrer geographischen Verbreitung. Zugleich ein Käfer-Verzeichnis der Mark Brandenburg. Nicolai, Berlin, vi + 159 pp.

Schintlmeister A (2013) Notodontidae & Oenosandridae (Lepidoptera). Brill, Leiden & Boston, 608 pp. https://doi.org/10.1163/9789004259188

Schönfeldt H von (1887) Catalog der Coleopteren von Japan mit Angabe der bezüglichen Beschreibungen und der sicher bekannten Fundorte. Jahrbücher des Nassauischen Vereins für Naturkunde 40: 31–204.

Schönherr CJ (1840b) Genera et species curculionidum, cum synonymia hujus familiae; species novae aut hactenus minus cognitae, descriptionibus A Dom. Leonardo Gyllenhal, C. H. Boheman, et entomologis aliis illustratae. Tomus sextus. Pars prima. Supplementum continens. Roret, Paris, [2] + 474 pp.

Schweitzer CE, Feldmann RM (2012) Revision of Decapoda deposited in The Muséum national d’Histoire naturelle, Paris. Bulletin of the Mizunami Fossil Museum 38: 15–27.

Seidel M, Arriaga-Varela E, Fikáček M (2016) Establishment of Cylominae Zaitzev, 1908 as a valid name for the subfamily Rygmodinae Orchymont, 1916 with an updated list of genera (Coleoptera: Hydrophilidae). Acta Entomologica Musei Nationalis Pragae 56: 159–165.

Seidlitz G (1916b) Die letzten Familien der Heteromeren (Col.). (Fortsetzung). Deutsche Entomologische Zeitschrift 1916 (3/4): 313–344. https://doi.org/10.1002/mmnd.48019160305

Siegel M (1866) Versuch einer Käfer-Fauna Krains. Mitteilungen des Musealvereines für Krain 1: 89–209.

Ślipiński A, Escalona HE (2013) Australian longhorn beetles (Coleoptera: Cerambycidae). Volume 1. Introduction and subfamily Lamiinae. CSIRO Publishing, Melbourne, xviii + 484 pp. https://doi.org/10.1071/9781486300044

Stickney FS (1923) The head-capsule of Coleoptera with twenty-six plates. Illinois Biological Monographs 8(1): 1–104. https://doi.org/10.5962/bhl.title.49937

Streubel AV (1846) Das Thierreich geordnet nach seiner Organisation, als Grundlage der Naturgeschichte der Thiere und als Einleitung in die vergleichende Anatomie. Vom Freiherrn Georg v. Cuvier. Nach der zweiten, vermehrten, Ausgabe frei ins Deutsche übersetzt und durch Zusätze sowohl dem heutigen Standpunkte der Wissenschaft angepasst als auch für den Selbstunterricht eingerichtet. Erster Theil. G. Reimer, Berlin, xvii + [1] + 972 pp.

Tanner VM (1966) Rhynchophora beetles of the Nevada test site. Brigham Young University Science Bulletin, Biological Series 8(2): 1–32.

Telnov D (2004) Check-list of Latvian beetles (Insecta: Coleoptera). Second edition. Entomological Society of Latvia, Riga, 114 pp. https://doi.org/10.5962/bhl.part.7442

Téocchi P, Sudre J, Jiroux E (2010) Synonymies, diagnoses et bionomie de quelques Lamiaires africains (15^e^ note) (Coleoptera, Cerambycidae, Lamiinae). Les Cahiers Magellanes (NS) 1: 1–27.

Theobald W (1882) Burma, its people and productions; or, notes on the fauna, flora and minerals of Tenasserim, Pegu and Burma. By Rev. F. Mason. Vol. I. Geology, mineralogy and zoology. Rewritten and enlarged by W. Theobald. Stephen Austin & Sons, Hertford, xxiv + [1 (Errata)] + 560 pp.

Tomaszewska WK (2007) Family Endomychidae Leach, 1815 [except subfamily Merophysiinae]. In: Löbl I, Smetana A (Eds) Catalogue of Palaearctic Coleoptera. Volume 4. Elateroidea – Derodontoidea – Bostrichoidea – Lymexyloidea – Cleroidea – Cucujoidea. Apollo Books, Stenstrup, 559–568.

Villiers A (1984) Une nouvelle tribu de Cerambycidae: les Trigonarthrini (Coleoptera Cerambycidae). Bulletin de l’Institut Royal des Sciences Naturelles de Belgique (Entomologie) 55(10): 1–8.

Voss E (1930) Einige weitere Attelabiden und eine neue *Camarotus*-Art. Rhynihitinae [sic]. Sborník Entomologickeho Oddeleni Národniho Musea v Praze 8: 60–66.

Wang B, Ponomarenko AG, Zhang HC (2010) Middle Jurassic Coptoclavidae (Insecta: Coleoptera: Dytiscoidea) from China: a good example of mosaic evolution. Acta Geologica Sinica 84: 680–687. https://doi.org/10.1111/j.1755-6724.2010.00272.x

Wasmann ESJ (1909) Die psychischen Fähigkeiten der Ameisen. Mit einem Ausblick auf die vergleichende Tierpsychologie. 2. Aufl. Stuttgart, xi + 190 pp.

Wasmann ESJ (1929) Die Paussiden des baltischen Bernsteins und die Stammesgeschichte der Paussiden. Bernstein-Forschungen (Amber studies) 1: 1–110. https://doi.org/10.1002/mmnd.48019290102

Westerhauser J (1832) Latridii (Moderfresser) aus der Gegend von München. Faunus 1: 151–161.

Westwood JO (1842) Notice of a hitherto unobserved character distinctive of the sexes in certain Cetoniidae. The Annals and Magazine of Natural History 8: 338–341. https://doi.org/10.1080/03745484209442766

Wilcox JA (1954) Leaf beetles of Ohio (Chrysomelidae: Coleoptera). Ohio Biological Survey, Bulletin 43: 353–506.

Wilts EF, Bruns D, Fontaneto D, Ahlrichs WH (2012) Phylogenetic study on *Proales daphnicola* Thompson, 1892 (Proalidae) and its relocation to *Epiphanes* (Rotifera: Epiphanidae). Zoologischer Anzeiger 251: 180–196. https://doi.org/10.1016/j.jcz.2011.08.005

Winkler A (1915) Ein neuer blinder Tenebrionide aus der Krim. Wiener Entomologische Zeitung 34: 331–335. https://doi.org/10.5962/bhl.part.10623

Wollaston TV (1868) Coleoptera Hesperidum, being an enumeration of the coleopterous insects of the Cape Verde Archipelago. John van Voorst, London, xxxix + 285 pp. [1867] https://doi.org/10.5962/bhl.title.48651

Young FN (1980) Predaceous water beetles of the genus *Desmopachria* Babington: the subgenera with descriptions of new taxa (Coleoptera: Dytiscidae). Revista de Biologia Tropical 28: 305–321.

Zang R (1905) Passalidarum synonymia. Kritische Revision der von Kuwert und anderen Autoren aufgestellten Gattungen und Arten. Notes from the Leyden Museum 25: 221–232.

## Additions and corrections to appendices in Bouchard et al. (2011)

**Appendix 1**

Conservation of younger names using Reversal of Precedence (Art. 23.9). Cases listed in alphabetical order by family with their supporting references.

**Cerylonidae**

Supporting references for the conservation of Murmidiinae Jacquelin du Val, 1858 over Ceutocerinae Mannerheim, 1852 (Art. 23.9.2). The taxon name Ceutocerinae Mannerheim, 1852 has not been used as valid after 1899 to our knowledge.

Bouchard P, Bousquet Y, Davies AE, Alonso-Zarazaga MA, Lawrence JF, Lyal CHC, Newton AF, Reid CAM, Schmitt M, Ślipiński SA, Smith ABT (2011) Family-group names in Coleoptera (Insecta). ZooKeys 88: 1–972.

Bousquet Y, Bouchard P, Davies AE, Sikes DS (2013) Checklist of beetles (Coleoptera) of Canada and Alaska. Second edition. Pensoft, Sofia–Moscow, 402 pp.

Campbell JM (1991) Family Cerylonidae (cerylonid beetles). In: Bousquet Y (Ed.) Checklist of beetles of Canada and Alaska. 227–228.

Crowson RA (1981) The biology of the Coleoptera. Academic Press, New York, xii + 802 pp.

Dajoz R (1976) Les coléoptères Murmidiidae et Euxestidae de la faune paléarctique. Morphologie, biologie, systématique. Bulletin Mensuel de la Société Linnéenne de Lyon 45: 182–192.

Díaz AG, González JCO, Fernández MJL (2012) Proyecto fauna Ibérica. Coleoptera; Bothrideridae, Cerylonidae, Zopheridae. Arquivos Entomoloxicos 6: 74–76.

Dodelin B (2011) À propos des Cerylonidae de France et nouvelle découverte de *Philothermus evanescens* (Reitter) en Rhône-Alpes (Coleoptera). Bulletin Mensuel de la Société Linnéenne de Lyon 80: 53–59.

Elgueta M, Arriagada G (1989) Estado actual del conocimiento de los coleopteros de Chile (Insecta: Coleoptera). Revista Chilena de Entomología 17: 5–60.

Lawrence JF (1980) A new genus of Indo-Australian Gempylodini with notes on the constitution of the Colydiidae (Coleoptera). Australian Journal of Entomology 19: 293–310.

Lawrence JF (2016) Classification (families & subfamilies). In: Beutel RG, Leschen RAB (Eds) Coleoptera, beetles: morphology and systematics. Archostemata, Adephaga, Myxophaga, Polyphaga partim. Volume 1. 2^nd^ edition. Walter de Gruyter GmbH, Berlin/Boston, 13–22.

Lawrence JF, Beutel RG, Leschen RAB, Ślipiński A (2010) Changes in classification and list of families and subfamilies. In: Leschen RAB, Beutel RG. Lawrence JF (Eds) Coleoptera, beetles. Volume 2: morphology and systematics (Elateroidea, Bostrichiformia, Cucujiformia partim). Walter de Gruyter GmbH, Berlin/Boston, 1–7.

Lawrence JF, Newton Jr AF (1995) Families and subfamilies of Coleoptera (with selected genera, notes, references and data on family-group names). In: Pakaluk J, Ślipiński A (Eds) Biology, phylogeny, and classification of Coleoptera. Papers celebrating the 80^th^ birthday of Roy A. Crowson. Museum i Instytut Zoologii PAN, Warszawa, 779–1006.

Lawrence JF, Stephan K (1975) The North American Cerylonidae (Coleoptera: Clavicornia). Psyche 82: 131–166.

Pal TK, Lawrence JF (1986) A new genus and subfamily of mycophagous Bothrideridae (Coleoptera: Cucujoidea) from the Indo‐Australian region, with notes on related families. Journal of the Australian Entomological Society 25: 185–210.

Peck SB (2006) The beetles of the Galápagos Islands, Ecuador: evolution, ecology, and diversity (Insecta: Coleoptera). NRC Research Press, Ottawa, 313 pp.

Peck SB, Cook J, Hardy Jr JD (2002) Beetle fauna of the island of Tobago, Trinidad and Tobago, West Indies. Insecta Mundi 16: 9–23.

Peck SB, Thomas MC (1998) A distributional checklist of the beetles (Coleoptera) of Florida. Arthropods of Florida and Neighboring Land Areas, volume 16, 180 pp.

Robertson JA, Ślipiński A, Moulton M, Shockley FW, Giorgi A, Lord NP, Mckenna DD, Tomaszewska W, Forrester J, Miller KB, Whiting MF, Mchugh JV (2015) Phylogeny and classification of Cucujoidea and the recognition of a new superfamily Coccinelloidea (Coleoptera: Cucujiformia). Systematic Entomology 40: 745–778.

Robertson JA, Whiting MF, McHugh JV (2008) Searching for natural lineages within the Cerylonid series (Coleoptera: Cucujoidea). Molecular Phylogenetics and Evolution 46: 193–205.

Sen Gupta T, Crowson RA (1973) A review of the classification of Cerolynidae (Coleoptera, Clavicornia). Transactions of the Royal Entomological Society of London 124: 365–446.

Ślipiński A (1988) Revision of the Australian Cerylonidae (Coleoptera: Cucujoidea). Annales Zoologici 42: 1–74.

Ślipiński A (1990) Monograph of the world Cerylonidae (Coleoptera: Cucujoidea). Part I. Phylogeny and higher classification. Annali del Museo civico di Storia Naturale di Genova, 88: 1–273.

Ślipiński A (2007) Family Cerylonidae Billberg, 1820 [pp. 552–554]. In: Löbl I, Smetana A (Eds) Catalogue of Palaearctic Coleoptera. Volume 4. Elateroidea–Derodontoidea–Bostrichoidea–Lymexyloidea–Cleroidea–Cucujoidea. Apollo Books, Stenstrup, 935 pp.

Ślipiński A, Lawrence JF (2010) Cerylonidae Billberg, 1820. In: Leschen RAB, Beutel RG. Lawrence JF (Eds) Coleoptera, beetles. Volume 2: morphology and systematics (Elateroidea, Bostrichiformia, Cucujiformia partim). Walter de Gruyter GmbH, Berlin/Boston, 422–432.

Stephan KH (1989) The Bothrideridae and Colydiidae of America north of Mexico (Coleoptera; Clavicornia and Heteromera). Occasional Papers of the Florida State Collection of Arthropods 6, 65 pp.

Thomas MC (2002) 91. Cerylonidae Billberg, 1820 [pp. 363–365]. *In*: Arnett RH, Jr., Thomas MC, Skelley PE, Frank JH (Eds) (2002) American beetles. Volume 2. Polyphaga: Scarabaeoidea through Curculionoidea. CRC Press, Boca Raton, xiv + 861 pp.

**Chrysomelidae**

Supporting references for the conservation of Chalepini Weise, 1910 over Stenopodiini Horn, 1883: 290 and Microrhopalini Horn, 1883. The taxon names Stenopodiini Horn, 1883: 290 and Microrhopalini Horn, 1883 have not been used as valid after 1899 to our knowledge.

Bouchard P, Bousquet Y, Davies AE, Alonso-Zarazaga MA, Lawrence JF, Lyal CHC, Newton AF, Reid CAM, Schmitt M, Ślipiński SA, Smith ABT (2011) Family-group names in Coleoptera (Insecta). ZooKeys 88: 1–972.

Bousquet Y, Bouchard P, Davies AE, Sikes DS (2013) Checklist of beetles (Coleoptera) of Canada and Alaska. Second edition. Pensoft, Sofia–Moscow, 402 pp.

Butte JG (1969) Revision of the tribe Chalepini of America north of Mexico. IV. Genus *Sumitrosis* Butte (Coleoptera: Chrysomelidae). Joural of the New York Entomological Society 77: 12–30.

Casari SA (2005) Larva and pupa of *Metaxycera purpurata* (Chrysomelidae, Cassidinae, Chalepini). Iheringia, Série Zoologia 95: 373–376.

Casari SA, Teixeira EP (2004) Immatures of *Heterispa vinula* (Erichson) and *Physocoryna scabra* Guérin-Méneville (Coleoptera, Chrysomelidae, Cassidinae, Chalepini). Revista Brasileira de Entomologia 48: 473–480.

Eiseman CS (2014) New host records and other notes on North American leaf-mining Chrysomelidae (Coleoptera). The Coleopterists Bulletin 68: 351–359.

Eiseman CS (2015) Notes on leaf-mining Chrysomelidae (Coleoptera) in New England. The Coleopterists Bulletin 69: 453–458.

Ford EJ, Cavey JF (1985) Biology and Larval Descriptions of Some Maryland Hispinae (Coleoptera: Chrysomelidae). The Coleopterists Bulletin 39: 36–59.

Laplante S, Bousquet Y, Bélanger P, Chantal C (1991) Liste des espèces de coléoptères du Québec. Fabreries, supplément 6, 136 pp.

LeSage L (1991) Family Chrsyomelidae leaf beetles. In: Bousquet (Ed.) Checklist of beetles of Canada and Alaska. Agriculture Canada, Research Press, Ottawa, 301–323.

Majka CG, Chandler DS, Donahue CP (2011) Checklist of the beetles of Maine, USA. Empty Mirrors Press, Halifax, 328 pp.

Marucci RC, Moreira SG, Mendes SM (2017) First report of *Chalepus dorni* (Coleoptera: Chrysomelidae: Cassidinae) in maize crops of Minas Gerais, Brazil. Acta Biológica Colombiana 22: 246–248.

Meskens C, McKenna D, Hance T, Windsor D (2011) Host plant taxonomy and phenotype influence the structure of a Neotropical host plant-hispine beetle food web. Ecological Entomology 36: 480–489.

Naczi RFC, Staines CL (2011) Noteworthy Records of Hispines from Belize (Coleoptera: Chrysomelidae). Insecta Mundi 0190: 1–6.

Peck SB (2005) A checklist of the beetles of Cuba with data on distributions and bionomics (Insecta: Coleoptera). Arthropods of Florida and Neighboring Land Areas, volume 18, 241 pp.

Peck SB, Thomas MC (1998) A distributional checklist of the beetles (Coleoptera) of Florida. Arthropods of Florida and Neighboring Land Areas, volume 16, 180 pp.

Riley EG, Clark SM, Flowers RW, Gilbert AJ (2002) Chrysomelidae Latreille 1802. In: Arnett Jr RH, Thomas MC, Skelley PE, Frank JH (Eds) American beetles. Volume 2. Polyphaga: Scarabaeoidea through Curculionoidea. CRC Press, Boca Raton, 617–691.

Riley EG, Clark SM, Gilbert AJ (2001) New records, nomenclatural changes, and taxonomic notes for select North American leaf beetles (Coleoptera: Chrysomelidae). Insecta Mundi 15(1): 1–17.

Riley E, Enns WR (1979) An annotated list of Missouri leaf beetles (Coleoptera: Chrysomelidae). Transactions of the Missouri Academy of Science 13: 53–83.

Sikes DS (2004) The beetle fauna of Rhode Island: an annotated checklist. The Biota of Rhode Island 3 1–296.

Staines CL (2002) The New World tribes and genera of hispines (Coleoptera: Chrysomelidae: Cassidinae). Proceedings of the Entomological Society of Washington 104: 721–784.

Staines CL (2010) Nomenclatural notes on Chalepini and Sceloenopliini (Coleoptera: Chrysomelidae: Cassidinae). Insecta Mundi 0122: 1–2.

Staines CL (2011) Hispines (Chrysomelidae, Cassidinae) of La Selva Biological Station, Costa Rica. ZooKeys 157: 45–65.

Staines CL, Zamorano L (2012) Two new genera of hispines (Coleoptera: Chrysomelidae: Cassidinae) from Ecuador. Insecta Mundi 0232: 1–6.

Virkki N, Santiago-Blay JA, Riley EG (1992) Chromosomes of Puerto Rican Hispinae and Cassidinae (Coleoptera: Chrysomelidae). The Coleopterists Bulletin 46: 29–42.

Webster RP, LeSage L, DeMerchant I (2012) New Coleoptera records from New Brunswick, Canada: Megalopodidae and Chrysomelidae. ZooKeys 179: 321–348.

**Tenebrionidae**

Supporting references for the conservation of *Anomalipus* Guérin-Méneville, 1831 over *Heteroscelis* Latreille, 1828 (Art. 23.9.2). The taxon name *Heteroscelis* Latreille, 1828 has not been used as valid after 1899 to our knowledge.

Banaszkiewicz M (2006) Comparative study of female genitalia in Pedinini (sensu Iwan 2004) (Coleoptera: Tenebrionidae: Pedinini), with notes on the classification. Annales Zoologici 56: 59–77.

Barker JF (1983) Towards a biogeography of the Kalahari; part 1, to which region does the Kalahari belong? Botswana Notes and Records 15: 85–91.

Bouchard P (Ed) (2014) The book of beetles: A life-size guide to six hundred of nature’s gems. With contributions from Bouchard P, Bousquet Y, Carlton C, Chamorro ML, Escalona HE, Evans AV, Konstantinov A, Leschen RAB, Le Tirant S, Lingafelter SW. University of Chicago Press. Chicago, 656 pp.

Carl M (1994) Neue und bemerkenswerte Tenebrionidae aus dem Naturhistorischen Museum Wien, gesammelt von W. Kühnelt im Jahre 1964 in Namibia (Insecto: Coleoptera). Annalen des Naturhistorischen Museums in Wien (Serie B für Botanik und Zoologie) 96: 343–348.

Endrödy-Younga S (1980) The concept of heteromorph speciation. Consequences of speciation in homomorph and heteromorph populations. Annals of the Transvaal Museum 32: 241–247.

Endrödy-Younga S (1988) Revision of the genus *Anomalipus* Latreille, 1846 (Coleoptera, Tenebrionidae, Platynotini). Transvaal Museum Monograph No. 6, 129 pp.

Endrödy-Younga S (2000) Revision of the subtribe Gonopina (Coleoptera: Tenebrionidae, Opatrinae, Platynotini). Annals of the Transvaal Museum 37: 1–54.

Endrödy-Younga S, Tschinkel W (1993) Estimation of population size and dispersal in *Anomalipus mastodon* Fåhraeus, 1870 (Coleoptera: Tenebrionidae: Platynotini). Annals of the Transvaal Museum 36: 21–30.

Iwan D (2000) Revision of the trigonopoid Platynotina from South Africa. Part VII. Genera *Bantodemus* Koch, 1955 and *Parabantodemus* gen. nov. (Coleoptera: Tenebrionidae: Platynotini). Genus 11: 235–350.

Iwan D (2002) Catalogue of the world Platynotini (Coleoptera: Tenebrionidae). Genus 13: 219–323.

Iwan D (2002) Generic classification of the tribe Platynotini (Coleoptera: Tenebrionidae), with notes on phylogeny. Annales Zoologici 52: 1–149.

Iwan D (2004) A comparative study of male genitalia in Opatrinae sensu Medvedev (1968) (Coleoptera: Tenebrionidae), with notes on the reinterpreted tribal classification. Part II. Annales Zoologici 54: 735–765.

Iwan D (2010) Insecta Coleoptera Tenebrionidae Pedinini Platynotina. Faune de Madagascar 93, 178 pp.

Iwan D, Banaszkiewicz M (2005) Larvae of the genus *Anomalipus* Latreille, 1846 (Coleoptera: Tenebrionidae). Annales Zoologici 55: 375–381.

Iwan D, Bečvář S (2000) Description of the early stages of *Anomalipus plebejus plebejulus* (Coleoptera: Tenebrionidae) from Zimbabwe with notes on the classification of the Opatrinae. European Journal of Entomology 97: 403–412.

Iwan D, Kamiński MJ (2014) Taxonomy of the genus *Schelodontes* Koch, 1956 with a key to species (Coleoptera: Tenebrionidae: Platynotina). Insect Systematics & Evolution 45: 159–179.

Iwan D. (2008) Description of the larva of *Anchophthalmus* Gerstaecker, 1854 (Coleoptera: Tenebrionidae: Pedinini), with a key to larvae of Platynotina. African Entomology 16: 287–295.

Jagersbacher-Baumann J, Ebermann E (2012) Fungal spore transfer and intraspecific variability of a newly desccribed African soil mite (Heterostigmata, Scutacaridae, Heterodispus). Zoologischer Anzeiger 251: 101–114.

Kamiński MJ, Kanda K, Lumen R, Smith AD, Iwan D (2019) Molecular phylogeny of Pedinini (Coleoptera, Tenebrionidae) and its implications for higher-level classifcation. Zoological Journal of the Linnean Society 185: 77–97.

Kamiński MJ, Lumen R, Kubicz M, Kanda K, Iwan D (2019) Immature stages of beetles representing the ’Opatrinoid’ clade (Coleoptera: Tenebrionidae): an overview of current knowledge of the larval morphology. Zoomorphology 138: 1–22.

Merkl O, Grabant A, Soltész Z (2015) Type Catalogue of darkling beetles (Tenebrionidae) preserved in the Hungarian Natural History Museum. Hungarian Natural History Museum, Budapest, 735 pp.

Peña G LE (1985) Nota sobre tenebrionidos (Coleoptera: Tenebrionidae). Revista Chilena de Entomologia 12: 225.

Robiche G (2010) Les *Anomalipus* Latreille, 1846 de la région centrale du Mozambique. Description d’une nouvelle espèce (Coleoptera, Tenebrionidae). Lambillionea 110 (Numéro 2, Tome 2): 237–240.

Scholtz CH, Holm E (Eds) (2008) Insects of Southern Africa. Second edition. Protea Book House. Pretoria, x + 502 pp.

Schulze L (1978) The Tenebrionidae of southern Africa, XLV. Description of some larvae of the subgenera *Gonopus* and *Agonopus* of the genus *Gonopus* (Coleoptera). Annals of the Transvaal Museum 31: 1–16.

**Appendix 2**

Coleoptera family-group name changes required based on the Principle of Priority. The action that has been taken to fix the problem, or recommendation for future work, is mentioned for each case. Cases listed in alphabetical order by family.

Add the following entries:

**Table:**

Family	Change from:	Change to:	Action	Reference
Bostrichidae	Trogoxylini Lesne, 1921	Tristariini Lesne, 1921	An application to the Commission is needed to conserve usage of the well-established name Trogoxylini Lesne, 1921	
Cerambycidae	Ancitini Aurivillius, 1917	Hebesecini Pascoe, 1871	An application to the Commission is needed to conserve usage of the well-established name Ancitini Aurivillius, 1917	
Chrysomelidae	Uroplatini Weise, 1910	Octotomini Horn, 1883	An application to the Commission is needed to conserve usage of the well-established name Uroplatini Weise, 1910	
Curculionidae	Tropiphorini Marseul, 1863	Leptopini Lacordaire, 1863	An application to the Commission is needed to conserve usage of the well-established name Tropiphorini Marseul, 1863	
Elateridae	Eudicronychinae Girard, 1971	Dicronychidae Schwarz, 1897	An application to the Commission is needed to suppress the older name because it is based on a misidentified type genus (Art. 65.2.1)	
Endomychidae	Anamorphinae Strohecker, 1953	Symbiotinae Joy, 1932	An application to the Commission is needed to conserve usage of the well-established name Anamorphinae Strohecker, 1953	
Eucnemidae	Macraulacinae/-ini Fleutiaux, 1923	Fornacini Beaulieu, 1919 and Dromaeolini Beaulieu, 1919	An application to the Commission is needed to conserve usage of the well-established names Macraulacinae/-ini Fleutiaux, 1923	
Laemophloeidae	Laemophloeidae Ganglbauer, 1899	Nartheciidae Casey, 1890	An application to the Commission is needed to conserve usage of the well-established name Laemophloeidae Ganglbauer, 1899	
Limnichidae	Thaumastodinae Champion, 1924	Pseudeucinetinae Csiki, 1924	An application to the Commission is needed to conserve usage of the well-established name Thaumastodinae Champion, 1924	
Meloidae	Lyttini Streubel, 1846	Cantharini Latreille, 1802	An application to the Commission is needed to suppress the older name because it is based on a misidentified type genus (Art. 65.2.1)	
Scarabaeidae	Pachycnemina Laporte, 1840	Lepitrichina Perty, 1840	Reversal of Precedence or an application to the Commission is needed to conserve usage of the well-established name Pachycnemina Laporte, 1840	

Delete the following entries:

**Table:**

Family	Change from:	Change to:	Action	Reference
Elateroidea/-idae	Elateroidea/-idae Leach, 1815	Cebrionoidea/-idae Latreille, 1802	An application to the Commission will be sent to conserve usage of the well-established names Elateroidea/-idae Leach, 1815	P. J. Johnson (in Lawrence and Newton 1995, pers. comm. 2009)
Latridiidae	Latridiidae Erichson, 1842	Corticariidae Curtis, 1829	An application to the Commission was submitted by Bousquet et al. (2010) to conserve usage of the well-established name Latridiidae Erichson, 1842	
Meloidae	Lyttini Solier, 1851	Cantharini Latreille, 1802	An application to the Commission is needed to suppress the older name because it is based on a misidentified type genus (Art. 65.2.1)	

## Appendix 3

Coleoptera family-group name changes required based on the Principle of Homonymy. The action that has been taken to fix the problem, or recommendation for future work, is mentioned for each case. Cases listed in alphabetical order by family.

Add the following entries:

**Table:**

Family	Coleoptera name:	Homonym:	Type genera	Action	Reference
Anthribidae	Meconemini Pierce, 1930	Meconemini Burmeister, 1838 [Orthoptera]	different	An application to the Commission is needed to remove the homonymy (Art. 55.3.1)	
Byrrhidae	Microchaetini Paulus, 1973	Microchaetini Beddard, 1895 [Oligochaeta]	different	An application to the Commission is needed to remove the homonymy (Art. 55.3.1)	
Cantharidae	Ichthyurini Champion, 1915	Ichthyurinae Packard, 1895 [Lepidoptera]	different	An application to the Commission is needed to remove the homonymy (Art. 55.3.1)	
Carabidae	Homopterinae Wasmann, 1920	Homopterinae Boisduval, 1852 [Lepidoptera]	different	An application to the Commission is needed to remove the homonymy (Art. 55.3.1)	
Carabidae	Glyptini Horn, 1881	Glyptini Cushman and Rohwer, 1920 [Hymenoptera]	different	An application to the Commission is needed to remove the homonymy (Art. 55.3.1)	
Catiniidae	Catiniidae Ponomarenko, 1968	Catiniidae Bocquet and Stock, 1957 [Crustacea]	different	An application to the Commission is needed to remove the homonymy (Art. 55.3.1)	
Chrysomelidae	Chorinini Weise, 1923	Chorininae Dana, 1851 [Crustacea]	different	An application to the Commission is needed to remove the homonymy (Art. 55.3.1)	
Chrysomelidae	Gastrophysina Kippenberg, 2010	Gastrophysini Harting, 1864 [Pisces]	different	An application to the Commission is needed to remove the homonymy (Art. 55.3.1)	
Cleridae	Trichodidae Streubel, 1839	Trichodina Maitland, 1851 [Protozoa]	different	An application to the Commission is needed to remove the homonymy (Art. 55.3.1)	
Coccinellidae	Monocorynini Miyatake, 1988	Monocoryninae Rees, 1956 [Cnidaria]	different	An application to the Commission is needed to remove the homonymy (Art. 55.3.1)	
Elateridae	Limoniina Jakobson, 1913	Limoniidae Speiser, 1909 [Diptera]	different	An application to the Commission is needed to remove the homonymy (Art. 55.3.1)	
Eucnemidae	Epiphanini Muona, 1993	Epiphanidae Harring, 1913 [Rotifera]	different	An application to the Commission is needed to remove the homonymy (Art. 55.3.1)	
Passalidae	Gonatinae Kuwert, 1891	Gonatidae Hoyle, 1886 [Cephalopoda]	different	An application to the Commission is needed to remove the homonymy (Art. 55.3.1)	
Scarabaeidae	Aplonychidae H. C. C. Burmeister, 1855	Aplonychini De Stefani, 1908 [Diptera]	different	An application to the Commission is needed to remove the homonymy (Art. 55.3.1)	
Staphylinidae	Tychini Raffray, 1904	Tychinae Dana, 1851 [Crustacea]	different	An application to the Commission is needed to remove the homonymy (Art. 55.3.1)	
Staphylinidae	Ocypodina Hatch, 1957	Ocypodidae Rafinesque, 1815 [Crustacea]	different	An application to the Commission is needed to remove the homonymy (Art. 55.3.1)	
Trogossitidae	Decamerini Crowson, 1964	Decameridae Rasmussen, 1978 [Echinodermata]	different	An application to the Commission is needed to remove the homonymy (Art. 55.3.1)	

Delete the following entries:

**Table:**

Family	Coleoptera name:	Homonym:	Type genera	Action	Reference
Carabidae	Nomiini Gozis, 1875	Nomiidae Robertson, 1904 [Hymenoptera]	different	An application was submitted to the Commission to emend the stem of the beetle family-group name	Engel and Bouchard (2009)
Cleroidea	Chaetosomatidae Crowson, 1952	Chaetosomatidae Claus, 1872 [Nematoda]	same, Coleoptera genus older	An application to the Commission was submitted by YB and PB to conserve the Coleoptera name	
Gyrinidae	Enhydrini Régimbart, 1882	Enhydrini Gray, 1825	different	An application was recently submitted to the Commission by Özdikmen and Darilmaz (2010) to remove the homonymy	
Lampyridae	Photinini LeConte, 1881	Photininae Giglio-Tos, 1915 [Mantodea]	different	An application to the Commission was submitted by Svenson and Branham (2007) to remove the homonymy (Art. 55.3.1)	

**Appendix 4**

**Table:** Coleoptera names as they appear on the Official List of Family-Group Names in Zoology. Cases listed in alphabetical order by family.

Family	Name on Official List	Opinion(s) or Direction	Reference
Carabidae	Nomiusidae Gozis, 1875	2272	ICZN 2011a
Chaetosomatidae	Chaetosomatidae Crowson, 1952	2287	ICZN 2011b
Curculionidae	Plinthini Lacordaire, 1863	2300	ICZN 2012d
Gyrinidae	Enhydrusini Régimbart, 1882	2297	ICZN 2012a
Lampyridae	Photinini LeConte, 1881	2397	ICZN 2018
Latridiidae	Corticariidae Curtis, 1829	2288	ICZN 2011c
Latridiidae	Latridiidae Erichson, 1842	2288	ICZN 2011c
Scarabaeidae	Dynastidae MacLeay, 1819	2344	ICZN 2014
Scarabaeidae	Scarabaeidae Latreille, 1802	2344	ICZN 2014
Staphylinidae	Athetini Casey, 1910	2305	ICZN 2012e
Staphylinidae	Geostibina Seevers, 1978	2305	ICZN 2012d
Staphylinidae	Omaliidae MacLeay, 1825	2370	ICZN 2015b

**Appendix 5**

**Table:** Coleoptera type genus names as they appear on the Official List of Generic Names in Zoology. Cases listed in alphabetical order by family.

Family	Name on Official List	Opinion(s)	Reference(s)
Anthicidae	*Anthicus* Paykull, 1798	2377	ICZN 2017
Anthicidae	*Microhoria* Chevrolat, 1877	2377	ICZN 2017
Buprestidae	*Polybothris* Dupont, 1833	2366	ICZN 2015a
Carabidae	*Nomius* Laporte, 1835	2272	ICZN 2011a
Cerambycidae	*Apodasya* Pascoe, 1863	2287	ICZN 2011b
Chaetosomatidae	*Chaetosoma* Westwood, 1851	2287	ICZN 2011b
Chrysomelidae	*Bromius* Chevrolat, 1836	2298	ICZN 2012b
Chrysomelidae	*Eumolpus* Weber, 1801	2298	ICZN 2012b
Curculionidae	*Otiorhynchus* Germar, 1822 [year emended]	2299	ICZN 2012c
Curculionidae	*Plinthus* Germar, 1817	2299	ICZN 2012d
Lampyridae	*Photinus* Laporte, 1833	2397	ICZN 2018
Latridiidae	*Corticaria* Marsham, 1802	2288	ICZN 2011c
Latridiidae	*Latridius* Herbst, 1793	2288	ICZN 2011c
Scarabaeidae	*Dynastes* MacLeay, 1819	2344	ICZN 2014
Scarabaeidae	*Scarabaeus* Linnaeus, 1758	2344	ICZN 2014
Staphylinidae	*Callicerus* Gravenhorst, 1802	2305	ICZN 2012e
Staphylinidae	*Omalium* Gravenhorst, 1802	2370	ICZN 2015b

**Appendix 6**

Summary of cases involving Coleoptera family-group names and/or their type genera awaiting a ruling by the Commission. Cases listed in alphabetical order by family.

Replace the cases listed in Bouchard et al (2011: 894–895) with:

**Chrysomelidae**

An application to conserve *Eupales* Lefèvre, 1885 and Eupalini Verma et al. 2005 was submitted by Jolivet and Verma (Bulletin of Zoological Nomenclature 2009: 204; see notice of “New applications to the Commission”) but this Case (number 3498) was never published (ICZN secretariat pers. comm. 2019).

**Dytiscidae**

An application to conserve usage of the genus *Bidessus* Sharp, 1880 was submitted by Bousquet and Bouchard (2018a). An alternative request to the Commission was subsequently published by Fery and Grygier (2019).

**Eucnemidae**

An application to conserve usage of Eucnemidae Eschscholtz, 1829 over Melasidae Fleming, 1821 was submitted by Muona (Bulletin of Zoological Nomenclature 1994: 185; see notice of “New applications to the Commission”) but this Case (number 2938) was never published (ICZN secretariat pers. comm. 2019).

**Hybosoridae**

Ballerio (Bulletin of Zoological Nomenclature 2018: 3; see “Notice of New Applications to the Commission (Cases 3753–3772)”) submitted an application to conserve usage of *Acanthocerus* W. S. MacLeay, 1819 (now *Ceratocanthus* A. White, 1842). This Case (number 3766) has not been published yet.

**Ripiphoridae**

A request was submitted to the Commission by Bousquet and Bouchard (2018b) to conserve usage of Ripiphoridae Laporte, 1840 and its type genus *Ripiphorus* Bosc, 1791.

**Tenebrionidae**

Bousquet and Bouchard (Bulletin of Zoological Nomenclature 2018: 206; see “Notice of New Applications to the Commission (Cases 3782–3787)”) submitted an application to conserve usage of *Cnodalon* Latreille, 1797. This Case (number 3784) has not been published yet.

**Curculionidae**

Caldara and Alonso-Zarazaga (2018) submitted an application to the Commission to conserve usage of *Orchestes* Illiger, 1798 by giving it precedence over *Salius* Schrank, 1798.

